# Significance of research on natural products from marine-derived *Aspergillus* species as a source against pathogenic bacteria

**DOI:** 10.3389/fmicb.2024.1464135

**Published:** 2024-09-19

**Authors:** Bin Wang, Jin Cai, Longtao Huang, Yonghao Chen, Ruoxi Wang, Mengyao Luo, Meng Yang, Mohan Zhang, Nasihat, Guangying Chen, Guolei Huang, Caijuan Zheng

**Affiliations:** ^1^Key Laboratory of Tropical Medicinal Resource Chemistry of Ministry of Education, College of Chemistry and Chemical Engineering, Hainan Normal University, Haikou, China; ^2^Key Laboratory of Tropical Medicinal Plant Chemistry of Hainan Province, Haikou, China

**Keywords:** marine-derived, *Aspergillus* sp., secondary metabolites, antibacterial activity, antimicrobial resistance

## Abstract

Bacterial infections pose a significant clinical burden on global health. The growing incidence of drug-resistant pathogens highlights the critical necessity to identify and isolate bioactive compounds from marine resources. Marine-derived fungi could provide novel lead compounds against pathogenic bacteria. Due to the particularity of the marine environment, *Aspergillus* species derived from marine sources have proven to be potent producers of bioactive secondary metabolites and have played a considerable role in advancing drug development. This study reviews the structural diversity and activities against pathogenic bacteria of secondary metabolites isolated from marine-derived *Aspergillus* species over the past 14 years (January 2010–June 2024), and 337 natural products (including 145 new compounds) were described. The structures were divided into five major categories—terpenoids, nitrogen-containing compounds, polyketides, steroids, and other classes. These antimicrobial metabolites will offer lead compounds to the development and innovation of antimicrobial agents.

## 1 Introduction

Bacterial infections pose a significant clinical burden on global health (Xuan et al., 2023; Wallis et al., 2023). An estimated 7.7 million deaths are attributed to bacterial infections each year (Okeke et al., 2024; Ikuta et al., 2022). For example, *Staphylococcus aureus*, a frequent colonizer of the human population and one of the foremost opportunistic bacterial pathogens of humans, was associated with more than 1 million deaths in 2019. *Staphylococcus aureus* caused significant morbidity and mortality globally (Howden et al., 2023). Additionally, four additional pathogens (*Escherichia coli*, *Streptococcus pneumoniae*, *Klebsiella pneumoniae*, and *Pseudomonas aeruginosa*) were also associated with more than 0.5 million deaths each in 2019 (Ikuta et al., 2022). Deaths related to bacteria would rank as the second leading cause of death globally. Furthermore, antimicrobial resistance (AMR) remains a global threat. AMR posed a significant global public health threat owing to the rapid global acceleration of resistance in microorganisms. This trend limited the effectiveness of preventing and treating infections caused by viruses, bacteria, and parasites (Charani et al., 2023; Haenni et al., 2022; de Alcântara Rodrigues et al., 2020). A global surveillance report by the World Health Organization (WHO) identified the severe economic effects of AMR (de Alcântara Rodrigues et al., 2020). For instance, the estimated annual expense for the US healthcare system alone ranges from $21 to $34 billion. Beyond the health sector, AMR was projected to cause a decline in actual gross domestic product (GDP) of 0.4 to 1.6% (Gow et al., 2022; Jin et al., 2023). Consequently, the lack of new antimicrobial drugs to replace those that become ineffective underscored the urgent need to preserve the efficacy of existing drugs (Prestinaci et al., 2015). The increasing challenge of AMR highlighted the importance of marine microbial resources as crucial assets in developing new antimicrobial drugs (Alahmari et al., 2022; Carroll et al., 2024). Marine microorganisms, through long-term adaptation to extreme environments, have evolved unique metabolic pathways capable of synthesizing various structurally diverse antimicrobial compounds (Pinedo-Rivilla et al., 2022; Hai et al., 2021), such as marine sponge-derived terpenoid 13-(*E*)-geoditin A (Chen B. et al., 2022), marine coral-derived steroid lobocaloid B (Zhu et al., 2024), ascidian lactone prunolide C (Holland et al., 2022), mangrove sediments polyketone stemphone C (Cai et al., 2023). Thus, marine microorganism resources emerged as an essential source of structurally novel and antimicrobial natural products (Jeewon et al., 2023; Yurchenko et al., 2021; Han et al., 2023; Xu et al., 2022).

Genus *Aspergillus* has been considered one of the most significant general fungi, and representatives have been found in almost all aerobic environments, such as plants, soil, marine life, and submarine sediments (Ibrahim et al., 2023; Sun et al., 2022). Several metabolites of *Aspergillus* have been proven to possess valuable activities, such as aspergillomarasmine A from *Aspergillus versicolor* surmount metallo-*β*-lactamase antibiotic resistance, and Simvastatin, from *Aspergillus terreus* with a critical blood-lipid-lowering medicine, as a potential drug against *S. aureus* biofilm (King et al., 2014; Graziano et al., 2015). Furthermore, marine-derived *Aspergillus* fungi, which lived the diverse and hostile environments, produced a variety of structurally novel and antibacterial chemical compounds, and a significant proportion of these compounds were secondary metabolites with antimicrobial activity (Orfali et al., 2021; Li H. H. et al., 2023; Wang and Ding, 2018; Lee et al., 2013), such as marine-derived fungus *Aspergillus ustus* polyketone stromemycin B (Xue et al., 2024), marine gorgonian-derived fungus *Aspergillus sclerotiorum* alkaloid sclerotiamide L (Meng et al., 2022), marine coral-derived fungus *Aspergillus hiratsukae* terpene chevalone H (Chen X. Y. et al., 2022), marine sediment-derived fungus *A. terreus* lactone butyrolactone I (Bao et al., 2021). Moreover, a series of outstanding reviews on marine-derived *Aspergillus* fungi has been published. In 2013, Lee et al. reviewed the bioactive secondary metabolites of *Aspergillus* derived from marine sources. In 2018, Wang et al. conducted a review of 232 new bioactive metabolites of *Aspergillus* in the marine environment from 2006 to 2016 and categorized their bioactivity and chemical structures (Wang and Ding, 2018). In 2020, Xu et al. summarized the structural diversity and biological activity of 130 heterocyclic alkaloids produced by *Aspergillus* of marine origin from 2014 to 2018 (Xu K. et al., 2020). In 2021, Orfali et al. highlight secondary metabolites from various marine-derived *Aspergillus* species reported between 2015 and 2020 along with their biological potential and structural aspects whenever applicable (Orfali et al., 2021). In 2023, Li et al. summarized the antimicrobial compounds from marine *Aspergillus* from January 2021 to March 2023 (Li H. H. et al., 2023). However, no studies have been carried out on the antimicrobial compounds from marine *Aspergillus* from 2010 to 2024. It is believed that the study of *Aspergillus* living in marine environments will facilitate the discovery of drug lead compounds. Consequently, this review discussed the antibacterial substances derived from *Aspergillus* species in the marine environment from January 2010 to June 2024. A total of 117 cited references were presented in the review. It comprehensively covered the chemical diversity and antimicrobial properties of 337 reported compounds, including 145 new compounds isolated from marine-derived *Aspergillus* fungi. These compounds were structurally categorized into terpenoids (32 compounds), nitrogen-containing compounds (98 compounds), polyketides (139 compounds), steroids (18 compounds), and other compounds (50 compounds). Some potential compounds’ relevant biological and pharmacological activities are also highlighted, which will benefit future drug development and innovation. Notably, some antimicrobial compounds against human pathogenic bacteria produced by *Aspergillus* fungi also showed activities against agriculture and fish pathogenic bacteria and so on (Zhang et al., 2024; Xue et al., 2024), which might be suggested as one of the probable candidate drugs for “One Health” in the utilization in healthcare, agriculture, and fishery.

## 2 Structural and antibacterial activity studies

### 2.1 Terpenoids

Terpenoids were generally composed of structural units derived from isoprene or isopentane. A total of 32 antibacterial terpenoids (including 13 new compounds) were found in the marine-derived fungal genus *Aspergillus* sp., comprising 18 sesquiterpenes, four diterpenes, and 10 triterpenoids. The structures and the absolute configurations of the new compounds and novel skeleton compounds were elucidated by a detailed spectroscopic analysis of nuclear magnetic resonance (NMR) spectroscopy and mass spectrometry (MS) data, electronic circular dichroism (ECD) calculations, and single-crystal X-ray diffraction.

#### 2.1.1 Sesquiterpenes

**Figure 1:**
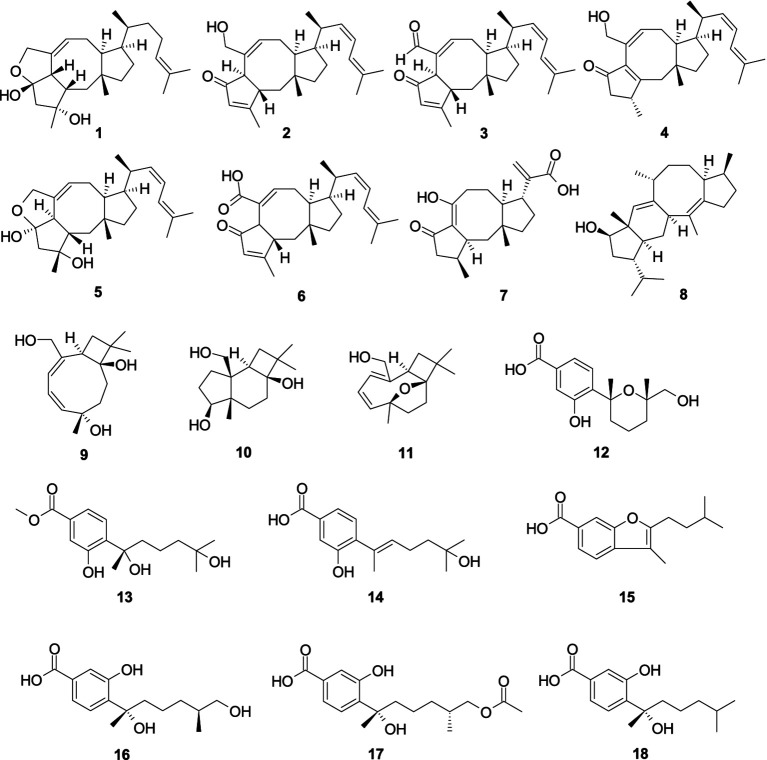
Chemical structures of antibacterial sesquiterpenes **1**–**18** from *Aspergillus* spp.

One new ophiobolin sesterterpenoid, (5*S*,6*S*)-16,17-dihydroophiobolin H (**1**), together with two known analogs, (6*α*)-21,21-*O*-dihydroophiobolin G (**2**) and 6-epi-ophiobolin G (**3**), were isolated from the cold-seep-derived fungus *A. insuetus* SD-512 (Chi et al., 2020). Compound **1**–**3** exhibited broad-spectrum antibacterial efficacy against eight tested bacterial strains (*Escherichia coli*, *P. aeruginosa*, *Aeromonas hydrophilia*, *Edwardsiella tarda*, *Vibrio alginolyticus*, *Vibrio anguillarum*, *Vibrio Parahemolyticus*, and *Vibrio vulnificus*) with the minimum inhibitory concentration (MIC) values from 4.0 to 32.0 μg/mL. A novel ophiobolin sesterterpenoid ophiobolin U (**4**) and a known analog (5*ɑ*,6*ɑ*)-ophiobolin H (**5**) were obtained from alga-derived fungus *A. ustus cf*-42 (Liu et al., 2013). Compounds **4**–**5** showed inhibitory effects against *E. coli*, demonstrating inhibition zones of 15.0 and 10.0 mm at a concentration of 30 μg/disk, respectively. Asperophiobolin E (**6**) was obtained from the coral-derived fungus *A. hiratsukae* SCSIO 5Bn_1_003 (Zeng et al., 2022a). Compound **6** demonstrated strong antibacterial efficacy against *Bacillus subtilis* (MIC, 17.0 μg/mL), which exhibited weak activity against *S. aureus*, with the MIC value of 102.86 μg/mL. One new sesterterpenoid, asperbrunneo acid (**7**), was obtained from the marine-derived fungus *Aspergillus brunneoviolaceus* MF180246 (Xu et al., 2024). Compound **7** showed weak antibacterial efficacy against *S. aureus* with the MIC value of 200 μg/mL. Aspergilol C (**8**) was obtained from the marine-derived fungus *Aspergillus* sp. ZZ1861 (Ha et al., 2024). Compound **8** exhibited potent antibacterial activity against *E. coli*, with the MIC value of 6.25 μg/mL. Punctaporonins B (**9**), D (**10**), and G (**11**), were obtained from the fungus *A. terreus* SCSIO 41202 (Zhang et al., 2024). Compounds **9**–**11** showed a strong antibacterial effect against *Xanthomonas citri* subsp. *citri* with the MIC values of 0.625, 0.625, and 0.3125 mg/mL, respectively. One novel bisabolene-type sesquiterpenoid, 12-hydroxysydowic acid (**12**), along with two known analogs, aspergoterpenin C (**13**) and engyodontiumone I (**14**), were extracted from the fungus *A. versicolor* SD-330 (Li et al., 2021). Compounds **12**–**14** exhibited selective inhibitory activity against *A. hydrophilia*, *E. coli*, *E. tarda*, and *Vibrio harveyi*, with the MIC values ranging 1.0–8.0 μg/mL. Aspergillusene B (**15**), (7*S*,11*S*)-(+)-12-hydroxysydonic acid (**16**), expansol G (**17**), and (*S*)-sydonic acid (**18**), were isolated from the fungus *Aspergillus. sydowii* LW09 (Yang et al., 2023). Compounds **15**, **17**, and **18** demonstrated weak antibacterial efficacy against *Ralstonia solanacarum* (the same MIC, 32.0 μg/mL). Compound **16** demonstrated weak antibacterial activity against *P. syringae*, exhibiting the MIC value of 32.0 μg/mL (Figure 1).

#### 2.1.2 Diterpenoids

**Figure 2:**
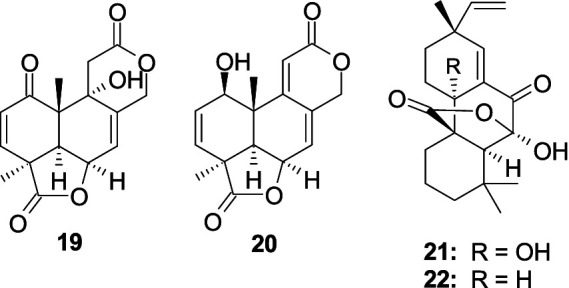
Chemical structures of antibacterial diterpenoids **19**–**22** from *Aspergillus* spp.

A new tetranorlabdane diterpenoid asperolide D (**19**), along with one known analog asperolide A (**20**), was isolated from the fungus *Aspergillus wentii* SD-310 (Li et al., 2016). Compounds **19** and **20** exhibited antibacterial activity against *E. tarda*, with the same MIC value of 16.0 μg/mL. Two pimarane diterpenes, sphaeropsidin A (**21**) and aspergiloid E (**22**), were obtained from the algal-derived fungus *Aspergillus porosus* G23 (Neuhaus et al., 2019). Compounds **21** and **22** showed activity against *S. aureus* ATCC 25923 and ATCC BAA-41, with the MIC values ranging 32.6–77.8 μM (Figure 2).

#### 2.1.3 Meroterpenoids

**Figure 3:**
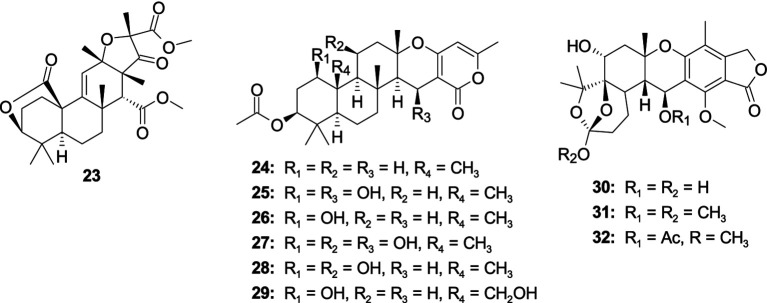
Chemical structures of antibacterial meroterpenoids **23**–**32** from *Aspergillus* spp.

A new 3,5-dimethylor-sellinic acid-based meroterpenoid, aspergillactone (**23**), from the marine-derived fungus *Aspergillus* sp. CSYZ-1 (Cen et al., 2021), exhibited potent antimicrobial activity against *Helicobacter pylori* (ATCC 43504, G27, Hp159, and BY583) and *S. aureus* (ATCC 25923, USA300, BKS231, BKS233) with the MIC values of 1.0–4.0 and 2.0–16.0 μg/mL. A new meroterpenoid, chevalone B (**24**), was obtained from the marine-derived fungus *Aspergillus* sp. H30 (Hu et al., 2019). Compound **24** showed weak antimicrobial activity against *S. aureus* with the MIC value of 50 μg/mL. Five new *α-*pyrone meroterpenoids, chevalones H–L (**25**–**29**), isolated from the gorgonian-derived fungus *A. hiratsukae* SCSIO 7S2001 (Chen X. Y. et al., 2022), showed antibacterial activities against *Micrococcus lutea*, *K. pneumoniae*, methicillin-resistant *Staphylococcus aureus* (MRSA) and *Streptococcus faecalis*, with the MIC values of 6.25–100 μg/mL. A new meroterpenoid, austalide R (**30**), and two known compounds, austalides M (**31**) and N (**32**), were isolated from the sponge-derived fungus *Aspergillus* sp. (Zhou et al., 2014). Compounds **30** and **31** displayed broad-spectrum inhibitory activity against eight tested strains (*Halomonas aquamarine*, *Pseudoalteromonas elyakovii*, *V. harveyi*, *Roseobacter litoralis*, *Polaribacter irgensii*, and *Shewanella putrefaciens*) with the MIC values range from 0.01 to 0.1 μg/mL, whereas **32** displayed inhibitory activity against *V. natriegens* and *R. litoralis* with the same MIC value of 0.01 μg/mL (Figure 3).

### 2.2 Nitrogen-containing compounds

Nitrogenous secondary metabolites were ubiquitous in nature with a wide range of biological activities. A total of 98 nitrogen-containing antimicrobial compounds (including 53 new compounds) were discovered from the genus *Aspergillus* sp., including 39 indole alkaloids, 11 quinazolinone alkaloids, four cytochalasan alkaloids, 13 peptides, and 31 other nitrogen-containing metabolites. The structures and the absolute configurations of the new compounds and novel skeleton compounds were elucidated by a detailed spectroscopic analysis of NMR and MS data, ECD calculations, and single-crystal X-ray diffraction. The absolute configurations of the amino acid residues of the peptides were determined by Marfey’s method.

#### 2.2.1 Indole alkaloids

**Figure 4:**
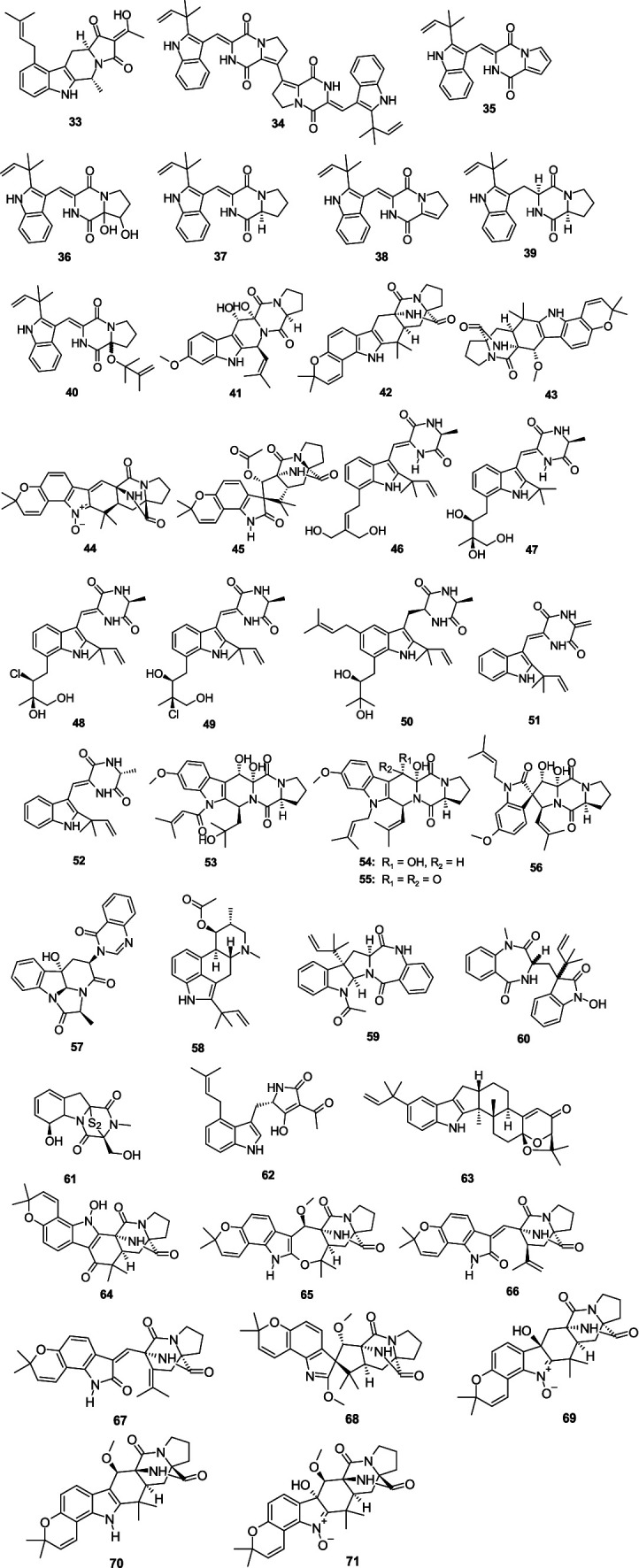
Chemical structures of antibacterial indole alkaloids **33**–**71** from *Aspergillus* spp.

Griseofamine A (**33**), isolated from the deep-sea derived fungus *Aspergillus* sp. SCSIO 41024 (Chen et al., 2020), exhibited weak antibacterial activity against *E. coli* with the MIC value of 64.0 μg/mL. Four new indole alkaloids brevianamides S–V (**34**–**37**), together with two known analogs brevianamide K (**38**) and deoxybrevianamide E (**39**), were isolated from the fungus *A. versicolor* MF030 (Song F. H. et al., 2021). Compounds **34**–**39** displayed antibacterial effects against *Bacille Calmette-Guérin* (BCG), with the MIC values of 6.25, 50, 25, 100, 50, and 100 μg/mL, respectively. Compound **39** also showed antibacterial effects against *S. aureus* and *B. subtilis* with the MIC values of 100 and 50 μg/mL, respectively. A new alkaloid, 9ξ-*O*-2(2,3-dimethylbut-3-enyl)brevianamide Q (**40**), was isolated from the alga-derived fungus *A versicolor* pt20 (Miao et al., 2012). Compound **40** exhibited a weak inhibitory effect on *E. coli* and *S. aureus*, with the same inhibition zone of 7.0 mm at a disk concentration of 30 μg/mL, respectively. 12,13-Dihydroxy-fumitremorgin C (**41**), separated from the fungus *Aspergillus* sp. SCSIO Ind09F01 demonstrated potent inhibitory activity against *Mycobacterium tuberculosis*, with the MIC value of 2.41 μM (Luo et al., 2017). (−)-stephacidin A (**42**) was separated from a gorgonian-derived fungus *Aspergillus* sp. XS-20090066 revealed a selective antibacterial effect against *Staphylococcus epidermidis* (MIC, 14.5 μM) (Chen et al., 2013). Notoamide *F* (**43**) was obtained from the fungus *A. sclerotiorum* GDST-2013-0501 (Wang C. Y. et al., 2022). Compound **43** exhibited a moderate antibacterial effect against *S. epidermidis*, with the MIC value of 12.5 μM. Two new indole alkaloids, asperthrins A (**44**) and E (**45**), were obtained from the fungus *Aspergillus* sp. YJ191021 (Yang et al., 2021). Compound **44** displayed antibacterial effects against *E. tarda*, *V. anguillarum*, *A. hydrophilia* and *Vibrio parahaemolyticus* (MIC, 16, 8, 32, and 16 μg/mL, respectively). Compound **45** displayed an inhibitory effect against *Rhizoctonia solani* with the MIC value of 25 μg/mL. Five new indole alkaloids, 24,25-dihydroxyvariecolorin G (**46**), 25-hydroxy-rubrumazine B (**47**), 22-chloro-25-hydroxyrubrumazine B (**48**), 25-hydroxy-variecolorin *F* (**49**), and 27-epi-aspechinulin D (**50**), along with the known analog neoechinulin B (**51**) were isolated from the fungus *Aspergillus Chevalieri* CS-122 (Yan et al., 2023). Compound **46** displayed significant inhibitory activity against *E. coli* (MIC, 4.0 μg/mL), while compound **48** displayed an inhibitory effect against *Vibrio harveyi* (MIC, 8.0 μg/mL). Moreover, compounds **47** and **50** exhibited broad-spectrum antibacterial effects against five evaluated bacterial strains (*V. harveyi*, *E. tarda*, *Aeromonas hydrophila*, *E. coli*, and *Micrococcus luteus*) with the MIC values ranging 16.0–32.0 μg/mL. Compound **51** showed significant activities against *A. hydrophila* (MIC, 4.0 μg/mL) and *E. coli* (MIC, 8.0 μg/mL). A known compound, neoechinulin A (**52**), was separated from the coral-derived fungus *A. hiratsukae* SCSIO 7S2001 (Chen X. Y. et al., 2022). Compound **52** showed weak antibacterial activities against *K. pneumoniae* and *S. faecalis* with MIC values of 50.0 and 12.5 μg/mL, respectively. Compound **52** also had an antibacterial effect against *H. pylori* Hp159 with the MIC value of 16 μg/mL (Yu et al., 2022). Asperfumigatin (**53**), 12,13-dihydroxyfumitremorgin C (**41**), fumitremorgin B (**54**), 13-oxofumitremorgin B (**55**), spirotryprostatin C (**56**), (−)-chaetominine (**57**), and fumigaclavine C (**58**) were isolated from the fungus *Aspergillus fumigatus* H22 (Zhang R. et al., 2022). Compounds **41** and **53**–**58** showed antibacterial activity against MRSA, with the MIC values from 1.25 to 25.0 μM. Epi-aszonalenin A (**59**) were isolated from the fungus *A. fumigatus* SCSIO 41012 (Limbadri et al., 2018). Compound **59** displayed antibacterial effect against *A. baumanii* ATCC19606 (MIC, 50 μg/mL) and ATCC 15122 (MIC, 6.25 μg/mL). A new tryptophan-derived alkaloid, 3-((1-hydroxy-3-(2-methylbut-3-en-2-yl)-2-oxoindolin-3-yl)methyl)-1-methyl-3,4-dihydrobenzo[e]-[1,4]-diazepine-2,5-dione (**60**), was separated from the sponge-associated fungus *Aspergillus* sp. (Zhou et al., 2014). Compound **60** selectively inhibited *V. harveyi* and *Vibrio natriegens*, with the same MIC value of 1.0 μg/mL. Gliotoxin (**61**), separated from the fungus *Aspergillus* sp. SCSIO Ind09F01, strongly inhibited *M. tuberculosis* (MIC, 0.03 μM) (Luo et al., 2017). *β*-Cyclopiazonic acid (**62**), isolated from sponge-derived fungus *Aspergillus felis* FM324, showed antibacterial effects on *S. aureus*, MRSA, and *B. subtilis*—all exhibiting the same MIC value of 59.2 μM (Wang et al., 2021). One new indole-diterpenoid, (2*R*,4b*R*,6a*S*,12b*S*,12c*S*,14a*S*)-4b-deoxy-*β*-aflatrem (**63**), was isolated from the marine-derived fungus *Aspergillus flavus* OUCMDZ-2205 (Sun et al., 2014). Compound **63** exhibited antibacterial activity against *S. aureus* with the MIC value of 20.5 μM. Eight new notoamide-type alkaloids, sclerotiamides K–R (**64**–**71**), were isolated from a marine gorgonian-derived fungus *A. sclerotiorum* LZDX-33-4 (Meng et al., 2022). Compounds **64**–**71** showed antibacterial activity against *S. aureus* ATCC29213 with MIC values ranging 4–64 μM (Figure 4).

#### 2.2.2 Quinazolinone alkaloids

**Figure 5:**
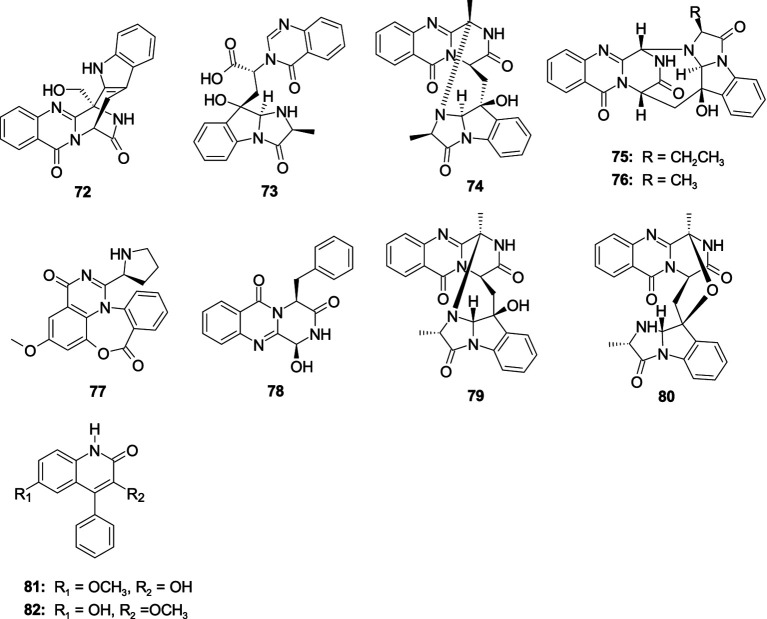
Chemical structures of antibacterial quinazolinone alkaloids **72**–**82** from *Aspergillus* spp.

Two novel alkaloids fumigatosides E–F (**72**–**73**), along with a known alkaloid fumiquinazoline G (**74**), were isolated from *A. fumigatus* SCSIO 41012 (Limbadri et al., 2018). Compound **72** showed activities against *Acinetobacter baumanii* ATCC 19606, *A. baumanii* ATCC 15122, *S. aureus* ATCC 16339, and *K. pneumonia* ATCC 14578 with the MIC values of 12.5, 6.25, 6.25, and 12.5 μg/mL, respectively. Compound **73** exhibited activity against *A. baumanii* ATCC 19606 with the MIC value of 6.25 *μ*g/mL. Compound **73** exhibited significant activity against *S. aureus* ATCC16339 and 29,213, (MIC, 1.56 and 0.78 μg/mL). Compound **74** showed activities against *A. baumanii* ATCC 15122, *S. aureus* ATCC 16339, *S. aureus* ATCC29213, and *K. pneumonia* ATCC 14578 with the MIC values of 6.25, 12.5, 12.5, and 25 μg/mL, respectively. One new alkaloid cottoquinazoline H (**75**) and a known analog cottoquinazoline A (**76**) were separated from the coral-associated fungus *A. versicolor* AS-212 (Dong et al., 2023a). Compound **75** showed potent inhibitory effects against the aquatic pathogenic bacterium *Vibrio harvryi* (MIC, 18.1 μM) and *V. parahemolyticus* (MIC, 9.0 μM). Compound **76** exhibited moderate activity against *A. hydrophila* with an MIC value of 18.6 μM. Compound **76** also showed strong antibacterial effect against *E. coli* with the MIC value of 5.0 μM (Zhang L. et al., 2020; Zhang Y. H. et al., 2020). A new alkaloid, aspergicin (**77**), was separated from the mixed cultivation of two mangrove-associated mangrove fungi *Aspergillus* sp. (Zhu et al., 2011). Compound **77** exhibited a moderate antibacterial effect against *B. subtilis* and *B. dysenteriae*, with consistent MIC values of 15.6 μg/mL. Brevianamide M (**70**) was separated from the alga-associated fungus *A. versicolor* pt20 (Miao et al., 2012). Compound **78** exhibited antibacterial activity against *E. coli* and *S. aureus*, with inhibition zones of 11.0 and 10.0 mm observed at a concentration of 30 μg/disk, respectively. Fumiquinazolines D (**79**) and C (**80**), were separated from the sea cucumber-associated fungus *A. fumigatus* M580 (Tuan et al., 2022). Compounds **79** and **80** exhibited antibacterial activity against Gram-positive *Enterococcus faecalis* with the same MIC value of 32.0 μg/mL. 3-Hydroxy-6-methoxy-4-phenylquinolin-2(1*H*)-one (**81**) and 3-methoxy-6-hydroxy-4-phenylquinolin-2(1*H*)-one (**82**) were separated from a coral-derived fungus *A. versicolor* AS-212 (Dong et al., 2023b). Compounds **81** and **82** demonstrated an antibacterial effect against aquatic pathogenic bacteria *V. harveyi* and *V. alginolyticus*, with the MIC values from 8 to 32 μg/mL (Figure 5).

#### 2.2.3 Cytochalasan alkaloids

**Figure 6:**
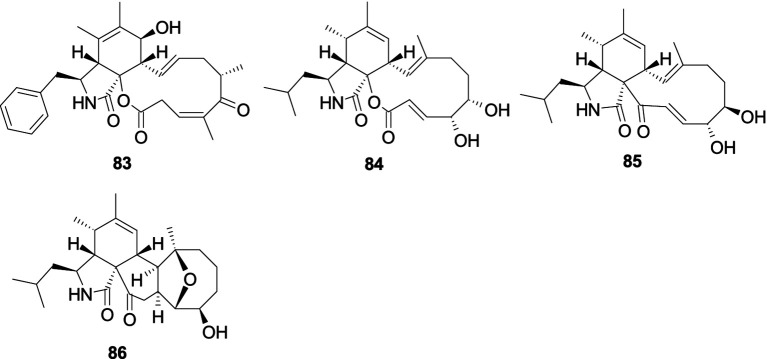
Chemical structures of antibacterial cytochalasan alkaloids **83**–**86** from *Aspergillus* spp.

Cytochalasin Z17 (**83**) was isolated from the sponge-derived fungus *Aspergillus* sp., and it showed selective and pronounced activity effect *R. litoralis* with the MIC value of 0.0001 μg/mL (Zhou et al., 2014). Aspochalasins I (**84**), D (**85**), and PZ (**86**), were separated from the coral-associated fungus *Aspergillus elegans* (Zheng et al., 2013). Compound **84** showed moderate antibacterial activity against *S. epidermidis* (MIC, 20 μM) and *S. aureus* (MIC, 10 μM). Compound **85** exhibited extensive antibacterial effects against four pathogenic bacteria (*S. albus*, *S. aureus*, *E. coli*, and *Bacillus cereus*) with a consistent MIC value of 10 μM. Compound **86** displayed an antibacterial effect against *S. epidermidis* with the same MIC value of 20 μM (Figure 6).

#### 2.2.4 Peptides

**Figure 7:**
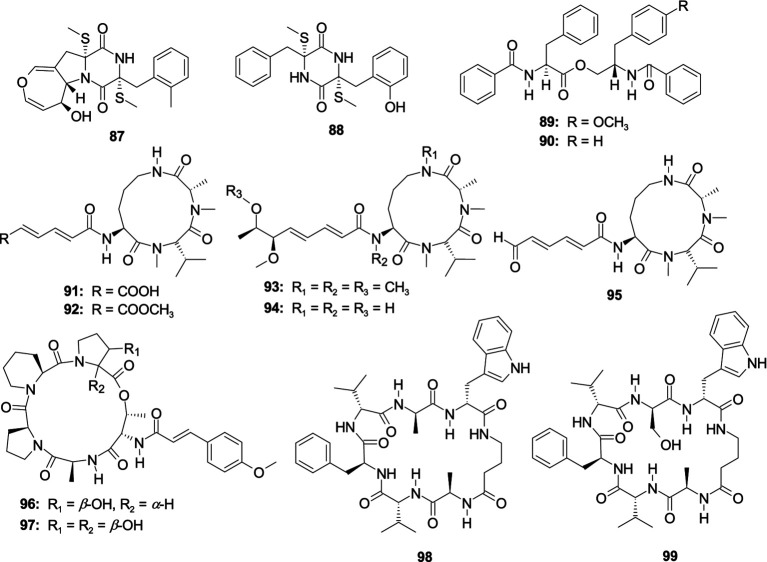
Chemical structures of antibacterial cytochalasan alkaloids **87**–**99** from *Aspergillus* spp.

One novel thiodiketopiperazine, emestrin M (**87**), and a known monomer compound, emethacin C (**88**), were separated from the fungus *A. terreus* RA2905 (Wu et al., 2020a). Compounds **87** and **88** displayed antibacterial activity against *P. aeruginosa* ATCC 27853 with the MIC values of 64 and 32 μg/mL, respectively. One novel phenylalanine derivative 4′-OMe-asperphenamate (**89**) and another known phenylalanine derivative asperphenamate (**90**) were separated from the coral-associated fungus *A. elegans* ZJ-2008010 (Zheng et al., 2013). Compounds **89** and **90** showed an antibacterial effect against *S. epidermidis* with the same MIC value of 10.0 μM. Three novel aspochracin-type cyclic tripeptides, sclerotiotides M–O (**91**–**93**), together with two previously identified analogs, sclerotiotides L (**94**) and *F* (**95**), were originated from the fungus *Aspergillu insulicola* HDN151418 (Sun et al., 2020). Compounds **91** and **92** dispalyed a broad antibacterial effect on eight pathogenic strains (*B. cereus*, *Proteus*species, *Mycobacterium phlei*, *B. subtilis*, *V. parahemolyticus*, *E. tarda*, MRCNS, and MRSA) with the MIC values ranging 1.56–25.0 μM. Compound **93** showed an antibacterial effect on *E. tarda* and *V. parahemolyticus* with consistent MIC values of 25 μM. Compounds **94** and **95** showed antibacterial activity effects on four bacterial strains (*B. cereus*, *Proteus* species, *E. tarda*, and *V. parahemolyticus*) with consistent MIC values of 25 μM. Two new pentadepsipeptides, aspertides D (**96**) and E (**97**), were originated from the multistrain fermentation of two marine-associated fungi *Aspergillus tamarii* MA-21 and *Aspergillus insuetus* SD-512 (Chi et al., 2023). Compound **96** exhibited an antibacterial effect on four aquatic bacterial pathogens (*E. tarda*, *V. alginolyticus*, *V. anguillarum*, and *V. vulnificus*) with the MIC values of 8.0–32.0 μg/mL. Compound **97** had an antibacterial effect on *E. tarda* and *S. aureus* with the MIC values of 16.0 and 8.0 μg/mL, respectively (Figure 7). Unguisins A (**98**) and B (**99**) were isolated from marine sponge-derived fungus *Aspergillus nidulans* M256, displayed antibacterial activity against *E. faecalis* with the MIC values of 32 and 128, respectively.

#### 2.2.5 Other nitrogen-containing metabolites

**Figure 8:**
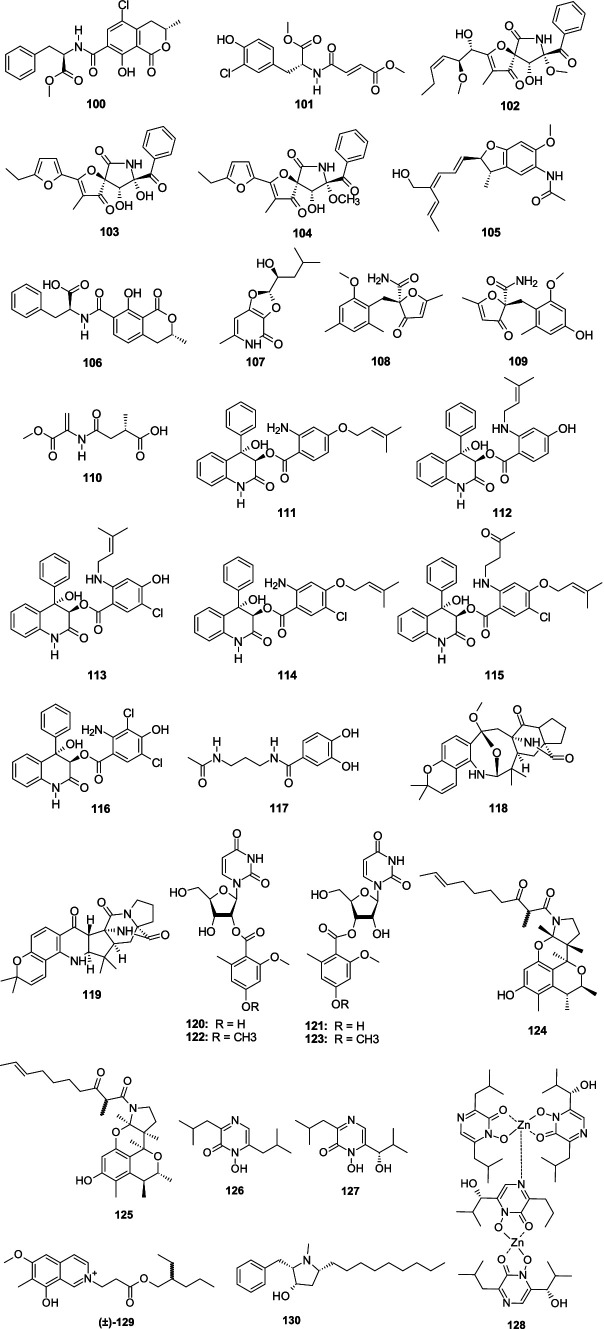
Chemical structures of other nitrogen-containing antibacterial metabolites **100**–**130** from *Aspergillus* spp.

Ochratoxin A methyl ester (**100**) was separated from the fungus *A. elegans* KUFA0015 (Kumla et al., 2021). Compound **100** showed a broad spectrum of antibacterial effect against *E. faecalis* ATCC29212, *E. faecalis* B3/101, *S. aureus* ATCC29213, and MRSA *S. aureus* 66/1 with the MIC values of 16, 16, 8, and 16 μg/mL, respectively. A new chlorinated amino acid derivative, aspergamide A (**101**), was obtained from the sponge-associated fungus *Aspergillus* sp. LS53 (Zhang L. et al., 2020; Zhang Y. H. et al., 2020). Compound **101** had a weak antibacterial effect on *V. harveyi*, with the MIC value of 16 μg/mL. 11-*O*-methylpseurotin A (**102**), azaspirofurans B (**103**), and A (**104**) were separated from the marine-associated fungus *A. fumigatus* H22 (Zhang R. et al., 2022). Compounds **102**–**104** showed a strong antibacterial effect against MRSA (MIC, 10.0, 5.0, and 5.0 μM, respectively). A new benzofuran derivative, dibetanide (**105**), was separated from the sponge-derived fungus *Aspergillus* sp. LS57 (Li W. H. et al., 2023). Compound **105** displayed inhibitory activity against *Botrytis cinerea* with the MIC value of 256 μg/mL. Ochratoxin B (**106**) was separated from the sponge-associated fungus *A. elegans* KUFA0015 (Duraes et al., 2021). Compound **106** had a weak antibacterial effect against *S. aureus* 272,123 with the MIC value of 50.0 μM. Dihydroisoflavipucine (**107**) was separated from the sponge-associated fungus *Aspergillus* sp. and showed strong activity against *R. litoralis* with the MIC value of 0.0001 μg/mL (Zhou et al., 2014). A racemate of benzyl furanone, (+)-asperfuranone (**108**) and (−)-asperfuranone (**109**), were separated from coral-associated fungus *A. terreus* RA2905 (Wu et al., 2020b). Compounds **108**–**109** displayed an antibacterial effect against *P. aeruginosa* ATCC 27853 with the MIC values of 32 and 128 μg/mL, respectively. A novel compound, carneusin B (**110**), was separated from the fungus *Aspergillus carneus* GXIMD00519 (Lu et al., 2023). Compound **110** displayed weak antibacterial activities against *Vibrio rotiferianus* and *Alteromonas macleodii* with the consistent MIC value of 64.0 μg/mL. Seven novel benzoic acid-containing alkaloids, asperalins A–F (**111**–**116**) and *N*-(3-acetamidopropyl)-3,4-dihydroxybenzamide (**117**), were separated from a seagrass-associated fungus *Aspergillus alabamensis* SYSU-6778 (Hu et al., 2023). Compounds **111**–**116** revealed moderate-to-potent activities against *Streptococcu iniae* and *Streptococcus parauberis* with the MIC values ranging 2.2–87.3 μM, respectively. Compound **117** showed weak antibacterial effect on *Edwardsiella ictaluri* with MIC value of 79.3 μM. Two new compounds, sclerotiamides I (**118**) and J (**119**), were isolated from a marine gorgonian-derived fungus *A. sclerotiorum* LZDX-33-4 (Meng et al., 2022). Compounds **118** and **119** displayed antibacterial activity against *S. aureus* ATCC29213 with the same MIC value of 16 μM. Two novel nucleoside derivatives, kipukasins H (**120**) and I (**121**), together with two known analogs, kipukasins E (**122**) and D (**123**), originated from the fungus *A. versicolor* (Chen et al., 2014). Compounds **120**–**123** exhibited antibacterial effects on *S. epidermidis* with the MIC values of 12.5, 12.5, 50.0, and 50.0 μM, respectively. Two rare tetracyclic skeleton alkaloids, perinadines B (**124**) and C (**125**), were originated from the fungus *Aspergillus* sp. LS116 (Liu Y. et al., 2022). Compounds **124**–**125** exhibited moderate antibacterial effects on *B. subtilis* (MIC, 32.0 and 64.0 μg/mL, respectively). Neoaspergillic (**126**), isolated from coral-associated fungus *Aspergillus* sp. CF07002 showed a weak antibacterial effect on three tested bacterial strains (*B. cereus*, *K. pneumoniae*, and *E. coli*) with MIC values ranging 30.0–40.0 μg/mL (Cardoso-Martinez et al., 2015). A novel dimer of a zinc complex, dizinchydroxyneoaspergillin (**128**), and a known compound hydroxyneoaspergillic acid (**127**), originated from the fungus *Aspergillus ochraceopetaliformis* SCSIO 41018 (Guo et al., 2021). Compound **127** exhibited potent inhibitory effects against *A. baumannii* with the MIC value of 0.45 μg/mL. Compound **128** showed significant bactericide effects against MRSA, *S. aureus*, *E. faecalis*, *A. baumannii*, and *K. pneumonia* with the MIC values from 0.45 to 7.8 μg/mL. A racemic mixture alkaloid, (±)-puniceusine N (**129**), was isolated from the fungus *Aspergillus puniceus* SCSIO z021 (Liu C. M. et al., 2022). Compound (±)-**129** had medium antibacterial activities against *S. aureus*, MRSA, and *E. coli* with a consistent MIC value of 100 μg/mL. Preussin (**130**), separated from the fungus *Aspergillus candidus* KUFA0062, displayed inhibitory activity against *S. aureus* ATCC 29213, *E. faecalis* ATCC 29212, MRSA, and vancomycin-resistant *enterococci* with consistent MIC value of 32.0 μg/mL (Buttachon et al., 2018) (Figure 8).

### 2.3 Polyketides

Polyketides were a group of compounds recognized for their wide range of structures and biological activities. These compounds were produced through a series of Claisen condensation reactions, usually utilizing acetyl-coenzyme A (acetyl-CoA), malonyl-coenzyme A (malonyl-CoA), and other substrates. A total of 139 antibacterial polyketides (including 54 new compounds) were separated from the genus of *Aspergillus* sp., including 20 anthraquinones, 31 xanthones, 59 lactones, and 29 other polyketide metabolites. The structures and the absolute configurations of the new compounds were elucidated by a detailed spectroscopic analysis of NMR and MS data, ECD calculations, as well as single-crystal X-ray diffraction.

#### 2.3.1 Anthraquinones

**Figure 9:**
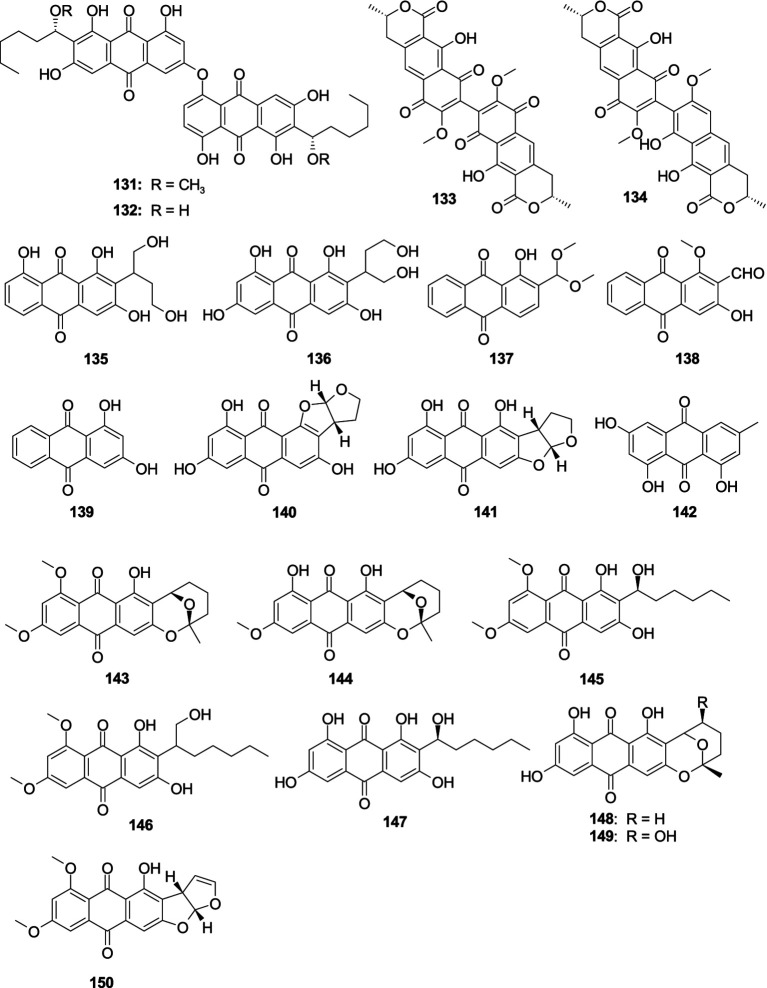
Chemical structures of antibacterial anthraquinones **131**–**150** from *Aspergillus* spp.

Two new anthraquinone dimers, 6,6′-oxybis(1,3,8-trihydroxy-2-((*S*)-1-methoxyhexyl)anthracene-9,10-dione) (**131**) and 6,6′-oxybis(1,3,8-trihydroxy-2-((*S*)-1-hydroxyhexyl)anthracene-9,10-dione) (**132**) were originated from the fungus *A. versicolor* INF16-17 (Li et al., 2019). Compounds **131**–**132** demonstrated a selective antibacterial effect on *S. aureus* at a concentration of 30.0 μg/well. Xanthomegnin (**133**) and viomellein (**134**) were separated from the sponge-associated fungus *A. elegans* KUFA0015 (Kumla et al., 2021). Compounds **133**–**134** had a moderate antibacterial effect on *E. faecalis* ATCC29212, *S. aureus* ATCC29213, and *S. aureus* 66/1 (MRSA), with the MIC values ranging 2.0–32.0 μg/mL. One new anthraquinone versiconol B (**135**) and a known compound versiconol (**136**) were originated from the fungus *Aspergillus* sp. F40 (Tian et al., 2018). Compounds **135**–**136** exhibited weak antibacterial activity against *S. aureus* and *V. parahaemolyticus* with the MIC values of 12–48 μg/mL. One novel anthraquinone derivative, 2-(dimethoxymethyl)-1-hydroxyanthracene-9,10-dione (**137**), along with two previously reported analogs, damnacanthal (**138**) and xanthopurpurin (**139**), were separated from the fungus *A. versicolor* 3A00029 (Wang et al., 2018). Compound **137** displayed a potent inhibitory effect on MRSA (ATCC 43300 and CGMCC 1.12409), with the MIC values of 3.9 and 7.8 μg/mL, respectively. Compound **138**–**139** showed a weak antibacterial effect on *V. vulnificus* MCCC E1758, *V. rotiferianus* MCCC E385, and *Vibrio campbellii* MCCC E333, with the MIC values ranging 62.5–125 μg/mL. One novel anthraquinone isoversicolorin C (**140**) and one known anthraquinone derivative versicolorin C (**141**) were separated from the fungus *A. nidulans* MA-143 (Yang et al., 2018a). Compound **140** demonstrated a remarkable antibacterial effect on *V. alginolyticus* (MIC, 1.0 μg/mL) and *E. ictaluri* (MIC, 4.0 μg/mL). Compound **141** exhibited an antibacterial effect against five tested bacterial strains (*E. coli*, *M. luteus*, *V. alginolyticus*, *V. parahaemolyticus*, and *E. ictaluri*), with the MIC values ranging 1.0–8.0 μg/mL. Emodin (**142**) was separated from the fungus *A. fumigatus* MF029 (Song Z. J. et al., 2021). Compound **142** showed potent activity against BCG with the MIC value of 1.25 μg/mL, along with **142** demonstrated moderate antibacterial activities effect on MRSA and *S. aureus* with the same MIC value of 50.0 μg/mL. 6,8-Di-*O*-methylaverufin (**143**) and 6-*O*-methylaverufin (**144**) were separated from the alga-associated fungus *A. versicolor* pt20 (Miao et al., 2012). Compounds **143**–**144** displayed an antibacterial effect against *E. coli* and *S. aureus*, showing the same inhibition zone of 10.0 mm at 30 μg/disk. The new anthraquinone, 6,8-di-*O*-methylaverantin (**145**), together with one known congener 6,8-di-*O*-methylversiconol (**146**), was separated from the fungus *A. versicolor* EN-7 (Zhang et al., 2012). Compounds **145** and **146** showed weak inhibition against *E. coli*, with the inhibition zones 7.0 and 6.5 mm at 20 μg/disk, respectively. Averantin (**147**), averufin (**148**), and nidurufin (**149**) were originated from the fungus *A. versicolor* PF10M (Lee et al., 2010). Compounds **147**–**149** showed a better antibacterial effect on *Streptococcus pyogenes* and *S. aureus* with the MIC values from 0.78 to 6.25 μg/mL. 6,8-Di-*O*-methylversicolorin A (**150**) was originated from the fungus *Aspergillus* sp. WHUF05236 (Lv et al., 2022). Compound **150** displayed an antibacterial effect against *H. pylori*, with the MIC values from 20.00 to 43.47 μM (Figure 9).

#### 2.3.2 Xanthones

**Figure 10:**
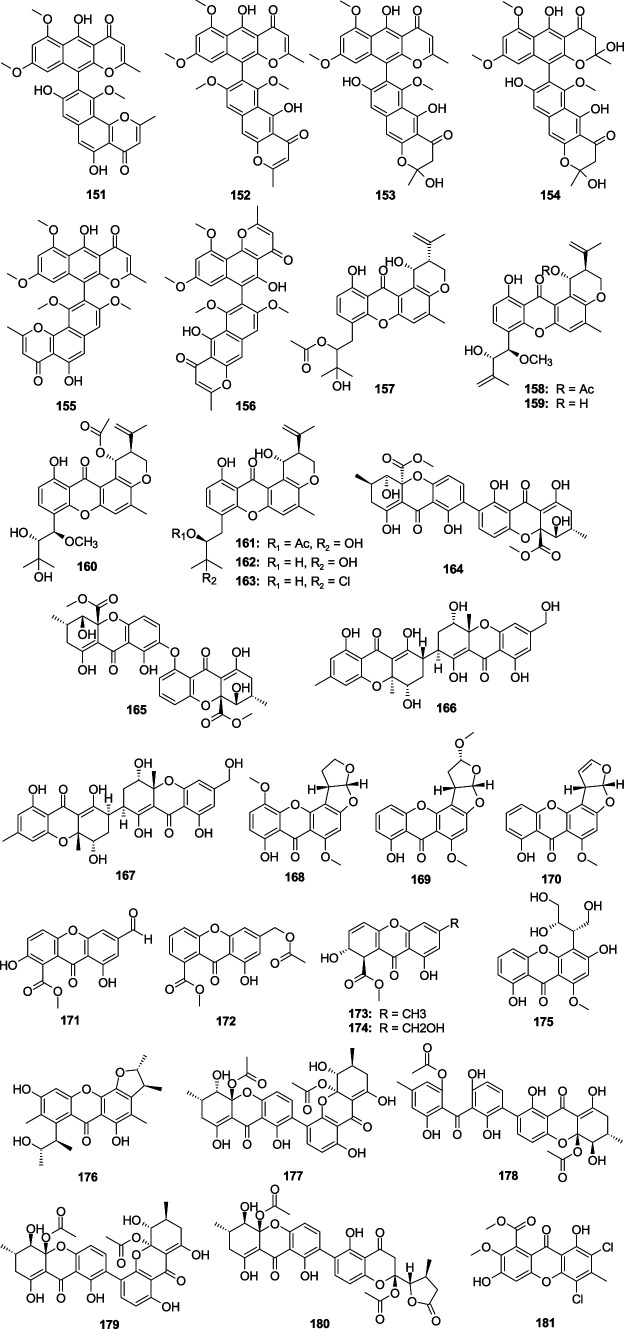
Chemical structures of antibacterial xanthones **151**–**181** from *Aspergillus* spp.

Asperpyrone A (**151**), aurasperones A (**152**), *F* (**153**), and B (**154**), were separated from the mangrove-associated fungus *Aspergillus* sp. DM94 (Gou et al., 2020). Compound **151**–**154** displayed an obvious antibacterial effect on *H. pylori* with the MIC values ranging 4.0–32.0 μg/mL. Fonsecinone A (**155**) and asperpyrone C (**156**) were separated from the fungus *A. welwitschiae* CUGBMF180262 (Han et al., 2022). Compounds **155** and **156** showed moderate antibacterial activities against *H. pylori* with the same MIC value of 16 μg/mL. Three novel prenylxanthone derivatives, aspergixanthones I–K (**157**–**159**), and four known analogss aspergixanthone A (**160**), 15-acetyl tajixanthone hydrate (**161**), tajixanthone hydrate (**162**), and 16-chlorotajixanthone (**163**), were originated from the fungus *Aspergillus* sp. ZA-01 (Zhu et al., 2018). Compounds **157**–**163** displayed anti-*Vibrio* activities to three pathogenic *Vibrio* spp. (*V*. *parahemolyticus*, *V. anguillarum*, and *V. alginolyticus*), with the MIC values between 1.56 and 25.0 μM. Among them, **157** exhibited significant anti-*Vibrio* activity, suggesting that the propenyl group at C-20 with *α*-stereoconfiguration might be crucial for the anti-*Vibrio* activity. Homodimeric tetrahydroxanthone secalonic acid D (**164**) was isolated from *A*. *aculeatinus* WHUF0198 and **164** performed activities against *H. pylori* G27, *H. pylori* 26,695, *H. pylori* 129, *H. pylori* 159, *S. aureus* USA300, and *B. subtilis* 168 with MIC values of 4.0, 4.0, 2.0, 2.0, 2.0, and 1.0 μg/mL, respectively (Wu et al., 2023). A new tetrahydroxanthone dimer, 5-epi-asperdichrome (**165**), was originated from the mangrove-associated fungus *A. versicolor* HDN1009 (Yu et al., 2018). Compound **165** exhibited weak activity against four tested bacterial strains (*V. parahemolyticus*, *B. subtilis*, *M. phlei*, and *P. aeruginosa*), with the MIC values ranging 100.0–200.0 μg/mL. Two new heterodimeric tetrahydroxanthones, aflaxanthones A (**166**) and B (**167**), were separated from mangrove-associated fungus *A. flavus* QQYZ (Zang et al., 2022). Compound **166** possessed a moderate inhibitory effect on MRSA (MIC, 12.5 μM), and compounds **166** and **167** showed a weak inhibitory effect on *B. subtilis* with the same MIC value of 25 μM. A new sterigmatocystin, 5-methoxydihydrosterigmatocystin (**168**), was originated from the sponge-associated fungus *A. versicolor* MF359 (Song et al., 2014). Compound **168** exhibited a significant antibacterial effect against *B. subtilis* (MIC, 3.125 μg/mL) and *S. aureus* (MIC, 12.5 μg/mL). Oxisterigmatocystin C (**169**) was separated from the fungus *Aspergillus* sp. F40 (Tian et al., 2018). Compound **169** displayed weak antibacterial activity against *S. aureus* (MIC, 48.0 μg/mL). Sterigmatocystin (**170**) originated from a sponge-derived fungus *A. sydowii* DC08 (Handayani et al., 2022). Compound **170** showed activities against MRSA, Multidrug-resistant *P. aeruginosa* (MDRPA), *E. coli*, *S. aureus*, and *P. aeruginosa* with the MIC values of 64.0, 128.0, 16.0, 32.0, and 32.0 μg/mL, respectively. Two new anthrone derivatives, 2-hydroxy-6-formyl-vertixanthone (**171**) and 12-*O*-acetyl-sydowinin A (**172**), together with two known analogs aspergillusone A (**173**) and AGI-B4 (**174**), were originated from the fungus *A. sydowii* C1-S01-A7 (Wang et al., 2019). Compounds **171**–**174** showed weak activities to MRSA with the MIC values ranging 15.0–32.0 μg/mL. A new xanthone, isosecosterigmatocystin (**175**) was separated from the fungus *A. nidulans* MA-143 (Yang et al., 2018a). Compound **175** showed weak activity against *E. ictaluri* (MIC, 16.0 μg/mL). A new citrinin dimer, *seco*-penicitrinol A (**176**), was separated from the algal-associated fungal *A. sydowii* EN-534 (Yang et al., 2018b). Compound **176** showed weak inhibitory activity against four bacterial strains (*M. luteus*, *E. ictaluri*, *V. alginolyticus*, and *V. c*), with the MIC values ranging 16.0–32.0 μg/mL. Secalonic acid F1 (**177**), secalonic acid H (**178**), penicillixanthone A (**179**), and chrysoxanthone C (**180**) showed weak antibacterial activity against *S. aureus* with the MIC values 25.0, 50.0, 6.25, and 50.0 μg/mL, respectively, which were separated from the fungus *A. brunneoviolaceus* MF180246 (Xu et al., 2024). A new chlorinated biphenyl, aspergetherin A (**181**), displayed weak activity against MRSA 05–72 and MRSA USA300, with the same MIC value of 128.0 μg/mL, which was separated from the sponge-associated fungus *A. terreus* 164,018 (Li J. X. et al., 2023) (Figure 10).

#### 2.3.3 Lactones

**Figure 11:**
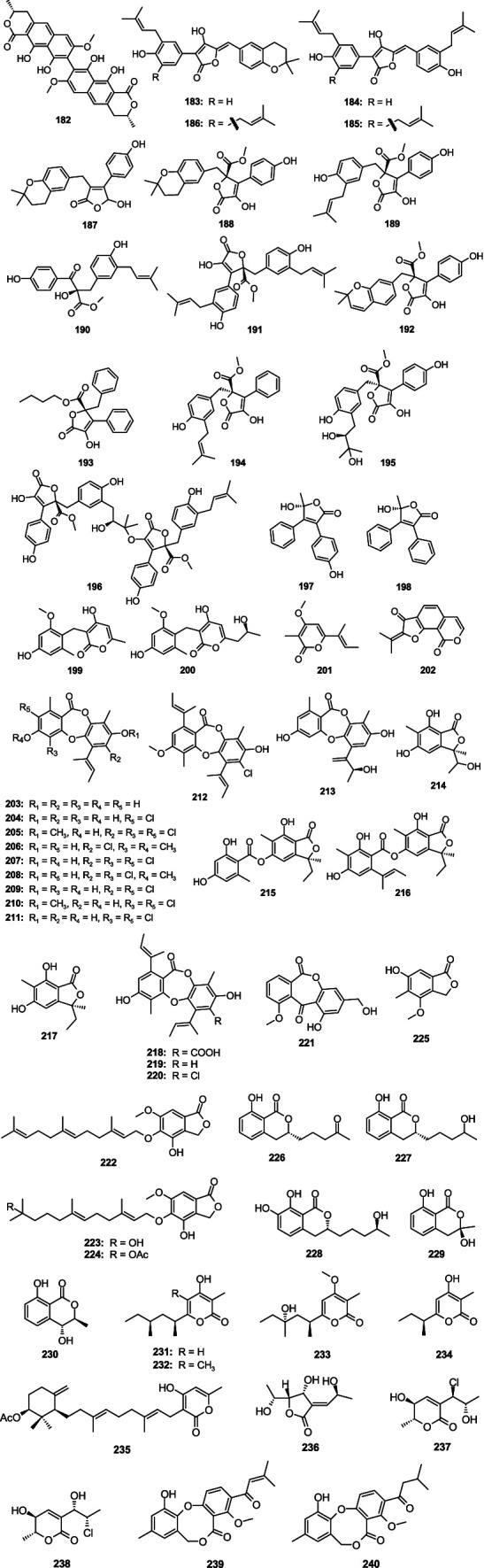
Chemical structures of antibacterial lactones **182**–**240** from *Aspergillus* spp.

Vioxanthin (**182**) showed significant antibacterial effect on *E. faecalis* ATCC29212, *E. faecalis* (VRE) B3/101, *S. aureus* ATCC29213, and *S. aureus* (MRSA) 66/1 with the MIC values 2.0, 1.0, 2.0 and 0.5, respectively, which was separated from the sponge-associated fungus *A. elegans* KUFA0015 (Kumla et al., 2021). Two new prenylated phenylbutyrolactones, aspulvinones R–S (**185**–**186**), together with two known compounds aspulvinones B′ (**183**) and H (**184**) were separated from the fungus *Aspergillus flavipes* KUFA1152 (Machado et al., 2021). Compounds **183**–**186** displayed strong activities against *E. faecalis* and *S. aureus* with the MIC values ranging 8.0–16.0 μg/mL. Asperteretal E (**187**) and aspernolide A (**188**) were originated from the fungus *A. terreus* SCSIO FZQ028 (Zeng et al., 2020b), and they showed moderate antimicrobial activities against *S. aureus* ATCC 29213 and *Bacillus thuringiensis* ATCC 10792, with inhibitory diameters from 7.49 to 8.94 mm at 30 μg/disk, respectively. Butyrolactone I (**189**) displayed significant antibacterial against *S. aureus* with the MIC value of 0.78 μg/mL, which was collected from the fungus *Aspergillus* sp. SCSIO 41029 (Chen et al., 2021). A new aromatic butanolide, asperbutenolide D (**190**), along with two known analogs (+)-3′,3′-di-(dimethylallyl)-butyrolactone II (**191**) and aspernolide E (**192**), displayed moderate antibacterial against *S. aureus* with the MIC values of 21.3, 17.4, and 26.1 μM, respectively, which were separated from sediment-associated fungus *A. terreus* SCAU011 (Bao et al., 2021). A novel butyrolactone derivative, flavipesin A (**193**), demonstrated obvious antibacterial activities against *S. aureus* (MIC, 8.0 μg/mL) and *B. subtillis* (MIC, 0.25 μg/mL), and the fungus was separated from the mangrove-associated fungus *A. flavipes* AIL8 (Bai et al., 2014). Versicolactone B (**194**) and butyrolactone VI (**195**) were separated from the coral-derived fungus *A. terreus* SCSIO41404 (Peng et al., 2022). Compound **194** demonstrated weak antibacterial against *E. faecalis* (MIC, 5 μg/mL). Compound **195** demonstrated weak antibacterial against *K. pneumoniae* (MIC, 50 μg/mL). A novel aromatic butanolide, asperbutenolide A (**196**), with strong inhibition activity against *S. aureus* (MIC, 1.30 μg/mL) and *V. splendidus* (MIC, 3.70 μg/mL), was separated from the mangrove sediment-derived fungus *A. terreus* SCAU011 (Bao et al., 2020). 5*R*-(+)-9-hydroxymicroperfuranone (**197**) and 5*R*-(+)-microperfuranone (**198**), with weak inhibition activity against *E. coli* with the MIC values of 50 and 25 μg/mL, respectively, which were separated the fungus *Aspergillus* sp. ZZ1861 (Ha et al., 2024). Two new benzyl pyrones, asperpyranones A–B (**199**–**200**), exhibited weak antibacterial against *P. aeruginosa* ATCC 27853 with the MIC values of 32 and 128 μg/mL, respectively, which were separated from a marine-derived fungus *A. terreus* RA2905 (Wu et al., 2020b). Nectriapyrone (**201**) and asperisocoumarin A (**202**), displayed a weak antibacterial effect on *V. harveyi* with MIC values of 64.0 and 32.0 μg/mL, respectively, which were separated from the fungus *Aspergillus* sp. LS53 (Zhang L. et al., 2020; Zhang Y. H. et al., 2020). Unguinol (**203**), 2-chlorounguinol (**204**), and nidulin (**205**) showed strong antibacterial activity against *E. coli*, *P. aeruginosa*, *S. aureus*, *E. faecalis*, *B. subtilis*, *Salmonella*. *typosa*, *Vibrio cholera* Inaba, and *M. luteus*, with MIC values ranging 0.78–3.12 μg/disk, which were separated from the fungus *Aspergillus unguis* WR8 (Handayani et al., 2020). One novel depsidone derivative, aspergillusidone H (**206**), together with three known compounds nornidulin (**207**), aspergillusidones B (**208**), and C (**209**), were separated from the fungus *A. unguis* GXIMD02505 (Zhang Y. T. et al., 2022). Compounds **207** and **209** had antibacterial activity against MRSA, *Mylabris* var*iabilis*, and *Methanocaldococcus jannaschii*, with MIC values from 2 to 32 μg/mL. Compound **208** displayed antibacterial activity against *M. variabilis* (MIC, 128 μg/mL). One new depsidone 7-dechloronidulin (**210**), together with two known compounds 2,4-dichlorounguinol (**211**) and emeguisin B (**212**) were separated from the fungus *A. unguis* GXIMD02505 (Thi et al., 2023). Compound **210** was selectively bioactive on three Gram-positive bacteria (*B. cereus*, *E. faecalis*, *S. aureus*) (MICs: 2–4 μg/mL). Compound **211** had broad-spectrum antimicrobial activity against six bacteria (*B. cereus*, *E. faecalis*, *S. aureus*, *E. coli*, *P. aeruginosa*, and *S. enterica*), with the MIC values ranging 16–64 μg/mL. Compound **212** showed weak activity against *E. faecalis* with the MIC value of 256 μg/mL. One new depsidone asperunguissidone A (**213**), one new phthalide asperunguislide A (**214**), and six known compounds asperlide (**215**), aspergiside C (**216**), (3*S*)-3-ethyl-5,7-dihydroxy-3,6-dimethylphthalide (**217**), aspergisidone (**218**), folipastatin (**219**), emeguisins A (**220**), were separated from the fungus *A. unguis* PSU-MF16 (Saetang et al., 2021). Compounds **213**–**220** showed activity against *S. aureus* and MRSA with the MIC values from 1.0 to 200.0 μg/mL. 8-Demethoxy-10-methoxy-wentiquinone C (**221**) was separated from the fungus *A. sydowii* C1-S01-A7, and showed a weak antibacterial activity against MRSA with an MIC value of 32.4 μg/mL (Wang et al., 2019). Three new farnesylated phthalide derivatives farnesylemefuranones D–F (**222**–**224**) were isolated from the cold-seep-derived fungus *A. insuetus* SD-512, and they exhibited inhibitory effects against *V. vulnificus* with the same MIC value of 4.0 μg/mL, while **221** and **223** also inhibited *V. alginolyticus* with the same MIC value of 4.0 μg/mL (Chi et al., 2020). Silvaticol (**225**) was separated from the fungus *Aspergillus* sp. ZZ1861, and **225** displayed inhibitory activity against *E. coli* with the MIC value of 12.5 μg/mL (Ha et al., 2024). Two novel dihydroisocoumarin derivatives, aspergillumarins A (**226**) and B (**227**), were separated from the marine-associated fungus *Aspergillus* sp. (Li et al., 2012). Compounds **226** and **227** demonstrated weak antibacterial against *S. aureus* and *B. subtilis* at a concentration of 50 μg/mL. A new dihydroisocoumarin, aspergimarin G (**228**), was separated from the sponge-associated fungus *Aspergillus* sp. NBUF87 (Lin S. X. et al., 2023), and showed a moderate activity against *S. aureus* and *S. enteritidis* with MIC values from 16.0 to 64.0 μg/mL. (*R*)-3-Hydroxymellein (**229**) and (3*R*,4*S*)-trans-4-hydroxymellein (**230**) were separated from the fungus *Aspergillus* sp. SCSCIO41405 (Peng et al., 2021). Compound **229** demonstrated a weak antibacterial effect on MRSA (MIC, 100.0 μg/mL). Compound **230** displayed a weak antibacterial effect on *E. faecalis* (MIC, 100.0 μg/mL). Three new 4-hydroxy-*α*-pyrones nipyrones A–C (**231**–**233**) and one known analog germicidin C (**234**) were separated from the sponge-associated fungus *A. niger* LS24 (Ding et al., 2019). Compound **233** demonstrated a significant inhibitory effect on *S. aureus* and *B. subtilis* with the MIC values of 8.0 and 16.0 μg/mL, respectively. Sartorypyrone A (**235**) was separated from the fungus *Aspergillus* sp. WHUF03110 and displayed a strong inhibitory activity against *B. subtilis*, *S. aureus* ATCC25923, *S. aureus* NEWMAN, *S. aureus* USA300, and *S. aureus* NRS 271 with MIC values ranging 1.0–2.0 μg/mL (Lv et al., 2021). Asperochrin A (**236**), chlorohydroaspyrones A (**237**) and B (**238**), were separated from the mangrove-associated fungus *spergillus ochraceus* MA-15 (Liu et al., 2015). Compound **236** showed an inhibitory activity against *A. hydrophila*, *V. anguillarum*, and *V. harveyi* with the MIC values of 8.0, 16.0, and 8.0 μg/mL, respectively. **237** and **238** showed weak inhibitory activity against the above three pathogenic bacterial (MIC, 16–32 μg/mL). One novel penicillide analog, ∆^2^′-1′-dehydropenicillide (**239**) and a known analog dehydropenicillide (**240**), were separated from the fungus *Aspergillus* sp. IMCASMFI80035 (Song F. H. et al., 2021), which demonstrated significant antibacterial activities against *H. pylori* (MIC, 21.73 and 21.61 μM, respectively) (Figure 11).

#### 2.3.4 Other polyketide metabolites

**Figure 12:**
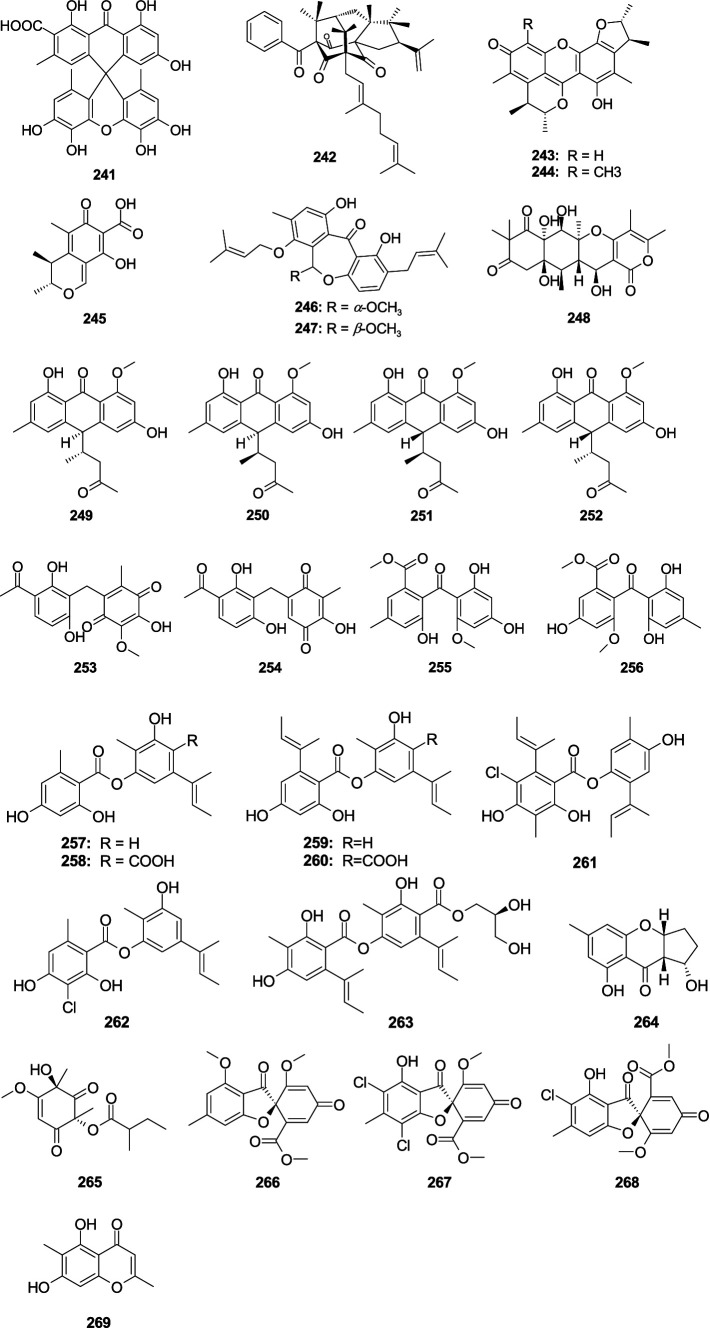
Chemical structures of other antibacterial polyketide metabolites **241**–**269** from *Aspergillus* spp.

The novel compound aspergiloxathene A (**241**), separated from the marine-associated fungus *Aspergillus* sp. IMCASMF180035, exhibited significant antibacterial activities against *S. aureus* (MIC, 5.60 μM) and MRSA (MIC, 22.40 μM) (Song F. H. et al., 2021). A new compound, cowabenzophenone A (**242**), was separated from the mangrove-associated fungus *A. terreus* (Ukwatta et al., 2020). Compound **242** showed strong antibacterial activity against *B. subtilis* (MIC, 1.0 μg/mL) and *S. aureus* (MIC, 2.0 μg/mL). Penicitrinone A (**243**), penicitrinone *F* (**244**), and citrinin (**245**) showed weak activity against *E. ictaluri* and *V. alginolyticus* with the MIC values from 16.0 to 32.0 μg/mL, were separated from the fungal *A. sydowii* EN-534 (Yang et al., 2018b). Two new compounds 25*S*-*O*-methylarugosin A (**246**), 25*R*-*O*-methylarugosin A (**247**) were separated from the fungus *Aspergillus* sp. ZZ1861 (Ha et al., 2024). Compound **247** showed weak activities against MRSA (MIC, 50.0 μg/mL). The new compound 12*S*-aspertetranone D (**248**), separated from sea trench-derived fungus *Aspergillus* sp. SY2601 (Sun et al., 2024), exhibited antibacterial effects on MRSA and *E. coli* with the MIC values of 3.75 and 5.0 μg/mL, respectively. Four new anthraquinone derivatives, (10*S*,12*S*)-chevalierone, (10*S*,12*R*)-chevalierone, (10*R*,12*S*)-chevalierone, and (10*R*,12*R*)-chevalierone (**249**–**252**), were isolated from the fungus *A. chevalieri* HP-5 (Wang Q. Y. et al., 2022). Compounds **250**–**252** showed significant inhibition against the opportunistic pathogenic bacterium *P. aeruginosa* (inhibition rate: 81.0–91.5%) and MRSA (inhibition rate: 74.0–88.5%) at the concentration of 200 μM, while the structural congener compound **249** only showed weak inhibition (inhibition rate: 38.2%) against the *P. aeruginosa* at 200 μM. Two novel phenome compounds, asperphenones A (**253**) and B (**254**), were separated from the mangrove-derived fungus *Aspergillus* sp. YHZ-1 (Guo et al., 2018). Compounds **253** and **254** demonstrated weak antibacterial effects on four Gram-positive bacteria, *S. aureus*, *S. pyogenes*, *B. subtilis*, and *M. luteus*, with the MIC values from 32.0 to 64.0 μg/mL. One new compound penibenzophenone E (**255**) and a known compound sulochrin (**256**) were originated from the fungus *A. fumigatus* H22 (Zhang R. et al., 2022). Compounds **255** and **256** demonstrated activity against MRSA with the same MIC value of 1.25 μM. Aspergisides A–B (**257**–**258**), together with agonodepsides A–B (**259**–**260**), were separated from sponge-derived fungus *A. unguis* PSU-MF16 (Saetang et al., 2021). Compounds **257**, **259**, and **260** had strong antibacterial activity against *S. aureus* and MRSA with the MIC values from 2.0 to 16.0 μg/mL. Compound **258** displayed a weak activity against *S. aureus* and MRSA with the same MIC value of 200.0 μg/mL. Guisinol (**261**) was separated from the fungus *A. unguis* GXIMD 02505 (Zhang Y. T. et al., 2022). Compound **261** showed antibacterial activities against MRSA (MIC, 16.0 μg/mL) and *M.* var*iabilis* (MIC, 64.0 μg/mL). Two new phenolic polyketides, unguidepside C (**262**) and agonodepside C (**263**), were isolated from two marine-associated fungal strains of *A. unguis* (Anh et al., 2022). Compounds **262** and **263** demonstrated inhibitory effects against *S. aureus*, *M. luteus*, and *B. subtilis*, with the MIC values from 8.0 to 22.1 μM. One new chromone, aspergilluone A (**264**), was separated from the fungus *Aspergillus* sp. LS57, which displayed an antibacterial effect on *M. tuberculosis* (MIC, 32.0 μg/mL) and *S. aureus* (MIC, 64.0 μg/mL) (Liu et al., 2021). Phomaligol A (**265**), separated from the fungus *A. flavus* MFA500, displayed a weak activity against *S. aureus* with MIC value of 31.2 μg/mL (Yang et al., 2011). Trypacidin (**266**) showed significant antitubercular activity with the MIC value of 1.25 μg/mL, which was separated from the fungus *A. fumigatus* MF029 (Song Z. J. et al., 2021). (+)-Geodin (**267**) and chlorotrypacidin (**268**) showed a weak antibacterial effect on *Staphylococcus albus*, *S. aureus*, and *V. anguillarum* with the same MIC value of 25.0 μM, and they were separated from the fungi of *A. versicolor* TA01-14 (Zhang et al., 2019). Eugenitol (**269**) demonstrated weak inhibitory activity against MRSA with the MIC value of 485.4 μM, which was separated from the mangrove sediment-associated fungus *Aspergillus* sp. SCSIO41407 (Cai et al., 2021) (Figure 12).

### 2.4 Steroids

Steroids were biosynthesized through complex cyclization reactions involving squalene and mevalonate pathways. A total of 18 antibacterial steroids (including 11 new compounds) were identified from marine-derived *Aspergillus* species. The steroid structures and the absolute configurations of the new compounds were elucidated by a detailed spectroscopic analysis of NMR and MS data, optical rotatory dispersion, ECD calculations, and single-crystal X-ray diffraction.

**Figure 13:**
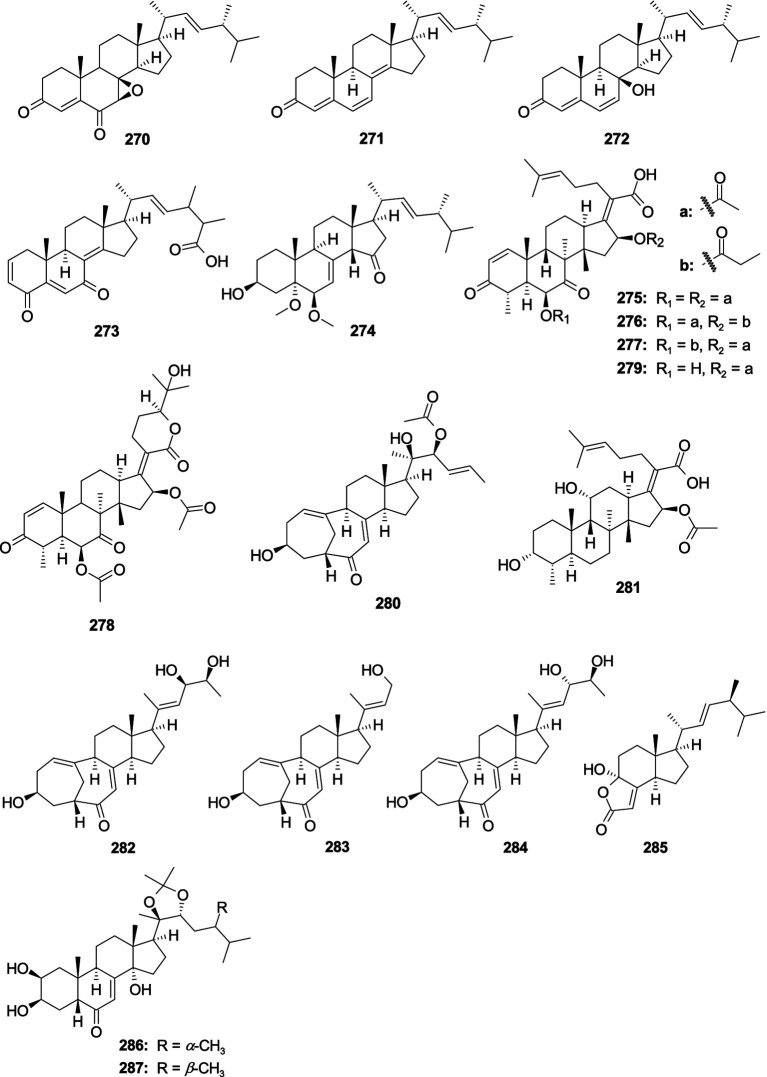
Chemical structures of antibacterial steroids **270**–**287** from *Aspergillus* spp.

A new steroid 7*β*,8*β*-Epoxy-(22*E*,24*R*)-24-methylcholesta-4,22-diene-3,6-dione (**270**) and a known steroid ergosta-4,6,8(14),22-tetraene-3-one (**271**) were separated from the fungus *Aspergillus penicillioides* SD-311 (Chi et al., 2021b). Compound **270** showed antibacterial activity against *V. anguillarum* with the MIC value of 32.0 μg/mL, while **271** displayed inhibitory activity against *E. tarda* and *M. luteus* with the same MIC value of 16.0 μg/mL. One new ergosterol derivative, isocyathisterol (**272**), exhibited a weak antibacterial activity against *E. coli* and *S. aureus*, with inhibitory diameters of 6.7 and 5.7 mm at 30 μg/disk, respectively, was originated from the alga-derived fungus *A. ustus cf*-42 (Liu et al., 2014). One new oxygenated steroid, aspersteroid A (**273**), was isolated from the marine-derived fungus *A. flavus* YJ07-1 (Yang M. Y. et al., 2018). Compound **273** showed antibacterial activities against *V. anguillarum*, *V. parahemolyticus*, and *V. alginolyticus* with the same MIC value of 12.5 μM. One new oxygenated ergostane-type steroid, 3*β*-hydroxy-5*ɑ*,6*β*-methoxyergosta-7,22-dien-15-one (**274**), was isolated from the marine sponge-derived fungus *Aspergillus* sp. NR151817 (Wen et al., 2024). Compound **274** showed weak inhibitory activity against *S. aureus* with an MIC value of 64 μg/mL. A known steroid C-21 acid helvolic acid (**275**) was isolated from the fungus *Aspergillus* sp. SCS-KFD66 (An et al., 2018). Compound **275** exhibited strong activity against *S. aureus* ATCC 6538 with an MIC value of 2.0 μg/mL. Three new helvolic acid derivatives, 16-*O*-propionyl-16-*O*-deacetylhelvolic acid (**276**), 6-*O*-propionyl-6-*O*-deacetylhelvolic acid (**277**), and 24-epi-6*β*,16*β*-diacetoxy-25-hydroxy-3,7-dioxo-29-nordammara-1,17(20)-diene-21,24-lactone (**278**), were isolated from the marine-derived fungus *A. fumigatus* HNMF0047 (Kong et al., 2018). Compounds **276**–**278** showed antibacterial activities against *Streptococcus agalactiae* and *S. aureus* with MIC values ranging 2.0–64.0 μg/mL. A new steroid 3,7-diketo-cephalosporin P_1_ (**279**), along with a known analog 22-*O*-acetylisocyclocitrinol A (**280**), were isolated from deep sea-derived fungus *A. fumigatus* SCSIO 41012 (Limbadri et al., 2018). Compound **279** showed weak activity against *A. baumanii* 19,606 with the MIC value of 50.0 μg/mL. Compound **280** exhibited high antibacterial activity with *A. baumanii* ATCC15122 and *K. pneumonia* ATCC14578 with the MIC values of 12.5 and 3.12 μg/mL, respectively. Fusidic acid (**281**) and neocyclocitrinol D (**282**) were obtained from the marine-derived fungus *A. flavus* JK07-1 (Ren et al., 2020). Compound **281** showed significant inhibitory activities against *Micrococcus lysodeikticus*, *B. cereus*, *Bacillus megaterium*, *Bacillus Anthracis*, and *Salmonella typhi*, with the MIC values of 0.07, 0.07, 0.07, 0.30, and 0.60 μM, respectively. Compound **282** showed effective inhibitory activity against *M*. *lysodeikticus* with an MIC value of 1.30 μM. A new C-23 steroid with bicyclo[4.4.1]A/B ring aspergillsteroid A (**283**) and a known analog neocyclocitrinol B (**284**) exhibited antibacterial activity against *V. harveyi* KP635244 with the MIC values of 16.0 and 128.0 μg/mL, respectively, which were separated from marine-derived fungus *Aspergillus* sp. LS116 (Xu P. et al., 2020). Demethylincisterol A_2_ (**285**) was separated from the coral-derived fungus *A. hiratsukae* SCSIO 5Bn_1_003 (Zeng et al., 2022a). Compound **285** displayed strong activity against *B. subtilis* with the MIC value of 10.26 μg/mL. Two new polyhydroxylated mycoecdysteroids, punicesterones B (**286**) and C (**287**), were separated from the deep-sea-derived fungus *A. puniceus* SCSIO z021 (Huang et al., 2023). Compounds **286** and **287** could show significantly inhibitory activity against *S. iniae*, *S. agalactiae*, *E. coli*, *B. subtilis*, and *S. aureus* at a concentration of 0.132 mM (Figure 13).

### 2.5 Other classes

Additionally, there were also some other classes of antibacterial secondary metabolites isolated from *Aspergillus* spp., including fatty acids, glycosides, and benzene derivatives. A total of 50 antibacterial compounds (including 14 new compounds) were isolated from the *Aspergillus* spp. The structures, like three undescribed compounds, carnemycins H − I and stromemycin B, were elucidated by comprehensive spectroscopic data and *J*-based configurational analysis.

**Figure 14:**
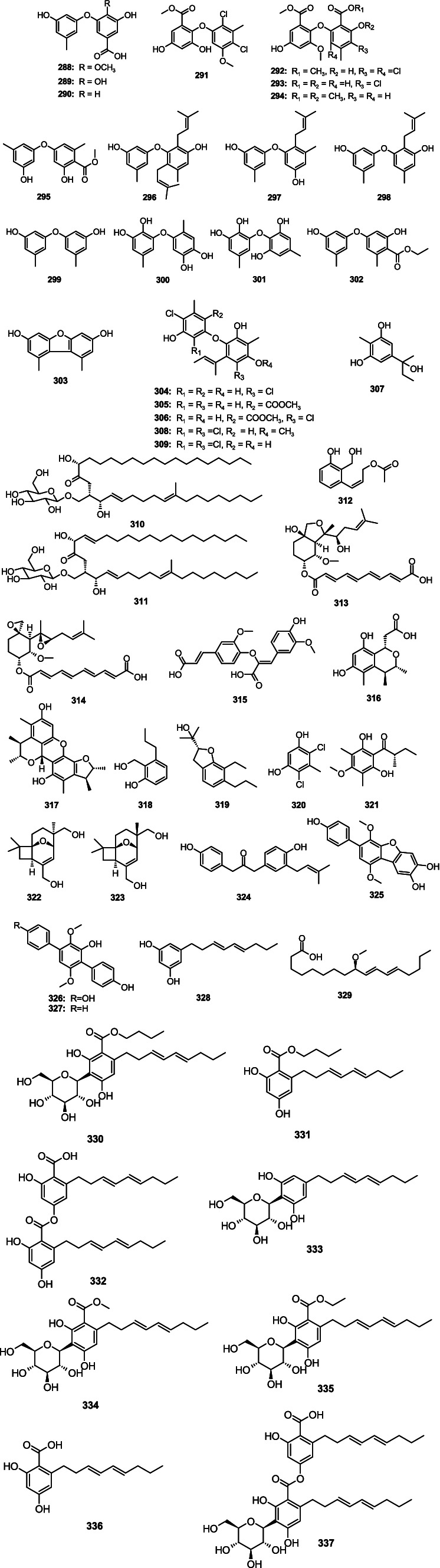
Chemical structures of other antibacterial classes **288**–**337** from *Aspergillus* spp.

A new phenyl ether derivative, 3-hydroxy-5-(3-hydroxy-5-methylphenoxy)-4-methoxybenzoic acid (**288**), together with two known analogs 3,4-dihydroxy-5-(3-hydroxy-5-methylphenoxy)benzoic acid (**289**) and 3-hydroxy-5-(3-hydroxy-5-methylphenoxy)-benzoic acid (**290**), were separated from the marine-derived fungus *A. carneus* (Xu et al., 2017). Compounds **288**–**290** had weak activity against *S. aureus*, *V. anguillarum*, and *E. coli* with the same MIC value of 25 μM. A new compound aspergetherin C (**291**) and two known analogs, methyl 3,5-dichloroasterric acid (**292**) and methyl chloroasterrate (**293**), were isolated from the fungus *A. terreus* 164,018 (Li J. X. et al., 2023). Compounds **291** and **293** showed weak antibacterial activity against MRSA 05–72 and MRSA USA300 (MIC, 64.0 μg/mL). Compound **292** had strong inhibitory activity against MRSA 05–72 with the MIC value of 1.0 μg/mL. Dimethyl 2,3′-dimethylosoate (**294**) was isolated from *A. fumigatus* H22 (Zhang R. et al., 2022). Compound **294** showed strong inhibitory activity against MRSA with the same MIC value of 5.0 μM. 4-Methoxycarbonyldiorcinol (**295**), showed strong inhibitory activity against *P. aeruginosa* with the MIC value of 13.9 μM, which was separated from the marine algae-derived fungus *A. versicolor* OUCMDZ-2738 (Liu et al., 2019). One new diphenyl ether, diorcinol K (**296**), along with two known analog diorcinols D (**297**) and I (**298**), were isolated from a fungus *Aspergillus* sp. CUGB-F046 (Xu et al., 2018). Compounds **296**–**298** displayed significant antibacterial activity against *S. aureus* and MRSA with the MIC values from 3.13 to 6.25 μg/mL. Diorcinol (**299**) was isolated from the deep-sea-derived *A. versicolor* 170,217 (Lin S. H. et al., 2023). Compound **299** exhibited weak inhibitory activity against *V. parahemolyticus* with an MIC value of 128.0 μg/mL. Violaceol-I (**300**), violaceol-II (**301**), 4-carbethoxydiorcinal (**302**), and 1,9-dimethyl-3,7-dibenzofurandiol (**303**) were isolated from the fungus *Aspergillus* sp. ZZ1861 (Ha et al., 2024). Compounds **300**–**303** showed inhibitory activity against MRSA and *E. coli* with the MIC values from 6.25 to 50.0 μg/mL. Two new diphenyl ethers, aspergillusethers E (**304**) and *F* (**309**), together with three known compounds aspergillusethers C (**305**) and D (**306**) and pilobolusate (**307**), were isolated from sponge-derived fungus *Aspergillus* sp. PSU-MF16 (Saetang et al., 2021). Compound **304** demonstrated moderate inhibitory activity against *S. aureus* and MRSA with the same MIC value of 16.0 μg/mL. Compounds **305**–**307** had weak antibacterial activity against *S. aureus* and MRSA with MIC values from 64.0 to 128.0 μg/mL. Aspergillusethers J (**308**) and F (**309**) showed inhibitory activity against MRSA, *M.* var*iabilis*, and *M. jannaschii* with MIC values ranging 2.0–64.0 μg/mL, which were separated from coral-derived fungus *A. unguis* GXIMD 02505 (Zhang Y. T. et al., 2022). Two new cerebroside derivatives, flavusides A (**310**) and B (**311**), were isolated from the marine-derived fungus *A. flavus* MFA500 (Yang et al., 2011). Compounds **310** and **311** showed moderate inhibitory activity against *S. aureus* with the same MIC value of 15.6 μg/mL. One new phenol derivative, acetylpeniciphenol (**312**), showed activity against *E. tarda*, *V. alginolyticus*, and *V. vulnificus* with the MIC values of 4.0, 8.0, and 8.0 μg/mL, respectively, which was separated from the cold-seep-derived fungus *A. insuetus* SD-512 (Chi et al., 2021a). Fumagiringillin (**313**) and fumagillin (**314**) were isolated from the marine-derived fungus *A. fumigatus* H22 (Zhang R. et al., 2022). Compounds **313** and **314** showed inhibitory activity against MRSA with MIC values of 25.0 and 2.50 μg/mL, respectively. 8-*O*-4-dehydrodiferulic acid (**315**) was isolated from the sponge-derived fungus *Aspergillus* sp. (Zhou et al., 2014). Compound **315** displayed activity against *R. litoralis* with an MIC value of 1.0 μg/mL. A new citrinin monomer penicitrinol L (**316**) and a known compound penicitrinol A (**317**) were separated from the marine algal-derived fungus *A. sydowii* EN-534 (Yang et al., 2018b). Compound **316** displayed weak inhibitory activity against *E. coli*, *E. ictaluri* and *V. alginolyticus* with the same MIC value of 64.0 μg/mL. Compound **317** showed inhibitory activity against *E. coli*, *M. luteus*, *E. ictaluri*, *V. alginolyticus*, and *V. parahaemolyticus* with the MIC values from 4.0 to 32.0 μg/mL. 2-(Hydroxymethyl)-3-propylphenol (**318**) and (−)-brassicadiol (**319**) were separated from the mangrove-derived fungus *Aspergillus* sp. ZJ-68 (Cai et al., 2019). Compounds **318** and **319** showed strong activity against *S. aureus*, *E. coli* and *B. subtilis* (MIC, 4.15–12.5 μg/mL). 4,6-Dichloro-5-methylbenzene-1,3-diol (**320**) was isolated from deep-sea derived fungus *A. terreus* CC-S06-18 (Huang et al., 2024). Compound **320** showed inhibitory activity against *V. parahaemolyticus* ATCC 17802, exhibiting an MIC value of 7.8 μg/mL. 1-(2,6-Dihydroxy-4-methoxy-3,5-dimethylphenyl)-2-methylbutan-1-one (**321**) was isolated from *A. unguis* GXIMD 02505 (Zhang Y. T. et al., 2022). Compound **321** showed inhibitory activities against *M. variabilis* and *M. jannaschii* with MIC values of 8.0 and 32.0 μg/mL, respectively. Two novel compounds, asperporonins A (**322**) and B (**323**), were separated from a marine fungus *A. terreus* SCSIO 41202 (Zhang et al., 2024). Compounds **322** and **323** showed antibacterial effects against *X. citri* subsp. *citri* with the same MIC value of 0.3125 mg/mL. Terrusnolide A (**324**) was separated from the deep-sea-derived fungus *Aspergillus* sp. SCSIO 41029 (Chen et al., 2021). Compound **324** displayed inhibitory activity against *S. aureus* with an MIC value of 6.25 μg/mL. Candidusin A (**325**), terphenyllin (**326**), and 4″-deoxyterphenyllin (**327**) were separated from a coral-derived fungus *Aspergillus* sp. SCSIO40435 (Ye et al., 2022). Compound **325** showed antibacterial activities against *E. coli*, *A. baumannii*, *S. aureus*, and MRSA with the MIC values of 1.0, 64.0, 32.0, and 16.0 μg/mL, respectively. Compound **326** had strong antibacterial activity against *E. coli* with an MIC value of 0.5 μg/mL. Compound **327** exhibited weak inhibitory activity against *B. subtilis* and *M. luteus* with MIC values of 64.0 and 32.0 μg/mL, respectively. 5[(3*E*,5*E*)-nona-3,5-dien-1-yl]benzene (**328**) was separated from the sponge-associated fungus *A. stellatus* KUFA2017 (Machado et al., 2022). Compound **328** showed antibacterial activity against *E. faecalis* ATCC 29212, *E. faecalis* B3/101 (VRE), *S. aureus*, and MRSA with the MIC values of 16.0, 16.0, 32.0, and 16.0 μg/mL, respectively (9*R*,10*E*,12*E*)-9-methoxyoctadecadienoic acid (**329**) was separated from a marine fungus *A. terreus* SCSIO41202 (Zhang et al., 2024). Compound **329** showed an antibacterial effect against *X. citri* subsp. *citri* with an MIC value of 0.078 mg/mL. Three undescribed compounds, carnemycins H–I (**330**–**331**) and stromemycin B (**332**), together with six phenolic compounds carnemycin E (**333**), carnemycin B (**334**), carnemycin A (**335**), 2,4-dihydroxy-6-[(3*E*,5*E*)-nona-3,5-dien-1-yl]-benzoic acid (**336**), and stromemycin (**337**), were separated from marine-derived fungus *A. ustus* (Xue et al., 2024). Compounds **330**–**337** showed different inhibitory activity against *R. solanacearum* with MIC values from 3 to 35 μg/mL (Figure 14).

## 3 Comprehensive overview and conclusions

In recent years, marine fungi have become a research hotspot because they can produce bioactive compounds. In conjunction with a series of previous literature, we conducted a comprehensive study focusing on antimicrobial compounds produced by *Aspergillus* fungi from different marine origins between January 2010 and June 2024 in Table 1.

**Table 1:** The antibacterial activity of secondary metabolites 1–331 from *Aspergillus* sp.

Compounds	Producing strains	Habitats	Genbank accession number	Antibacterial activity the MIC values	References
(5*S*,6*S*)-16,17-Dihydroophiobolin H (**1**)	*A. insuetus* SD-512	Cold-seep sediment, the northeast of the South China Sea	MN650839	Anti-A. hydrophilia, *E. coli*, *E. tarda*, *P. aeruginosa*, *V. alginolyticus*, V anguillarum, V. parahemolyticus, and *V. vulnificus*; 4, 4, 4, 8, 4, 32, 4, and 8 μg/mL	Chi et al. (2020)
(6*α*)-21,21-*O*-dihydroophiobolin G (**2**)	*A. insuetus* SD-512	Cold-seep sediment, the northeast of the South China Sea	MN650839	Anti-A. hydrophilia, *E. coli*, *E. tarda*, *P. aeruginosa*, *V. alginolyticus*, V anguillarum, V. parahemolyticus, and *V. vulnificus*; 8, 16, 8, 8, 4, 32, 8, and 8 μg/mL	Chi et al. (2020)
6-epi-Ophiobolin G (**3**)	*A. insuetus* SD-512	Cold-seep sediment, the northeast of the South China Sea	MN650839	Anti-A. hydrophilia, *E. coli*, *E. tarda*, *P. aeruginosa*, *V. alginolyticus*, V anguillarum, V. parahemolyticus, and *V. vulnificus*; 8, 16, 8, 8, 4, 32, 8, and 8 μg/mL	Chi et al. (2020)
Ophiobolin U (**4**)	*A. ustus* cf-42	Marine green alga, the Zhoushan Island, Zhejiang province, China	JX036023	Weak (anti-*E. coli* and *S. aureus*); Inhibitory diameters of 15 and 10 mm at 30 μg/disk	Liu et al. (2013)
(5*α*,6*α*)-Ophiobolin H (**5**)	*A. ustus* cf-42	Marine green alga, the Zhoushan Island, Zhejiang province, China	JX036023	Weak (anti-*E. coli*); Inhibitory diameter of 10 mm at 30 μg/disk	Liu et al. (2013)
Asperophiobolin E (**6**)	*A. hiratsukae* SCSIO 5Bn_1_003	Marine coral, the South China Sea	KY806121.1	Anti-*B. subtilis* and *S. aureus*; 17.0 and 102.86 μg/mL	Zeng et al. (2022a)
Asperbrunneo acid (**7**)	A. brunneoviolaceus MF180246	Mangrove mud sample, the Xinglin Bay, Xiamen, China	–	Anti-*S. aureus*; 200 μg/mL	Xu et al. (2024)
Aspergilol C (**8**)	*Aspergillus* sp. ZZ1861	Sea mud sample, the Zhoushan Island, Zhejiang province, China	OR985107	Anti-*E. coli*; 3.12 μg/mL	Ha et al. (2024)
Punctaporonin B (**9**)	*A. terreus* SCSIO 41202	Deep-sea sediment, the coast of the South China Sea	MN613535	Anti-*X. citri* subsp. *citri*; 0.625 mg/mL	Zhang et al. (2024)
Punctaporonin D (**10**)	*A. terreus* SCSIO 41202	Deep-sea sediment, the coast of the South China Sea	MN613535	Anti*-X. citri* subsp. *citri*; 0.625 mg/mL	Zhang et al. (2024)
Punctaporonin G (**11**)	*A. terreus* SCSIO 41202	Deep-sea sediment, the coast of the South China Sea	MN613535	Anti-*X. citri* subsp. *citri*; 0.3125 mg/mL	Zhang et al. (2024)
Sesquiterpenoid (**12**)	*A. versicolor* SD-330	Marine sediment, the South China Sea	MN176407	Anti-*E. coli*, A. hydrophilia, *E. tarda*, *P. aeruginosa*, *V. harveyi*, and *V. parahaemolyticus*; 8, 8, 8, 8, 4, and 16 μg/mL	Li et al. (2021)
Aspergoterpenin C (**13**)	*A. versicolor* SD-330	Marine sediment, the South China Sea	MN176407	Anti-*E. coli*, A. hydrophilia, *E. tarda*, *P. aeruginosa*, *V. harveyi*, and *V. parahaemolyticus*; 2, 8, 4, 16, 8, and 8 μg/mL	Li et al. (2021)
Engyodontiumone I (**14**)	*A. versicolor* SD-330	Marine sediment, the South China Sea	MN176407	Anti-*E. coli*, A. hydrophilia, *E. tarda*, *P. aeruginosa*, *V. harveyi*, and *V. parahaemolyticus*; 1, 4, 4, 16, 4, and 8 μg/mL	Li et al. (2021)
Aspergillusene B (**15**)	A. sydowii LW09	Deep-sea sediment, the Southwest Indian Ridge	OP584347	Anti-*R. solanacarum*; 32 μg/mL	Yang et al. (2023)
(7*S*,11*S*)-(+)-12-Hydroxysydonic acid (**16**)	A. sydowii LW09	Deep-sea sediment, the Southwest Indian Ridge	OP584347	Anti-*P. syringae*; 32 μg/mL	Yang et al. (2023)
Expansol G (**17**)	A. sydowii LW09	Deep-sea sediment, the Southwest Indian Ridge	OP584347	Anti-*R. solanacarum*; 32 μg/mL	Yang et al. (2023)
(*S*)-Sydonic acid (**18**)	A. sydowii LW09	Deep-sea sediment, the Southwest Indian Ridge	OP584347	Anti-*R. solanacarum*; 32 μg/mL	Yang et al. (2023)
Asperolide D (**19**)	A. wentii SD-310	Deep-sea sediment, the South China Sea	KM409566	Anti-*E. tarda*; 16 μg/mL	Li et al. (2016)
Asperolide A (**20**)	A. wentii SD-310	Deep-sea sediment, the South China Sea	KM409566	Anti-*E. tarda*; 16 μg/mL	Li et al. (2016)
Sphaeropsidin A (**21**)	*A. porosus* G23	Marine alga, the marine environment by BioViotica Naturstoffe GmbH	LT671130.1	Anti-*S. aureus* ATCC 25923 and ATCC BAA-41; 32.6 and 35.3 μM	Neuhaus et al. (2019)
Aspergiloid E (**22**)	*A. porosus* G23	Marine alga, the marine environment by BioViotica Naturstoffe GmbH	LT671130.1	Anti-*S. aureus* ATCC 25923 and ATCC BAA-41; 71.6 and 77.8 μM	Neuhaus et al. (2019)
Aspergillactone (**23**)	*Aspergillus* sp. CSYZ-1	Sediment, the Zhoushan Island, the East China Sea	–	Aanti-*H. pylori* ATCC 43504, G27, Hp159, BY583 and *S. aureus* ATCC 25923, USA300, BKS231, BKS233; 2, 1, 1, 4, 16, 2, 4, and 8 μg/mL	Cen et al. (2021)
Chevalone B (**24**)	Aspergillus sp. H30	*Cucumaria japonica*, the South China Sea	–	Weak (anti-*S. aureus*)	Hu et al. (2019)
Chevalone H (**25**)	*A. hiratsukae* SCSIO 7S2001	Marine gorgonian coral, the South China Sea	MN347034	Anti-*M. lutea*, *K. pneumoniae*, MRSA, and *S. faecalis*; 6.25, 50, 6.25, and 6.25 μg/mL	Chen X. Y. et al. (2022)
Chevalone I (**26**)	*A. hiratsukae* SCSIO 7S2001	Marine gorgonian coral, the South China Sea	MN347034	Anti-*M. lutea*, MRSA, and *S. faecalis*; 25, 6.25, and 25 μg/mL	Chen X. Y. et al., 2022
Chevalone J (**27**)	*A. hiratsukae* SCSIO 7S2001	Marine gorgonian coral, the South China Sea	MN347034	Anti-*M. lutea*, *K. pneumoniae*, and MRSA; 25, 25, and 12.5 μg/mL	Chen X. Y. et al., 2022
Chevalone K (**28**)	*A. hiratsukae* SCSIO 7S2001	Marine gorgonian coral, the South China Sea	MN347034	Anti-*K. pneumoniae*, MRSA, and *S. faecalis*; 6.25, 25, and 50 μg/mL	Chen X. Y. et al., 2022
Chevalone L (**29**)	*A. hiratsukae* SCSIO 7S2001	Marine gorgonian coral, the South China Sea	MN347034	Anti-*M. lutea*, MRSA, and *S. faecalis*; 12.5, 12.5, and 12.5 μg/mL	Chen X. Y. et al., 2022
Austalide R (**30**)	Aspergillus sp.	Marine sponge, the Adriatic Sea	–	Anti-*H. aquamarina*, *P. irgensii*, *P. elyakovii*, *S. putrefaciens*, and *V. harveyi*; 0.1 μg/mL	Zhou et al. (2014)
Austalide M (**31**)	Aspergillus sp.	Marine sponge, the Adriatic Sea	–	Anti-*H. aquamarina*, *P. irgensii*, *P. elyakovii*, *R. litoralis*, *S. putrefaciens*, and *V. harveyi*; 0.001, 0.01, 0.001, 0.001, 0.001, and 0.001 μg/mL	Zhou et al. (2014)
Austalide N (**32**)	Aspergillus sp.	Marine sponge, the Adriatic Sea	–	Anti-V. natrieegens and R. litorails; 0.01 μg/mL	Zhou et al. (2014)
Griseofamine A (**33**)	*Aspergillus* sp. SCSIO 41024	Deep-sea sediment, the South China Sea	MH608347.1	Anti-*E. coli*; 64.0 μg/mL	Chen et al. (2020)
Brevianamide S (**34**)	*A. versicolor* MF030	Deep-sea sediment, the Bohai Sea, China	–	Anti-BCG; 6.25 μg/mL	Song et al. (2012)
Brevianamide T (**35**)	*A. versicolor* MF030	Deep-sea sediment, the Bohai Sea, China	–	Anti-BCG; 50 μg/mL	Song et al. (2012)
Brevianamide U (**36**)	*A. versicolor* MF030	Deep-sea sediment, the Bohai Sea, China	–	Anti-BCG; 25 μg/mL	Song et al. (2012)
Brevianamide V (**37**)	*A. versicolor* MF030	Deep-sea sediment, the Bohai Sea, China	–	Anti-BCG; 100 μg/mL	Song et al. (2012)
Brevianamide K (**38**)	*A. versicolor* MF030	Deep-sea sediment, the Bohai Sea, China	–	Anti-BCG; 50 μg/mL	Song et al. (2012)
Deoxybrevianamide E (**39**)	*A. versicolor* MF030	Deep-sea sediment, the Bohai Sea, China	–	Anti-BCG, *S. aureus* ATCC 6538, and *B. subtilis* ATCC 6633; 100, 100, and 50 μg/mL	Song et al. (2012)
9ξ-*O*-2(2,3-dimethylbut-3-enyl)-brevianamide Q (**40**)	*A. versicolor* pt20	Marine brown alga, the Pingtan Island, Fujian province, China	–	Weak (anti-*E. coli* and *S. aureus*); Inhibitory diameters of 7 and 7 mm at 30 μg/disk	Miao et al. (2012)
12,13-Dihydroxy-fumitremorgin C (**41**)	*Aspergillus* sp. SCSIO Ind09F01	Deep-sea sediment, the Indian Ocean	AY373869	Anti-*M. tuberculosis*; 2.41 μM	Luo et al. (2017)
	*A. fumigatus* H22	Seawater, the Western Pacific	–	Anti-MRSA and *M. bovis*; 2.50 and 25 μM	Zhang R. et al. (2022)
(−)-Stephacidin A (**42**)	*Aspergillus* sp. XS-20090066	Marine gorgonian coral, the South China Sea	HM535361	Anti-*S. epidermidis*; 14.5 μM	Chen et al. (2013)
Notoamide *F* (**43**)	*A. sclerotiorum* GDST-2013-0501	Marine sponge, the South China Sea	MT534582	Anti-*S. epidermidis*; 12.5 μM	Wang C. Y. et al. (2022)
Asperthrin A (**44**)	*Aspergillus* sp. YJ191021	The intertidal zone soil, the ZhouShan Island, Zhejiang province, China	–	Anti-*X. oryzae* pv., *E. tarda*, *V. anguillarum*, A. hydrophilia, and *V. parahaemolyticus*; 12.5, 16, 8, 32, and 16 μg/mL	Yang et al. (2021)
Asperthrin E (**45**)	*Aspergillus* sp. YJ191021	The intertidal zone soil, the ZhouShan Island, Zhejiang province, China	–	Weak (anti-*X. oryzae* pv.)	Yang et al. (2021)
24,25-Dihydroxyvariecolorin G (**46**)	*A. chevalieri* CS-122	Deep-sea cold-seep sediment, the northeast of the South China Sea	KU872171.1	Anti-*V. harveyi* and *E. coli*; 16 and 4 μg/mL	Yan et al. (2023)
25-Hydroxyrubrumazine B (**47**)	*A. chevalieri* CS-122	Deep-sea cold-seep sediment, the northeast of the South China Sea	KU872171.1	Anti-*V. harveyi*, *E. tarda*, *A. hydrophila*, *E. coli*, and *M. luteus*; 32, 16, 32, 16, and 32 μg/mL	Yan et al. (2023)
22-Chloro-25-hydroxyrubrumazine B (**48**)	*A. chevalieri* CS-122	Deep-sea cold-seep sediment, the northeast of the South China Sea	KU872171.1	Anti-*V. harveyi* and *E. coli*; 8 and 32 μg/mL	Yan et al. (2023)
25-Hydroxyvariecolorin *F* (**49**)	*A. chevalieri* CS-122	Deep-sea cold-seep sediment, the northeast of the South China Sea	KU872171.1	Anti-*V. harveyi* and *E. coli*; 32 μg/mL	Yan et al. (2023)
27-Epi-aspechinulin D (**50**)	*A. chevalieri* CS-122	Deep-sea cold-seep sediment, the northeast of the South China Sea	KU872171.1	Anti-*V. harveyi*, *E. tarda*, *A. hydrophila*, *E. coli*, and *M. luteus*; 16, 32, 32, 32, and 16 μg/mL	Yan et al. (2023)
Neoechinulin B (**51**)	*A. chevalieri* CS-122	Deep-sea cold-seep sediment, the northeast of the South China Sea	KU872171.1	Anti-*A. hydrophila* and *E. coli*; 4 and 8 μg/mL	Yan et al. (2023)
Neoechinulin A (**52**)	*Aspergillus* sp. WHUF0343	The root soil of mangroves, the Yalong Bay, Sanya, Hainan province, China	–	Anti-*H. pylori* Hp159; 16 μg/mL	Yu et al. (2022)
	*A. hiratsukae* SCSIO 7S2001	Marine gorgonian coral, the South China Sea	MN347034	Anti-*K. pneumoniae* and MRSA; 50 and 12.5 μg/mL	Chen X. Y. et al., 2022
Asperfumigatin (**53**)	*A. fumigatus* H22	Seawater, the Western Pacific	–	Anti-MRSA; 5 μM	Zhang R. et al. (2022)
Fumitremorgin B (**54**)	*A. fumigatus* H22	Seawater, the Western Pacific	–	Anti-MRSA; 20 μM	Zhang R. et al. (2022)
13-Oxofumitremorgin B (**55**)	*A. fumigatus* H22	Seawater, the Western Pacific	–	Anti-MRSA; 1.25 μM	Zhang R. et al. (2022)
Spirotryprostatin C (**56**)	*A. fumigatus* H22	Seawater, the Western Pacific	–	Anti-MRSA; 10 μM	Zhang R. et al. (2022)
(−)-Chaetominine (**57**)	*A. fumigatus* H22	Seawater, the Western Pacific	–	Anti-MRSA; 25 μM	Zhang R. et al. (2022)
Fumigaclavine C (**58**)	*A. fumigatus* H22	Seawater, the Western Pacific	–	Anti-MRSA; 12.5 μM	Zhang R. et al. (2022)
Epi-aszonalenin A (**59**)	*A. fumigatus* SCSIO 41012	Deep-sea sediment, the Indian Ocean	KM924435	Anti-*A. baumanii* ATCC 15122; 6.25 μg/mL	Limbadri et al. (2018)
3-((1-Hydroxy-3-(2-methylbut-3-en-2-yl)-2-oxoindolin-3-yl)methyl)-1-methyl-3,4-dih-ydrobenzo[e] [1,4]diazepine-2,5-dione (**60**)	Aspergillus sp.	Marine sponge, the Adriatic Sea	–	Anti-*V. harveyi* and *V. natriegens*; 1.0 μg/mL	Zhou et al. (2014)
Gliotoxin (**61**)	*Aspergillus* sp. SCSIO Ind09F01	Deep-sea sediment, the Indian Ocean	AY373869	Anti-*M. tuberculosis*; 0.030 μM	Luo et al. (2017)
*β*-Cyclopiazonic acid (**62**)	*A. felis* FM324	Beach soil, the Big Island, Hawaii	MZ227547	Anti-*S. aureus*, MRSA, and *B. subtilis*; 59.2 μM	Wang et al. (2021)
(2*R*,4b*R*,6a*S*,12b*S*,12c*S*,14a*S*)-4b-Deoxy-*β*-aflatrem (**63**)	*A. flavus* OUCMDZ-2205	Marine prawn, the Lianyungang Sea, Jiangsu province, China	KC120773	Anti-*S. aureus*; 20.5 μM	Sun et al. (2014)
Sclerotiamide K (**64**)	A. sclerotiorum LZDX-33-4	Marine gorgonian coral, the South China Sea	OK012383.1	Anti-*S. aureus* ATCC29213; 64 μM	Meng et al. (2022)
Sclerotiamide L (**65**)	A. sclerotiorum LZDX-33-4	Marine gorgonian coral, the South China Sea	OK012383.1	Anti-*S. aureus* ATCC29213; 4 μM	Meng et al. (2022)
Sclerotiamide M (**66**)	A. sclerotiorum LZDX-33-4	Marine gorgonian coral, the South China Sea	OK012383.1	Anti-*S. aureus* ATCC29213; 64 μM	Meng et al. (2022)
Sclerotiamide N (**67**)	A. sclerotiorum LZDX-33-4	Marine gorgonian coral, the South China Sea	OK012383.1	Anti-*S. aureus* ATCC29213; 64 μM	Meng et al. (2022)
Sclerotiamide O (**68**)	A. sclerotiorum LZDX-33-4	Marine gorgonian coral, the South China Sea	OK012383.1	Anti-*S. aureus* ATCC29213; 64 μM	Meng et al. (2022)
Sclerotiamide *p* (**69**)	A. sclerotiorum LZDX-33-4	Marine gorgonian coral, the South China Sea	OK012383.1	Anti-*S. aureus* ATCC29213; 32 μM	Meng et al. (2022)
Sclerotiamide Q (**70**)	A. sclerotiorum LZDX-33-4	Marine gorgonian coral, the South China Sea	OK012383.1	Anti-*S. aureus* ATCC29213; 64 μM	Meng et al. (2022)
Sclerotiamide R (**71**)	A. sclerotiorum LZDX-33-4	Marine gorgonian coral, the South China Sea	OK012383.1	Anti-*S. aureus* ATCC29213; 32 μM	Meng et al. (2022)
Fumigatoside E (**72**)	*A. fumigatus* SCSIO 41012	Deep-sea sediment, the Indian Ocean	KM924435	Anti-*A. baumanii* ATCC 19606, ATCC 15122, *S. aureus* ATCC 16339, and *K. pneumonia* ATCC 14578; 12.5, 6.25, 6.25, and 12.5 μg/mL	Limbadri et al. (2018)
Fumigatoside *F* (**73**)	*A. fumigatus* SCSIO 41012	Deep-sea sediment, the Indian Ocean	KM924435	Anti-*A. baumanii* ATCC 19606; 6.25 μg/mL	Limbadri et al. (2018)
Fumiquinazoline G (**74**)	*A. fumigatus* SCSIO 41012	Deep-sea sediment, the Indian Ocean	KM924435	Anti-*A. baumanii* ATCC 15122, *S. aureus* ATCC 16339, ATCC 29213, and *K. pneumonia* ATCC 14578; 6.25, 12.5, 12.5, and 25 μg/mL	Limbadri et al. (2018)
Cottoquinazoline H (**75**)	*A. versicolor* AS-212	Deep-sea coral, the Magellan Seamounts	OP009765.1	Anti-*E. coli*, *M. luteus*, *V. harveyi*, *V. parahaemolyticus*, *V. vulnificus*, Curvularia spicifera, and Colletotrichum gloeosporioides; 72.2, 36.1, 18.1, 9.0, 72.2, 72.2, and 72.2 μg/mL	Dong et al. (2023a)
Cottoquinazoline A (**76**)	*A. versicolor* AS-212	Deep-sea coral, the Magellan Seamounts	OP009765.1	Anti-*A. hydrophila*, *M. luteus*, *V. harveyi*, *V. parahaemolyticus*, *V. vulnificus*, C. spicifera, and C. gloeosporioides; 18.6, 74.6, 37.3, 37.3, 74.6, 74.6, and 74.6 μg/mL	Dong et al. (2023a)
	*A. versicolor* CF-09-9	Seawater, the Bohai Sea	–	Anti-*E. coli*; 5.0 μM	Zhang L. et al. (2020); Zhang Y. H. et al. (2020)
Aspergicin (**77**)	Aspergillus sp.	mangrove plant *Avicennia marina*, Zhangjiang, Guangdong province, China	–	Anti-*B. subtilis* and *B. dysenteriae*; 15.6 and 15.6 μg/mL	Zhu et al. (2011)
Brevianamide M (**78**)	*A. versicolor* pt20	Marine brown alga, the Pingtan Island, Fujian province, China	–	Weak (anti-*E. coli* and *S. aureus*); inhibitory diameters of 11 and 10 mm at 30 μg/disk	Miao et al. (2012)
Fumiquinazoline D (**79**)	*A. fumigatus* M580	Sea cucumber, the Co To-Thanh Island, Vietnam	MW015802	Anti-*E. faecalis* and *S. enterica*; 32 and 256 μg/mL	Tuan et al. (2022)
Fumiquinazoline C (**80**)	*A. fumigatus* M580	Sea cucumber, the Co To-Thanh Island, Vietnam	MW015802	Anti-*B. subtilis* and *B. dysenteriae*; 32 and 64 μg/mL	Tuan et al. (2022)
	*A. fumigatus* SCSIO 41012	Deep-sea sediment, the Indian Ocean	KM924435	Anti-*S. aureus* ATCC16339 and ATCC 29213; 1.56 and 0.78 μg/mL	Limbadri et al. (2018)
3-Hydroxy-6-methoxy-4-phenylquinolin-2(1*H*)-one (**81**)	*A. versicolor* AS-212	Deep-sea coral, the Magellan Seamounts	OP009765.1	Anti-*V. harveyi* and *V. alginolyticus*; 8.0 μg/mL	Dong et al. (2023b)
3-Methoxy-6-hydroxy-4-phenylquinolin-2(1*H*)-one (**82**)	*A. versicolor* AS-212	Deep-sea coral, the Magellan Seamounts	OP009765.1	Anti-*V. harveyi* and *V. alginolyticus*; 32 μg/mL	Dong et al. (2023b)
Cytochalasin Z17 (**83**)	Aspergillus sp.	Marine sponge, the Adriatic Sea	–	Anti-*R. litoralis*; 0.0001 μg/mL	Zhou et al. (2014)
Aspochalasin I (**84**)	*A. elegans* ZJ-2008010	Soft coral, the South China Sea	–	Anti-*S. epidermidis* and *S. aureus*; 20 and 10 μg/mL	Zheng et al. (2013)
Aspochalasin D (**85**)	*A. elegans* ZJ-2008010	Soft coral, the South China Sea	–	Anti-*S. epidermidis*, *S. aureus*, *E. coli*, and *B. cereus*; 10 μg/mL	Zheng et al. (2013)
Aspochalasin PZ (**86**)	*A. elegans* ZJ-2008010	Soft coral, the South China Sea	–	Anti-*S. epidermidis*; 20 μg/mL	Zheng et al. (2013)
Emestrins M (**87**)	*A. terreus* RA2905	Sea hare, the South China Sea	MK611650	Anti-*P. aeruginosa* ATCC 27853; 64 μg/mL	Wu et al. (2020a)
Emethacin C (**88**)	*A. terreus* RA2905	Sea hare, the South China Sea	MK611650	Anti-*P. aeruginosa* ATCC 27853; 32 μg/mL	Wu et al. (2020a)
4′-OMe-asperphenamate (**89**)	*A. elegans* ZJ-2008010	Soft coral, the South China Sea	–	Anti-*S. epidermidis*; 10 μg/mL	Zheng et al. (2013)
Asperphenamate (**90**)	*A. elegans* ZJ-2008010	Soft coral, the South China Sea	–	Anti-*S. epidermidis*; 10 μg/mL	Zheng et al. (2013)
Sclerotiotide M (**91**)	*A. insulicola* HDN151418	Marine sponge, the Prydz Bay, Antarctica	MT898544	Anti-*B. cereus*, *P. species*, *M. phlei*, *E. tarda*, *B. subtilis*, MRCNS, MRSA, and *V. parahemolyticus*; 3.13, 3.13, 3.13, 1.56, 6.25, 12.5, 25, and 3.13 μM	Sun et al. (2020)
Sclerotiotide N (**92**)	*A. insulicola* HDN151418	Marine sponge, the Prydz Bay, Antarctica	MT898544	Anti-*B. cereus*, *P. species*, *M. phlei*, *E. tarda*, *B. subtilis*, MRCNS, MRSA, and *V. parahemolyticus*; 6.25, 6.25, 12.5, 1.56, 12.5, 25, 25, and 6.25 μM	Sun et al. (2020)
Sclerotiotide O (**93**)	*A. insulicola* HDN151418	Marine sponge, the Prydz Bay, Antarctica	MT898544	Anti-*E. tarda*; 25.0 μM	Sun et al. (2020)
Sclerotiotide L (**94**)	*A. insulicola* HDN151418	Marine sponge, the Prydz Bay, Antarctica	MT898544	Anti-*B. cereus*, P. species, *E. tarda*, and V. parahemolyticus; 25.0 μM	Sun et al. (2020)
Sclerotiotide *F* (**95**)	*A. insulicola* HDN151418	Marine sponge, the Prydz Bay, Antarctica	MT898544	Anti-*B. cereus*, P. species, *E. tarda*, and V. parahemolyticus; 25.0 μM	Sun et al. (2020)
Aspertides D (**96**)	A. tamarii MA-21 and *A. insuetus* SD-512	Mangrove plant *Sonneratia paracaseolaris*, Wenchang, Hainan province, China and deep-sea sediment, the South China Sea	HQ891663 MN696202	Anti-*E. tarda*, *V. alginolyticus*, *V. anguillarum*, and *V. vulnificus*; 8.0, 16, 32, and 8.0 μg/mL	Chi et al. (2023)
Aspertides E (**97**)	A. tamarii MA-21 and *A. insuetus* SD-512	Mangrove plant *S. paracaseolaris*, Wenchang, Hainan province, China and deep-sea sediment, the South China Sea	HQ891663 MN696202	Anti-*E. tarda* and *S. aureus*; 16 and 8.0 μg/mL	Chi et al. (2023)
Unguisins A (**98**)	*A. nidulans* M256	Marine sponge *Echinodictyum conulosum*, the Bai Tu Long Sea, Quang Ninh province, Vietnam	OR166104.1	Anti-*E. faecalis*; 32 μg/mL	Thi et al. (2023)
Unguisins B (**99**)	*A. nidulans* M256	Marine sponge *E. conulosum*, the Bai Tu Long Sea, Quang Ninh province, Vietnam	OR166104.1	Anti-*E. faecalis*; 128 μg/mL	Thi et al. (2023)
Ochratoxin A methyl ester (**100**)	*A. elegans* KUFA0015	Marine sponge *Monanchora unguiculata*, the Kram Island, Thailand	KX431209	Anti-*E. faecalis* ATCC 29212, B3/101, and *S. aureus* ATCC29213; 16, 16, and 8 μg/mL	Kumla et al. (2021)
Aspergamide A (**101**)	Aspergillus sp. LS53	Marine sponge, Sanya, Hainan province, China	–	Anti-*V. harveyi*; 16 μg/mL	Zhang L. et al. (2020); Zhang Y. H. et al. (2020)
11-*O*-methylpseurotin A (**102**)	*A. fumigatus* H22	Seawater, the Western Pacific	–	Anti-MRSA; 10 μM	Zhang R. et al. (2022)
Azaspirofuran B (**103**)	*A. fumigatus* H22	Seawater, the Western Pacific	–	Anti-MRSA; 5 μM	Zhang R. et al. (2022)
Azaspirofuran A (**104**)	*A. fumigatus* H22	Seawater, the Western Pacific	–	Anti-MRSA; 5 μM	Zhang R. et al. (2022)
Dibetanide (**105**)	Aspergillus sp. LS57	Marine sponge, the Xisha islands, China	–	Anti-*B. cinerea*; 256 μg/mL	Li W. H. et al. (2023)
Ochratoxin B (**106**)	*A. elegans* KUFA0015	Marine sponge *Monanchora unguiculata* the Kram Island, Thailand		Anti-*S. aureus* 272,123; 50 μg/mL	Duraes et al. (2021)
Dihydroisoflavipucine (**107**)	Aspergillus sp.	Marine sponge *Tethya aurantium*, the Adriatic Sea	–	Anti-*S. aureus*, *S. putrefaciens*, and *V. natriegens*; 0.001 μg/mL	Zhou et al. (2014)
(+)-Asperfuranone (**108**)	*A. terreus* RA2905	Sea hare *Aplysia pulmonica*, the South China Sea	MK611650	Weak (anti-*P. aeruginosa*)	Wu et al. (2020b)
(−)-Asperfuranone (**109**)	*A. terreus* RA2905	Sea hare *A. pulmonica*, the South China Sea	MK611650	Anti-*P. aeruginosa*; 128 μg/mL	Wu et al. (2020b)
Carneusin B (**110**)	*A. carneus* GXIMD00519	Marine coral, the Weizhou Islands, Guangxi province, China	MT672623	Anti-*V. rotiferianus* and *A. macleodii*; 64 μg/mL	Lu et al. (2023)
Asperalin A (**111**)	*A. alabamensis* SYSU-6778	Mangrove plant *Enhalus acoroides*, the Dongzhai Port, Hainan province, China	MH863631.1	Anti-*S. aureus*, *S. iniae*, and *S. parauberis*; 21.8, 21.8, and 43.6 μM	Hu et al. (2023)
Asperalin B (**112**)	*A. alabamensis* SYSU-6778	Mangrove plant *E. acoroides*, the Dongzhai Port, Hainan province, China	MH863631.1	Anti-*S. aureus*, *S. iniae*, and *S. parauberis*; 21.8, 21.8, and 43.6 μM	Hu et al. (2023)
Asperalin C (**113**)	*A. alabamensis* SYSU-6778	Mangrove plant *E. acoroides*, the Dongzhai Port, Hainan province, China	MH863631.1	Anti-*S. aureus*, *S. iniae*, and *S. parauberis*; 10.1, 5.0, and 10.1 μM	Hu et al. (2023)
Asperalin D (**114**)	*A. alabamensis* SYSU-6778	Mangrove plant *E. acoroides*, the Dongzhai Port, Hainan province, China	MH863631.1	Anti-*S. aureus*, *S. iniae*, and *S. parauberis*; 10.1, 5.0, and 10.1 μM	Hu et al. (2023)
Asperalin E (**115**)	*A. alabamensis* SYSU-6778	Mangrove plant *E. acoroides*, the Dongzhai Port, Hainan province, China	MH863631.1	Anti-*S. iniae* and *S. parauberis*; 2.2 and 71.1 μM	Hu et al. (2023)
Asperalin *F* (**116**)	*A. alabamensis* SYSU-6778	Mangrove plant *E. acoroides*, the Dongzhai Port, Hainan province, China	MH863631.1	Anti-*S. aureus*, *S. iniae*, *S. parauberis*, *B. subtilis*, and *E. ictalurid*; 21.8, 43.6, 87.3, 21.8, and 10.9 μM	Hu et al. (2023)
*N*-(3-acetamidopropyl)-3,4- dihydroxybenzamide (**117**)	*A. alabamensis* SYSU-6778	Mangrove plant *E. acoroides*, the Dongzhai Port, Hainan province, China	MH863631.1	Anti-*E. ictalurid*; 79.3 μM	Hu et al. (2023)
Sclerotiamide I (**118**)	A. sclerotiorum LZDX-33-4.	Marine gorgonian coral, the South China Sea	OK012383.1	Anti-*S. aureus* ATCC29213; 16 μM	Meng et al. (2022)
Sclerotiamide J (**119**)	A. sclerotiorum LZDX-33-4.	Marine gorgonian coral, the South China Sea	OK012383.1	Anti-*S. aureus* ATCC29213; 16 μM	Meng et al. (2022)
Kipukasin H (**120**)	*A. versicolor*	Marine gorgonian *Dichotella* gemmacea, the Xisha Islands, the South China Sea	AY373880	Anti-*S. epidermidis*; 12.5 μg/mL	Chen et al. (2014)
Kipukasin I (**121**)	*A. versicolor*	Marine gorgonian *D.* gemmacea, the Xisha Islands, the South China Sea	AY373880	Anti-*S. epidermidis*; 12.5 μg/mL	Chen et al. (2014)
Kipukasin E (**122**)	*A. versicolor*	Marine gorgonian *D.* gemmacea, the Xisha Islands, the South China Sea	AY373880	Anti-*S. epidermidis*; 50.0 μg/mL	Chen et al. (2014)
Kipukasin D (**123**)	*A. versicolor*	Marine gorgonian *D.* gemmacea, the Xisha Islands, the South China Sea	AY373880	Anti-*S. epidermidis*; 50.0 μg/mL	Chen et al. (2014)
Perinadine B (**124**)	Aspergillus sp. LS116	Marine sponge, Linshui, Hainan province, China	FJ864703	Anti-*B. subtilis*; 32.0 μg/mL	Liu Y. et al. (2022)
Perinadine C (**125**)	Aspergillus sp. LS116	Marine sponge, Linshui, Hainan province, China	FJ864703	Anti-*B. subtilis*; 64.0 μg/mL	Liu Y. et al. (2022)
Neoaspergillic (**126**)	*Aspergillus* sp. CF07002	Marine sediment, the eastern Pacific Ocean off Panama	KM819008	Anti-*B. cereus*, *K. pneumoniae*, and *E. coli*; 30.0–40.0 μg/mL	Cardoso-Martinez et al. (2015)
Hydroxyneoaspergillic acid (**127**)	A. ochraceopetaliformis SCSIO 41018	Marine sponge	MH109740.1	Anti-MRSA, *S. aureus*, *E. faecalis*, *A. baumannii*, *E. coli*, and *K. pneumonia*; 7.8, 7.8, 0.9, 0.45, 62.5, and 7.8 μg/mL	Guo et al. (2021)
Dizinchydroxyneoaspergillin (**128**)	A. ochraceopetaliformis SCSIO 41018	Marine sponge	MH109740.1	Anti-MRSA, *S. aureus*, *E. faecalis*, *A. baumannii*, *E. coli*, and *K. pneumonia*; 3.9, 3.9, 0.9, 0.45, 125, and 3.9 μg/mL	Guo et al. (2021)
Puniceusine N (**129**)	*A. puniceus* SCSIO z021	Deep-sea sediment, Okinawa Trough	GU456970	Anti-*S. aureus*, MRSA and *E. coli*; 100 μg/mL	Liu C. M. et al. (2022)
Preussin (**130**)	*A. candidus* KUFA0062	Marine sponge, the coral reef at Similan Island National Park, Thailand	KX431210	Anti-*S. aureus* ATCC 29213, *E. faecalis* ATCC 29212, B3/101, and MRSA; 32, 32, 64, and 32 μg/mL	Buttachon et al. (2018)
6,6′-Oxybis(1,3,8-trihydroxy-2-((*S*)-1-methoxyhexyl) anthracene-9,10-dione) (**131**)	*A. versicolor* INF16-17	Marine clam, the East China Sea	–	Anti-*S. aureus*; 30 μg/mL	Li et al. (2019)
6,6′-Oxybis(1,3,8-trihydroxy-2-((*S*)-1-hydroxyhexyl) anthracene-9,10-dione) (**132**)	*A. versicolor* INF16-17	Marine clam, the East China Sea	–	Anti-*S. aureus*; 30 μg/mL	Li et al. (2019)
Xanthomegnin (**133**)	*A. elegans* KUFA0015	Marine sponge *Monanchora unguiculata* the Kram Island, Thailand	KX431209	Anti-*E. faecalis* ATCC 29212, *S. aureus* ATCC 29213, and MRSA; 32, 32, and 16 μg/mL	Kumla et al. (2021)
Viomellein (**134**)	*A. elegans* KUFA0015	Marine sponge *Monanchora unguiculata* the Kram Island, Thailand	KX431209	Anti-*E. faecalis* ATCC 29212, *S. aureus* ATCC 29213, and MRSA; 8, 8 and 2 μg/mL	Kumla et al. (2021)
Versiconol B (**135**)	Aspergillus sp. F40	Marine sponge, the sea area near Xuwen County, Guangdong province, China	KT164776	Anti-*S. aureus* and *V. parahaemolyticus*; 48 and 24 μg/mL	Tian et al. (2018)
Versiconol (**136**)	Aspergillus sp. F40	Marine sponge, the sea area near Xuwen County, Guangdong province, China	KT164776	Anti-*V. parahaemolyticus*; 12 μg/mL	Tian et al. (2018)
2-(Dimethoxymethyl)-1-hydro xyanthracene-9,10-dione (**137**)	*A. versicolor* 3A00029	Deep-sea sediment, the West Pacific Ocean	–	Anti-MRSA, *V. vulnificus*, *V. rotiferianus*, and *V. campbellii*; 3.9, 31.3, 62.5, and 15.6 μg/mL	Wang et al. (2018)
Damnacanthal (**138**)	*A. versicolor* 3A00029	Deep-sea sediment, the West Pacific Ocean	–	Anti-MRSA, *V. vulnificus*, *V. rotiferianus*, and *V. campbellii*; 62.5, 62.5, 62.5, and 125 μg/mL	Wang et al. (2018)
Xanthopurpurin (**139**)	*A. versicolor* 3A00029	Deep-sea sediment, the West Pacific Ocean	–	Anti-MRSA, *V. vulnificus*, *V. rotiferianus*, and *V. campbellii*; 62.5, 62.5, 125, and 62.5 μg/mL	Wang et al. (2018)
Isoversicolorin C (**140**)	*A. nidulans* MA-143	Mangrove plant Rhizophora stylosa	JQ839285	Anti-*E. coli*, *M. luteus*, *V. vulnificus*, *V. alginolyticus*, *E. ictaluri*, and *V. parahaemolyticus*; 32, 16, 64, 1, 4, and 32 μg/mL	Yang et al. (2018a)
Versicolorin C (**141**)	*A. nidulans* MA-143	Mangrove plant *R. stylosa*	JQ839285	Anti-*E. coli*, *M. luteus*, *V. anguillarum*, *V. alginolyticus*, *E. ictaluri*, and *V. parahaemolyticus*; 1, 32, 4, 16, 8, and 1 μg/mL	Yang et al. (2018a)
Emodin (**142**)	*A. fumigatus* MF029	Marine sponge *Hymeniacidon perleve*, the Bohai Sea	MH974808	Anti-MRSA, *S. aureus*, and BCG; 50, 50, and 1.25 μg/mL	Song Z. J. et al. (2021)
6,8-Di-*O*-methylaverufin (**143**)	*A. versicolor* pt20	Marine brown alga *Spiraea thunbergii*, the Pingtan Island, Fujian province, China	–	Anti-*E. coli* and *S. aureus*; Inhibitory diameters of 10 and 10 mm at 30 μg/disk	Miao et al. (2012)
6-*O*-methylaverufin (**144**)	*A. versicolor* pt20	Marine brown alga *S. thunbergii*, the Pingtan Island, Fujian province, China	–	Anti-*E. coli* and *S. aureus*; Inhibitory diameters of 10 and 10 mm at 30 μg/disk	Miao et al. (2012)
6,8-Di-*O*-methylaverantin (**145**)	*A. versicolor* EN-7	Marine brown alga *S. thunbergia*, the Qingdao coastline, Shandong province, China	EU042148	Weak (anti-*E. coli*); Inhibitory diameter of 7.0 mm at 20 μg/disk	Zhang et al. (2012)
6,8-Di-*O*-methylversiconol (**146**)	*A. versicolor* EN-7	Marine brown alga *S. thunbergia*, the Qingdao coastline, Shandong province, China	EU042148	Weak (anti-*E. coli*); Inhibitory diameter of 6.5 mm at 20 μg/disk	Zhang et al. (2012)
Averantin (**147**)	*A. versicolor* PF10M	Marine sponge, the Jeju Island, Korea	–	Anti-*S. pyogenes* 308A, 77A, and *S. aureus* SG511, 285, 503; 0.78, 3.13, 3.13, 3.13, and 1.56 μg/mL	Lee et al. (2010)
Averufin (**148**)	*A. versicolor* PF10M	Marine sponge, the Jeju Island, Korea	–	Anti-*S. pyogenes* 308A and *S. aureus* SG511, 285, 503; 6.25, 12.50, 12.50, and 6.25 μg/mL	Lee et al. (2010)
Nidurufin (**149**)	*A. versicolor* PF10M	Marine sponge, the Jeju Island, Korea	–	Anti-*S. pyogenes* 308A, 77A, and *S. aureus* SG511, 285, 503; 3.13, 6.25, 6.25, 3.13, 3.13, and 3.13 μg/mL	Lee et al. (2010)
6,8-Di-*O*-methylversicolorin A (**150**)	*Aspergillus* sp. WHUF05236	Deep-sea sediment	OM638737	Anti-*H. pylori* 26,695 and G27; 43.47 μM	Lv et al. (2022)
Asperpyrone A (**151**)	Aspergillus sp. DM94	The rhizosphere soil of mangrove plant *Bruguiera gymnorrhiza*	–	Anti-*H. pylori* G27 and Hp159; 4 μg/mL	Gou et al. (2020)
Aurasperone A (**152**)	Aspergillus sp. DM94	The rhizosphere soil of mangrove plant *B. gymnorrhiza*	–	Anti-*H. pylori* G27 and Hp159; 8 and 16 μg/mL	Gou et al. (2020)
Aurasperone *F* (**153**)	Aspergillus sp. DM94	The rhizosphere soil of mangrove plant *B. gymnorrhiza*	–	Anti-*H. pylori* G27 and Hp159; 4 μg/mL	Gou et al. (2020)
Aurasperone B (**154**)	Aspergillus sp. DM94	The rhizosphere soil of mangrove plant *B. gymnorrhiza*	–	Anti-*H. pylori* G27 and Hp159; 8 and 16 μg/mL	Gou et al. (2020)
Fonsecinone A (**155**)	Aspergillus sp. DM94	the rhizosphere soil of mangrove plant *B. gymnorrhiza*	–	Anti-*H. pylori*; 16 μg/mL	Gou et al. (2020)
Asperpyrones C (**156**)	Aspergillus sp. DM94	the rhizosphere soil of mangrove plant *B. gymnorrhiza*	–	Anti-*H. pylori*; 16 μg/mL	Gou et al. (2020)
	A. welwitschiae CUGBMF180262	mud sample, the Xinglin Bay, XiaMen, China	MT120310	Anti-*H. pylori* G27 and Hp159; 4 μg/mL	Han et al. (2022)
Aspergixanthone I (**157**)	Aspergillus sp. ZA-01	Sediment, the Bohai Sea	–	Anti-V. parahemolyticus, *V. anguillarum*, and *V. alginolyticus*; 1.56, 1.56, and 3.12 μM	Zhu et al. (2018)
Aspergixanthone J (**158**)	Aspergillus sp. ZA-01	Sediment, the Bohai Sea	–	Anti-V. parahemolyticus, *V. anguillarum*, and *V. alginolyticus*; 6.25, 25.0, and 25.0 μM	Zhu et al. (2018)
Aspergixanthone K (**159**)	Aspergillus sp. ZA-01	Sediment, the Bohai Sea	–	Anti-V. parahemolyticus, *V. anguillarum*, and *V. alginolyticus*; 3.12, 25.0, and 12.5 μM	Zhu et al. (2018)
Aspergixanthone A (**160**)	Aspergillus sp. ZA-01	Sediment, the Bohai Sea	–	Anti-V. parahemolyticus, *V. anguillarum*, and *V. alginolyticus*; 25.0 μM	Zhu et al. (2018)
15-Acetyl tajixanthone hydrate (**161**)	Aspergillus sp. ZA-01	Sediment, the Bohai Sea	–	Anti-V. parahemolyticus, *V. anguillarum*, and *V. alginolyticus*; 12.5, 25.0, and 12.5 μM	Zhu et al. (2018)
Tajixanthone hydrate (**162**)	Aspergillus sp. ZA-01	Sediment, the Bohai Sea	–	Anti-V. parahemolyticus, *V. anguillarum*, and *V. alginolyticus*; 6.25, 6.25, and 12.5 μM	Zhu et al. (2018)
16-Chlorotajixanthone (**163**)	Aspergillus sp. ZA-01	Sediment, the Bohai Sea	–	Anti-V. parahemolyticus, *V. anguillarum*, and *V. alginolyticus*; 25.0, 6.25, and 25.0 μM	Zhu et al. (2018)
Secalonic acid D (**164**)	A. aculeatinus WHUF0198	Deep-sea sediment, the South China Sea	–	*H. pylori* G27, 26,695, 129, 159, *S. aureus* USA300, and *B. subtilis* 168; 4.0, 4.0, 2.0, 2.0, 2.0, and 1.0 μg/mL	Wu et al. (2023)
5-Epi-asperdichrome (**165**)	*A. versicolor* HDN1009	Mangrove soil, Guangzhou, China	KP765236	Anti-V. parahemolyticus, *B. subtilis*, *M. phlei*, and *P. aeruginosa*; 100, 200, 200, and 100 μg/mL	Yu et al. (2018)
Aflaxanthone A (**166**)	*A. flavus* QQYZ	Mangrove plant *Kandelia candel*, Huizhou, Guangdong province, China	JQ776536.1	Anti-MRSA and *B. subtilis*; 12.5 and 25 μg/mL	Zang et al. (2022)
Aflaxanthone B (**167**)	*A. flavus* QQYZ	Mangrove plant *K. candel*, Huizhou, Guangdong province, China	JQ776536.1	Anti-*B. subtilis*; 25 μg/mL	Zang et al. (2022)
5-Methoxydihy- drosterigmatocystin (**168**)	*A. versicolor* MF359	Marine sponge *H. perleve*, the Bohai Sea	HQ000003	Anti-*B. subtilis* and *S. aureus*; 3.125 and 12.5 μg/mL	Song et al. (2014)
Oxisterigmatocystin C (**169**)	Aspergillus sp. F40	Marine sponge, the sea area near Xuwen County, Guangdong province, China	KT164776	Anti-*S. aureus*; 48 μg/mL	Tian et al. (2018)
Sterigmatocystin (**170**)	A. sydowii DC08	Marine sponge, the Mandeh, South Coast, West Sumatra, Indonesia island	–	Anti-MRSA, MDPRA, *P. aeruginosa* ATCC 27853, *S. aureus* ATCC 25923, and *E. coli* ATCC 25922; 64, 128, 32, 32, and 16 μg/mL	Handayani et al. (2022)
2-Hydroxy-6-formyl-vertixanthone (**171**)	*A. sydowii* C1-S01-A7	Seawater, the West Pacific Ocean	MH571963	Anti-MRSA and CGMCC 1.12409; 16.3 and 16.1 μg/mL	Wang et al. (2019)
12-*O*-acetyl-sydowinin A (**172**)	*A. sydowii* C1-S01-A7	Seawater, the West Pacific Ocean	MH571963	Anti-MRSA and CGMCC 1.12409; 32.6 and 31.8 μg/mL	Wang et al. (2019)
Aspergillusone A (**173**)	*A. sydowii* C1-S01-A7	Seawater, the West Pacific Ocean	MH571963	Anti-MRSA and CGMCC 1.12409; 32.2 and 32.4 μg/mL	Wang et al. (2019)
AGI-B4 (**174**)	A. sydowii C1-S01-A7	Seawater, the West Pacific Ocean	MH571963	Anti-*V. vulnificus* MCCC E1758, MRSA, and CGMCC 1.12409; 32.5, 32.9 and 16.3 μg/mL	Wang et al. (2019)
Isosecosterigmatocystin (**175**)	*A. nidulans* MA-143	Mangrove plant *R. stylosa*	JQ839285	Anti-*E. ictaluri*; 16 μg/mL	Yang et al. (2018a)
*Seco-*penicitrinol A (**176**)	A. sydowii EN-534 and P. citrinum EN-535	Marine red alga *Laurencia okamurai*, Qingdao, Shandong province, China	MG242135 MG242136	Anti-*E. ictaluri* and *V. alginolyticus*; 64 and 32 μg/mL	Yang et al. (2018b)
Secalonic acid F1 (**177**)	A. brunneoviolaceus MF180246	Mangrove mud sample, the Xinglin Bay, Xiamen, China	–	Anti-*S. aureus*; 25 μg/mL	Xu et al. (2024)
Secalonic acid H (**178**)	A. brunneoviolaceus MF180246	Mangrove mud sample, the Xinglin Bay, Xiamen, China	–	Anti-*S. aureus*; 50 μg/mL	Xu et al. (2024)
Penicillixanthone A (**179**)	A. brunneoviolaceus MF180246	Mangrove mud sample, the Xinglin Bay, Xiamen, China	–	Anti-*S. aureus*; 6.25 μg/mL	Xu et al. (2024)
Chrysoxanthone C (**180**)	A. brunneoviolaceus MF180246	Mangrove mud sample, the Xinglin Bay, Xiamen, China	–	Anti-*S. aureus*; 50 μg/mL	Xu et al. (2024)
Aspergetherin A (**181**)	*A. terreus* 164,018	Marine sponge, the South China Sea	–	Anti-MRSA 05–72 and USA300; 128 μg/mL	Li J. X. et al. (2023)
Vioxanthin (**182**)	*A. elegans* KUFA0015	Marine sponge *Monanchora unguiculata* the Kram Island, Thailand	KX431209	Anti-*E. faecalis* ATCC29212, VRE, *S. aureus* ATCC 29213, and MRSA; 2, 1, 2, and 0.5 μg/mL	Kumla et al. (2021)
Aspulvinone B′ (**183**)	*A. flavipes* KUFA1152	Marine sponge *Mycale* sp., the Samaesan Island, Thailand	MT814286	Anti-*E. faecalis* ATCC29212, VRE, *S. aureus* ATCC 29213, and MRSA;32, 32, 16, and 16 μg/mL	Machado et al. (2021)
Aspulvinone H (**184**)	*A. flavipes* KUFA1152	Marine sponge *Mycale* sp., the Samaesan Island, Thailand	MT814286	Anti-*E. faecalis* ATCC29212, VRE, *S. aureus* ATCC 29213, and MRSA; 32, 64, 16 and 16 μg/mL	Machado et al. (2021)
Aspulvinone R (**185**)	*A. flavipes* KUFA1152	Marine sponge *Mycale* sp., the Samaesan Island, Thailand	MT814286	Anti-*E. faecalis* ATCC29212, VRE, *S. aureus* ATCC 29213, and MRSA; 8, 16, 8 and 16 μg/mL	Machado et al. (2021)
Aspulvinone S (**186**)	*A. flavipes* KUFA1152	Marine sponge *Mycale* sp., the Samaesan Island, Thailand	MT814286	Anti-*E. faecalis* ATCC29212, VRE, *S. aureus* ATCC 29213, and MRSA; 8, 8, 4, and 16 μg/mL	Machado et al. (2021)
Asperteretal E (**187**)	*A. terreus* SCSIO FZQ028	Deep-sea sediment, the South China	KX792117	Weak (anti-*S. aureus*, *B. thuringiensis*, *B. subtilis*, and *E. coli*); Inhibitory diameters of 8.94, 9.77, 7.98, and 7.53 mm at 30 μg/disk	Zeng et al. (2020b)
Aspernolide A (**188**)	*A. terreus* SCSIO FZQ028	Deep-sea sediment, the South China	KX792117	Weak (anti-*S. aureus*, *B. thuringiensis*, *B. subtilis*, and *E. coli*); Inhibitory diameters of 8.16, 9.13, 7.49, and 7.64 mm at 30 μg/disk	Zeng et al. (2020b)
Butyrolactone I (**189**)	*Aspergillus* sp. SCSIO 41029	Deep-sea sediment, the South China	MH591418.1	Anti-*S. aureus*; 0.78 μg/mL	Chen et al. (2021)
Asperbutenolide D (**190**)	*A. terreus* SCAU011	The rhizosphere sediment of a mangrove plant *R. stylosa*, the Techeng Isle, China	KY827341	Anti-*S. aureus*; 21.3 μM	Bao et al. (2021)
(+)-3′,3′-Di-(dimethylallyl)- butyrolactone II (**191**)	*A. terreus* SCAU011	The rhizosphere sediment of a mangrove plant *R. stylosa*, the Techeng Isle, China	KY827341	Anti-*S. aureus*; 17.4 μM	Bao et al. (2021)
Aspernolide E (**192**)	*A. terreus* SCAU011	The rhizosphere sediment of a mangrove plant *R. stylosa*, the Techeng Isle, China	KY827341	Anti-*S. aureus*; 26.1 μM	Bao et al. (2021)
Flavipesin A (**193**)	*A. flavipes* AIL8	Mangrove plant Acanthus ilicifolius, the Daya Bay, Shenzhen, China	–	Anti-*S. aureus* and *B. subtillis*; 8.0 and 0.25 μg/mL	Bai et al. (2014)
Versicolactone B (**194**)	*A. terreus* SCSIO41404	Marine soft coral *Sinularia* sp., the Sanya Bay, the South China Sea	KU866665.1	Anti-*E. faecalis*; 25 μg/mL	Peng et al. (2022)
Butyrolactone VI (**195**)	*A. terreus* SCSIO41404	Marine soft coral *Sinularia* sp., the Sanya Bay, the South China Sea	KU866665.1	Anti-*K. pneumoniae*; 50 μg/mL	Peng et al. (2022)
Asperbutenolide A (**196**)	*A. terreus* SCAU011	the rhizosphere soil of mangrove plant R. stylosa, the Techeng Isle, China	–	Anti-*S. aureus* and *V. splendidus*; 1.30 and 3.70 μg/mL	Bao et al. (2020)
5*R*-(+)-9-Hydroxy- microperfuranone (**197**)	Aspergillus sp. ZZ1861	Sea mud, the coastal area of Putuo, Zhoushan, China	OR985107	Anti-*E. coli*; 50 μg/mL	Ha et al. (2024)
5*R*-(+)-Microperfuranone (**198**)	*Aspergillus* sp. ZZ1861	Sea mud, the coastal area of Putuo, Zhoushan, China	OR985107	Anti-*E. coli*; 25 μg/mL	Ha et al. (2024)
Asperpyranone A (**199**)	*A. terreus* RA2905	Sea hare *A. pulmonica*, the South China Sea	MK611650	Anti-*P. aeruginosa*; 32 μg/mL	Wu et al. (2020b)
Asperpyranone B (**200**)	*A. terreus* RA2905	Sea hare *A. pulmonica*, the South China Sea	MK611650	Anti-*P. aeruginosa*; 128 μg/mL	Wu et al. (2020b)
Nectriapyrone (**201**)	Aspergillus sp. LS53	Marine sponge *Haliclona* sp., Sanya, Hainan province, China	–	Anti-*V. harveyi*; 64 μg/mL	Zhang L. et al. (2020); Zhang Y. H. et al. (2020)
Asperisocoumarin A (**202**)	Aspergillus sp. LS53	Marine sponge *Haliclona* sp., Sanya, Hainan province, China	–	Anti-*V. harveyi*; 32 μg/mL	Zhang L. et al. (2020); Zhang Y. H. et al. (2020)
Unguinol (**203**)	A. unguis WR8	Marine sponge *Haliclona fascigera*, the Mandeh Island, South Coast of West Sumatera, Indonesia	MN273740	Anti-*E. coli*, *P. aeruginosa*, *S. aureus*, *E. faecalis*, *B. subtilis*, MRSA, *S. typosa*, *V. cholerae*, and *M. luteus*; 1.56, 3.12, 3.12, 3.12, 0.78, 3.12, 3.12, 0.78, and 0.78 μg/disk	Handayani et al. (2020)
	*A. unguis* PSU-MF16	Marine sponge *Dysidea* sp., the Koh Bulon Mai Pai, Satun Province, Thailand	KY397987	Anti-*S. aureus*; 128 μg/mL	Saetang et al. (2021)
2-Chlorounguinol (**204**)	A. unguis WR8	Marine sponge *H. fascigera*, the Mandeh Island, South Coast of West Sumatera, Indonesia	MN273740	Anti-*E. coli*, *P. aeruginosa*, *S. aureus*, *E. faecalis*, *B. subtilis*, MRSA, *S. typosa*, *V. cholerae*, and *M. luteus*; 1.56, 1.56,1.56, 0.78, 0.78, 0.78, 1.56, 0.78, and 0.78 μg/dis	Handayani et al. (2020)
	*A. unguis* PSU-MF16	Marine sponge *Dysidea* sp., the Koh Bulon Mai Pai, Satun Province, Thailand	KY397987	Anti-*S. aureus* and MRSA; 8 μg/mL	Saetang et al. (2021)
Nidulin (**205**)	A. unguis WR8	Marine sponge *H. fascigera*, the Mandeh Island, South Coast of West Sumatera, Indonesia	MN273740	Anti-*E. coli*, *P. aeruginosa*, *S. aureus*, *E. faecalis*, *B. subtilis*, MRSA, *S. typosa*, *V. cholerae*, and *M. luteus*; 0.78, 1.56, 0.78, 0.78, 0.78, 0.78, 1.56, 0.78, and 0.78 μg/disk	Handayani et al. (2020)
Aspergillusidone H (**206**)	*A. unguis* GXIMD 02505	Marine coral *Pocillopora damicornis*, the Weizhou Islands, Guangxi, China	OL989238	Weak (anti-MRSA)	Zhang Y. T. et al. (2022)
Nornidulin (**207**)	*A. unguis* GXIMD 02505	Marine coral *P. damicornis*, the Weizhou Islands, Guangxi, China	OL989238	Anti-MRSA, *M. variabilis*, and *M. jannaschii*; 2, 8, and 16 μg/mL	Zhang Y. T. et al. (2022)
	*A. unguis* PSU-MF16	Marine sponge *Dysidea* sp., the Koh Bulon Mai Pai, Satun Province, Thailand	KY397987	Anti-*S. aureus* and MRSA; 2 μg/mL	Saetang et al. (2021)
Aspergillusidone B (**208**)	*A. unguis* GXIMD 02505	Marine coral *P. damicornis*, the Weizhou Islands, Guangxi, China	OL989238	*M. variabilis*; 128 μg/mL	Zhang Y. T. et al. (2022)
Aspergillusidone C (**209**)	*A. unguis* GXIMD 02505	Marine coral *P. damicornis*, the Weizhou Islands, Guangxi, China	OL989238	Anti-MRSA, *M. variabilis*, and *M. jannaschii*; 32, 8 and 32 μg/mL	Zhang Y. T. et al. (2022)
	*A. unguis* PSU-MF16	Marine sponge *Dysidea* sp., the Koh Bulon Mai Pai, Satun Province, Thailand	KY397987	Anti-*S. aureus* and MRSA; 2 and 1 μg/mL	Saetang et al. (2021)
7-Dechloronidulin (**210**)	*A. nidulans* M256	Marine sponge *E. conulosum*, the Bai Tu Long Sea, Quang Ninh province, Vietnam	OR166104.1	Anti-*B. cereus*, *E. faecalis*, and *S. aureus*; 2, 4 and 4 μg/mL	Thi et al. (2023)
2,4-Dichlorounguinol (**211**)	*A. nidulans* M256	Marine sponge *E. conulosum*, the Bai Tu Long Sea, Quang Ninh province, Vietnam	OR166104.1	Anti-*B. cereus*, *E. faecalis*, *S. aureus*, *E. coli*, *P. aeruginosa*, and *S. enterica*; 16, 32, 32, 16, 64 and 32 μg/mL	Thi et al. (2023)
Emeguisin B (**212**)	*A. nidulans* M256	Marine sponge *E. conulosum*, the Bai Tu Long Sea, Quang Ninh province, Vietnam	OR166104.1	Anti-*E. faecalis* and *S. aureus*; 256 and 128 μg/mL	Thi et al. (2023)
Asperunguissidone A (**213**)	*A. unguis* PSU-MF16	Marine sponge *Dysidea* sp., the Koh Bulon Mai Pai, Satun Province, Thailand	KY397987	Anti-*S. aureus* and MRSA; 64 μg/mL	Saetang et al. (2021)
Asperunguislide A (**214**)	*A. unguis* PSU-MF16	Marine sponge *Dysidea* sp., the Koh Bulon Mai Pai, Satun Province, Thailand	KY397987	Anti-*M. gypseum*; 200 μg/mL	Saetang et al. (2021)
Asperlide (**215**)	*A. unguis* PSU-MF16	Marine sponge *Dysidea* sp., the Koh Bulon Mai Pai, Satun Province, Thailand	KY397987	Anti-*S. aureus* and MRSA; 200 μg/mL	Saetang et al. (2021)
Aspergiside C (**216**)	*A. unguis* PSU-MF16	Marine sponge *Dysidea* sp., the Koh Bulon Mai Pai, Satun Province, Thailand	KY397987	Anti-*S. aureus* and MRSA; 200 μg/mL	Saetang et al. (2021)
(3*S*)-3-Ethyl-5,7-dihydroxy-3,6-Dimethyl- phthalide (**217**)	*A. unguis* PSU-MF16	Marine sponge *Dysidea* sp., the Koh Bulon Mai Pai, Satun Province, Thailand	KY397987	Anti-*S. aureus* and MRSA; 2 and 4 μg/mL	Saetang et al. (2021)
Aspergisidone (**218**)	*A. unguis* PSU-MF16	Marine sponge *Dysidea* sp., the Koh Bulon Mai Pai, Satun Province, Thailand	KY397987	Anti-*S. aureus* and MRSA; 32 and 64 μg/mL	Saetang et al. (2021)
Folipastatin (**219**)	*A. unguis* PSU-MF16	Marine sponge *Dysidea* sp., the Koh Bulon Mai Pai, Satun Province, Thailand	KY397987	Anti-*S. aureus* and MRSA; 2 and 1 μg/mL	Saetang et al. (2021)
Emeguisins A (**220**)	*A. unguis* PSU-MF16	Marine sponge *Dysidea* sp., the Koh Bulon Mai Pai, Satun Province, Thailand	KY397987	Anti-*S. aureus* and MRSA; 0.5 μg/mL	Saetang et al. (2021)
8-Demethoxy-10-methoxy- wentiquinone C (**221**)	A. sydowii C1-S01-A7	Seawater, the West Pacific Ocean	MH571963	Anti-MRSA; 32.4 μg/mL	Wang et al. (2019)
Farnesylemefuranone D (**222**)	*A. insuetus* SD-512	Cold-seep sediment, the northeast of the South China Sea	MN650839	Anti-A. hydrophilia, *E. coli*, *E. tarda*, *P. aeruginosa*, *V. alginolyticus*, V. parahemolyticus, and *V. vulnificus*; 8.0, 32, 8.0, 16, 4.0, 16, and 4.0 μg/mL	Chi et al. (2020)
Farnesylemefuranone E (**223**)	*A. insuetus* SD-512	Cold-seep sediment, the northeast of the South China Sea	MN650839	Anti-A. hydrophilia, *E. coli*, *E. tarda*, *P. aeruginosa*, *V. alginolyticus*, V. parahemolyticus, and *V. vulnificus*; 16, 32, 8.0, 16, 8.0, 16, and 4.0 μg/mL	Chi et al. (2020)
Farnesylemefuranone *F* (**224**)	*A. insuetus* SD-512	Cold-seep sediment, the northeast of the South China Sea	MN650839	Anti-A. hydrophilia, *E. coli*, *E. tarda*, *P. aeruginosa*, *V. alginolyticus*, V. parahemolyticus, and *V. vulnificus*; 8.0, 32, 4.0, 8.0, 4.0, 8.0, and 4.0 μg/mL	Chi et al. (2020)
Silvaticol (**225**)	*Aspergillus* sp. ZZ1861	Sea mud sample, the Zhoushan Island, Zhejiang province, China	OR985107	Anti-*E. coli*; 12.5 μg/mL	Ha et al. (2024)
Aspergillumarin A (**226**)	Aspergillus sp.	Mangrove plant *B. gymnorrhiza*, the South China Sea coast	–	Anti-*S. aureus* and *B. subtilis*; 50 μg/mL	Li et al. (2012)
Aspergillumarin B (**227**)	Aspergillus sp.	Mangrove plant *B. gymnorrhiza*, the South China Sea coast	–	Anti-*S. aureus* and *B. subtilis*; 50 μg/mL	Li et al. (2012)
Aspergimarin G (**228**)	Aspergillus sp. NBUF87.	Marine sponge *Hymeniacidon* sp., the Xisha Islands, the South China Sea	–	Anti-*S. aureus* and *S. enteritidis*; 16 and 64 μg/mL	Lin S. X. et al. (2023)
(*R*)-3-Hydroxymellein (**229**)	*Aspergillus* sp. SCSIO41405	Marine coral, Sanya Bay, the South China Sea	–	Anti-MRSA; 100 μg/mL	Peng et al. (2021)
(3*R*,4*S*)-Trans-4-hydroxymellein (**230**)	*Aspergillus* sp. SCSIO41405	Marine coral, Sanya Bay, the South China Sea	–	Anti-*E. faecalis*; 100 μg/mL	Peng et al. (2021)
Nipyrone A (**231**)	*A. niger* LS24	Marine sponge *Haliclona* sp., Linshui, Hainan province, China	KX290301	Anti-*S. aureus*, *E. coli*, *B. subtilis*, MRSA, and *M. tuberculosis*; 64, 32, 64, 128 and 128 μg/mL	Ding et al. (2019)
Nipyrone B (**232**)	*A. niger* LS24	Marine sponge *Haliclona* sp., Linshui, Hainan province, China	KX290301	Anti-*S. aureus*, *E. coli*, *B. subtilis*, MRSA, and *M. tuberculosis*; 64, 64, 64, 128, and 128 μg/mL	Ding et al. (2019)
Nipyrone C (**233**)	*A. niger* LS24	Marine sponge *Haliclona* sp., Linshui, Hainan province, China	KX290301	Anti-*S. aureus*, *E. coli*, *B. subtilis*, MRSA, and *M. tuberculosis*; 8, 64, 16, 128, and 64 μg/mL	Ding et al. (2019)
Germicidin C (**234**)	*A. niger* LS24	Marine sponge *Haliclona* sp., Linshui, Hainan province, China	KX290301	Anti-*S. aureus*, *E. coli*, *B. subtilis*, MRSA, and *M. tuberculosis*; 64, 64, 32, 128, and 128 μg/mL	Ding et al. (2019)
Sartorypyrone A (**235**)	*Aspergillus* sp. WHUF03110	Mangrove soil sample, the Yalong Bay, Sanya, Hainan province, China	MZ661122	Anti-*B. subtilis*, *S. aureus*, and *H. pylori*; 1–8 μg/mL	Lv et al. (2021)
Asperochrin A (**236**)	*A. ochraceus* MA-15	The rhizospheric soil of mangrove plant *B. gymnorrhiza*, Hainan province, China	KP279929	Anti-A. hydrophilia, *V. anguillarum*, and V. harvevi; 8, 16 and 8 μg/mL	Liu et al. (2015)
Chlorohydroaspyrone A (**237**)	*A. ochraceus* MA-15	The rhizospheric soil of mangrove plant *B. gymnorrhiza*, Hainan province, China	KP279929	Anti-A. hydrophilia, *V. anguillarum*, and V. harvevi; 16, 32 and 16 μg/mL	Liu et al. (2015)
Chlorohydroaspyrone B (**238**)	*A. ochraceus* MA-15	The rhizospheric soil of mangrove plant *B. gymnorrhiza*, Hainan province, China	KP279929	Anti-A. hydrophilia, *V. anguillarum*, and V. harvevi;16, 32 and 32 μg/mL	Liu et al. (2015)
∆^2′^-1’-Dehydropenicillide (**239**)	*Aspergillus* sp. IMCASMF180035	A mud sample, the intertidal zones of the Yellow Sea, Qingdao, Shandong province, China	MW015145	Anti-*H. pylori*; 21.73 μM	Song F. H. et al. (2021)
Dehydropenicillide (**240**)	*Aspergillus* sp. IMCASMF180035	A mud sample, the intertidal zones of the Yellow Sea, Shandong province, China	MW015145	Anti-*H. pylori*; 21.61 μM	Song F. H. et al. (2021)
Aspergiloxathene A (**241**)	*Aspergillus* sp. IMCASMF180035	A mud sample, the intertidal zones of the Yellow Sea, Qingdao, Shandong province, China	MW015145	Anti-*S. aureus* and MRSA; 5.60 and 22.40 μM	Song F. H. et al. (2021)
Cowabenzophenone A (**242**)	*A. terreus*	Mangrove plant *B. gymnorrhyza*, Jaffna lagoon, Northern Province, Sri Lanka	–	Anti-*B. subtilis* and *S. aureus*; 1.0 and 2.0 μg/mL	Ukwatta et al. (2020)
Penicitrinone A (**243**)	A. sydowii EN-534 and P. citrinum EN-535	Marine red alga *L. okamurai*, Qingdao, Shandong province, China	MG242135 MG242136	Anti-*E. coli*, *V. parahaemolyticus*, *V. alginolyticus*, *M. luteus*, and *E. ictaluri*; 64, 16, 32, 16, and 32 μg/mL	Yang et al. (2018b)
Penicitrinone *F* (**244**)	A. sydowii EN-534 and P. citrinum EN-535	Marine red alga *L. okamurai*, Qingdao, Shandong province, China	MG242135 MG242136	Anti-*E. ictaluri*, *V. alginolyticus*, and *V. parahaemolyticus*; 64, 64, and 32 μg/mL	Yang et al. (2018b)
Citrinin (**245**)	A. sydowii EN-534 and P. citrinum EN-535	Marine red alga *L. okamurai*, Qingdao, Shandong province, China	MG242135 MG242136	Anti-*E. coli*, *V. alginolyticus*, *V. parahaemolyticus*, *M. luteus*, and *E. ictaluri*; 8, 16, 8, 16, and 32 μg/mL	Yang et al. (2018b)
25*S*-*O*-methylarugosin A (**246**)	*Aspergillus* sp. ZZ1861	Sea mud sample, the Zhoushan Island, Zhejiang province, China	OR985107	Weak (anti-MRSA)	Ha et al. (2024)
25*R*-*O*-methylarugosin A (**247**)	*Aspergillus* sp. ZZ1861	Sea mud sample, the Zhoushan Island, Zhejiang province, China	OR985107	Anti-MRSA; 50 μg/mL	Ha et al. (2024)
12*S*-Aspertetranone D (**248**)	*Aspergillus* sp. SY2601	Marine sediment, the Mariana Trench	OR646740	Anti-MRSA and *E. coli*; 3.75 and 5 μg/mL	Sun et al. (2024)
(10*S*,12*S*)-Chevalierone (**249**)	*A. chevalieri* HP-5	Mud sample, the coast of Shenzhen Bay, China	–	Anti-*P. aeruginosa* Inhibition rate 38.2% at the concentration of 200 μM	Wang Q. Y. et al. (2022)
(10*S*,12*R*)-Chevalierone (**250**)	*A. chevalieri* HP-5	Mud sample, the coast of Shenzhen Bay, China	–	Anti-*P. aeruginosa* and MRSA; Inhibition rate 81.9 and 74.1% at the concentration of 200 μM	Wang Q. Y. et al. (2022)
(10*R*,12*S*)-Chevalierone (**251**)	*A. chevalieri* HP-5	Mud sample, the coast of Shenzhen Bay, China	–	Anti-*P. aeruginosa* and MRSA; Inhibition rate 81.0 and 85.0% at the concentration of 200 μM	Wang Q. Y. et al. (2022)
(10*R*,12*R*)-Chevalierone (**252**)	*A. chevalieri* HP-5	Mud sample, the coast of Shenzhen Bay, China	–	Anti-*P. aeruginosa* and MRSA; Inhibition rate 91.5 and 88.5% at the concentration of 200 μM	Wang Q. Y. et al. (2022)
Asperphenone A (**253**)	Aspergillus sp. YHZ-1	Unidentified mangrove plant, Hainan province, China	–	Anti-*S. aureus*, *B. subtilis*, *S. pyogenes*, and *M. luteus*; 64.0, 64.0, 64.0, and 32.0 μg/mL	Guo et al. (2018)
Asperphenone B (**254**)	Aspergillus sp. YHZ-1	Unidentified mangrove plant, Hainan province, China	–	Anti-*S. aureus*, *B. subtilis*, *S. pyogenes*, and *M. luteus*; 32.0, 64.0, 32.0, and 32.0 μg/mL	Guo et al. (2018)
Penibenzophenone E (**255**)	*A. fumigatus* H22	Seawater, the Western Pacific	–	Anti-MRSA; 1.25 μM	Zhang R. et al. (2022)
Sulochrin (**256**)	*A. fumigatus* H22	Seawater, the Western Pacific	–	Anti-MRSA; 1.25 μM	Zhang R. et al. (2022)
Aspergiside A (**257**)	*A. unguis* PSU-MF16	Marine sponge *Dysidea* sp., the Koh Bulon Mai Pai, Satun Province, Thailand	KY397987	Anti-*S. aureus* and MRSA; 8 μg/mL	Saetang et al. (2021)
Aspergiside B (**258**)	*A. unguis* PSU-MF16	Marine sponge *Dysidea* sp., the Koh Bulon Mai Pai, Satun Province, Thailand	KY397987	Anti-*S. aureus* and MRSA; 128 μg/mL	Saetang et al. (2021)
Agonodepside A (**259**)	*A. unguis* PSU-MF16	Marine sponge *Dysidea* sp., the Koh Bulon Mai Pai, Satun Province, Thailand	KY397987	Anti-*S. aureus* and MRSA; 2 μg/mL	Saetang et al. (2021)
Agonodepside B (**260**)	*A. unguis* PSU-MF16	Marine sponge *Dysidea* sp., the Koh Bulon Mai Pai, Satun Province, Thailand	KY397987	Anti-*S. aureus* and MRSA; 8 and 16 μg/mL	Saetang et al. (2021)
Guisinol (**261**)	*A. unguis* GXIMD 02505	Marine coral *P. damicornis*, the Weizhou Islands, Guangxi, China	OL989238	Anti-MRSA and *M.* var*iabilis*; 16 and 64 μg/mL	Zhang Y. T. et al. (2022)
Unguidepside C (**262**)	*A. unguis* 158SC-067	A seawater sample, Korea	MZ489151	Anti-*B. subtilis*, *M. luteus*, and *S. aureus*; 22.1 μM	Anh et al. (2022)
Agonodepside C (**263**)	*A. unguis* 158SC-067	A seawater sample, Korea	MZ489151	Anti-*B. subtilis*, *M. luteus*, and *S. aureus*; 8.0, 16.0, and 16.0 μM	Anh et al. (2022)
Aspergilluone A (**264**)	Aspergillus sp. LS57	Marine sponge *Haliclona* sp., Linshui, Hainan province, China	MH862766	Anti-*M. tuberculosis*, *S. aureus*, *B. subtilis*, and *E. coli*; 32, 64, 128 and 128 μg/mL	Liu et al. (2021)
Phomaligol A (**265**)	*A. flavus* MFA500	Marine green algae *Codium fragile*, the GeoMun Island, Yeosu, Korea	–	Anti-*S. aureus* and MRSA; 31.2 μg/mL	Yang et al. (2011)
Trypacidin (**266**)	*A. fumigatus* MF029	Marine sponge *H. perleve*, the Bohai Sea	MH974808	Anti-BCG, *B. subtilis* ATCC 6633, MRSA, and *S. aureus*; 1.25, 12.5, 50, and 50 μg/mL	Song Z. J. et al. (2021)
(+)-Geodin (**267**)	*A. versicolor* TA01-14	Marine gorgonian *Carijoa* sp., the South China Sea	KP759286	Anti-*S. albus*, *S. aureus*, and *V. anguillarum*; 25 μM	Zhang et al. (2019)
Chlorotrypacidin (**268**)	*A. versicolor* TA01-14	Marine gorgonian *Carijoa* sp., the South China Sea	KP759286	Anti-*S. albus*, *S. aureus*, and *V. anguillarum*; 25 μM	Zhang et al. (2019)
Eugenitol (**269**)	*Aspergillus* sp. SCSIO41407	Mangrove sediment sample, Sanya, Hainan province, China	–	Anti-MRSA; 485.4 μM	Cai et al. (2021)
7*β*,8*β*-Epoxy-(22*E*,24*R*)-24-methy-Lcholesta-4,22-diene-3,6-dione (**270**)	A. penicillioides SD-311	Deep-sea sediment, the South China Sea	MH779840	Anti-*V. anguillarum*; 32 μg/mL	Chi et al. (2021b)
Ergosta-4,6,8(14),22-tetraene-3-one (**271**)	A. penicillioides SD-311	Deep-sea sediment, the South China Sea	MH779840	Anti-*E. itarda* and *M. luteus*; 16 μg/mL	Chi et al. (2021b)
Isocyathisterol (**272**)	*A. ustus* cf-42	Marine green alga *C. fragile*, the Zhoushan Island, Zhejiang, China	JX036023	Weak (anti-*E. coli* and *S. aureus*); Inhibitory diameters 6.7 and 5.7 mm at 30 μg/disk	Liu et al. (2014)
Aspersteroid A (**273**)	*A. flavus* YJ07-1	the Bohai sea	–	Anti-*V. anguillarum*, V. parahemolyticus, and *V. alginolyticus*; 12.5 μg/mL	Yang M. Y. et al. (2018)
3*β*-Hydroxy-5*ɑ*,6*β*-methox-yergosta-7,22-dien-15-one (**274**)	*Aspergillus* sp. NR151817	Marine sponge *Coelocarteria* sp., Hainan province, China	NR151817	Anti-*S. aureus*; 64.0 μg/mL	Wen et al. (2024)
Helvolic acid (**275**)	*Aspergillus* sp. SCS-KFD66	A bivalve mollusk *Schisandra chinensis*, the Haikou Bay, Hainan province, China	MK085984	Anti-*S. aureus* and *L. monocytogenes*; 2 and 128 μg/mL	An et al. (2018)
16-*O*-propionyl-16-*O*-deacetylhelvolic acid (**276**)	*A. fumigatus* HNMF0047	Marine sponge, the beach of Wenchang, Hainan province, China	MH101462	Anti-*S. agalactiae* and *S. aureus*; 16.0 μg/mL	Kong et al. (2018)
6-*O*-propionyl-6-*O*-deacetylhelvolic acid (**277**)	*A. fumigatus* HNMF0047	Marine sponge, the beach of Wenchang, Hainan province, China	MH101462	Anti-*S. agalactiae* and *S. aureus*; 2 and 8 μg/mL	Kong et al. (2018)
24-Epi-6*β*,16*β*-diacetoxy-25-hydroxy-3,7-dioxo-29-nordammara-1,17(20)-diene-21,24-lactone (**278**)	*A. fumigatus* HNMF0047	Marine sponge, the beach of Wenchang, Hainan province, China	MH101462	Anti-*S. agalactiae*; 64 μg/mL	Kong et al. (2018)
3,7-Diketo-cephalosporin P_1_ (**279**)	*A. fumigatus* SCSIO 41012	Deep-sea sediment, the Indian Ocean	KM924435	Anti-*A. baumanii* ATCC 19606; 50 μg/mL	Limbadri et al. (2018)
22-*O*-acetylisocyclocitrinol A (**280**)	*A. fumigatus* SCSIO 41012	Deep-sea sediment, the Indian Ocean	KM924435	Anti-*A. baumanii* ATCC 15122 and *K. pneumonia* ATCC 14578; 12.5 and 3.125 μg/mL	Limbadri et al. (2018)
Fusidic acid (**281**)	*A. flavus* JK07-1	Marine sediment, the Huanghua, the Bohai Sea	–	Anti-M. lysodeikticus, *B. cereus*, *B. megaterium*, *B. anthracis*, and *S. typhi*; 0.07, 0.07, 0.07, 0.30, and 0.60 μM	Ren et al. (2020)
Neocyclocitrinol D (**282**)	*A. flavus* JK07-1	Marine sediment, the Huanghua, the Bohai Sea	–	Anti-M. lysodeikticus; 1.30 μM	Ren et al. (2020)
Aspergillsteroid A (**283**)	Aspergillus sp. LS116	Marine sponge *Haliclona* sp., Linshui, Hainan province, China	–	Anti-*V. harveyi*; 16 μg/mL	Xu P. et al. (2020)
Neocyclocitrinol B (**284**)	Aspergillus sp. LS116	Marine sponge *Haliclona* sp., Linshui, Hainan province, China	–	Anti-*V. harveyi*; 128 μg/mL	Xu P. et al. (2020)
Demethylincisterol A_2_ (**285**)	*A. hiratsukae* SCSIO 5Bn1003	Marine coral, the South China Sea	KY806121.1	Anti-*B. subtilis*; 10.26 μg/mL	Zeng et al. (2022a)
Punicesterone B (**286**)	*A. puniceus* SCSIO z021	Deep-sea sediment, the Okinawa Trough	KX258801	Anti-*S. iniae*, *S. agalactiae*, *E. coli*, *B. subtilis*, and *S. aureus*; 65.8, 65.8, 65.8, 32.9, and 32.9 μM	Huang et al. (2023)
Punicesterone C (**287**)	*A. puniceus* SCSIO z021	Deep-sea sediment, the Okinawa Trough	KX258801	Anti-*S. iniae*, *S. agalactiae*, *E. coli*, *B. subtilis*, and *S. aureus*; 65.8, 65.8, 65.8, 32.9, and 32.9 μM	Huang et al. (2023)
3-Hydroxy-5-(3-hydroxy-5-methylphenoxy)-4-methoxybenzoic acid (**288**)	*A. carneus*	Seawater sample, Sanya, Hainan Province, China	KX437770	Anti-*S. aureus*, *V. anguillarum*, and *E. coli*; 25.0 μM	Xu et al. (2017)
3,4-Dihydroxy-5-(3-hydroxy-5-methylphenoxy)benzoic acid (**289**)	*A. carneus*	Seawater sample, Sanya, Hainan Province, China	KX437770	Anti-*S. aureus*, *V. anguillarum*, and *E. coli*; 25.0 μM	Xu et al. (2017)
3-Hydroxy-5-(3-hydroxy-5-methylphenoxy)benzoic acid (**290**)	*A. carneus*	Seawater sample, Sanya, Hainan Province, China	KX437770	Anti-*S. aureus*, *V. anguillarum*, and *E. coli*; 25.0 μM	Xu et al. (2017)
Aspergetherin C (**291**)	*A. terreus* 164,018	Marine sponge *Dysidea* sp., the South China Sea	–	Anti-MRSA; 64 μg/mL	Li J. X. et al. (2023)
Methyl 3,5-dichloroasterric acid (**292**)	*A. terreus* 164,018	Marine sponge *Dysidea* sp., the South China Sea	–	Anti-MRSA 05–72 and USA300; 1.0 and 16 μg/mL	Li J. X. et al. (2023)
Methyl chloroasterrate (**293**)	*A. terreus* 164,018	Marine sponge *Dysidea* sp., the South China Sea	–	Anti-MRSA; 64 μg/mL	Li J. X. et al. (2023)
Dimethyl 2,3′-dimethylosoate (**294**)	*A. fumigatus* H22	Middle seawater, the Western Pacific	–	Anti-MRSA; 5 μM	Zhang R. et al. (2022)
4-Methylcarbonyldiorcinol (**295**)	*A. versicolor* OUCMDZ-2738	Marine alga *Epiactis prolifera*, the Shilaoren beach, Qingdao, Shandong province, China	MH150818	Anti-*P. aeruginosa*, *C. perfringens*, and *S. aureus*; 13.9, 55.6, and 55.6 μM	Liu et al. (2019)
Diorcinol K (**296**)	*Aspergillus* sp. CUGB-F046	Sediment sample, the Bohai Sea	–	Anti-*S. aureus* and MRSA; 3.125 μg/mL	Xu et al. (2018)
Diorcinol D (**297**)	*Aspergillus* sp. CUGB-F046	Sediment sample, the Bohai Sea	–	Anti-*S. aureus* and MRSA; 6.25 μg/mL	Xu et al. (2018)
Diorcinol I (**298**)	*Aspergillus* sp. CUGB-F046	Sediment sample, the Bohai Sea	–	Anti-*S. aureus* and MRSA; 6.25 μg/mL	Xu et al. (2018)
Diorcinol (**299**)	*A. versicolor* 170,217	the intestinal contents of a whale *Mesoplodon densirostris*, the East China Sea	SUB13826338	Anti-V. parahemolyticus; 128 μM	Lin S. H. et al. (2023)
Violaceol-I (**300**)	*Aspergillus* sp. ZZ1861	Sea mud sample, the Zhoushan Island, Zhejiang province, China	OR985107	Anti-MRSA and *E. coli*; 50 and 6.25 μg/mL	Ha et al. (2024)
Violaceol-II (**301**)	*Aspergillus* sp. ZZ1861	Sea mud sample, the Zhoushan Island, Zhejiang province, China	OR985107	Anti-MRSA and *E. coli*; 50 and 6.25 μg/mL	Ha et al. (2024)
4-Carbethoxydiorcinal (**302**)	*Aspergillus* sp. ZZ1861	Sea mud sample, the Zhoushan Island, Zhejiang province, China	OR985107	Anti-MRSA; 25 μg/mL	Ha et al. (2024)
1,9-Dimethyl-3,7-dibenzofurandiol (**303**)	*Aspergillus* sp. ZZ1861	Sea mud sample, the Zhoushan Island, Zhejiang province, China	OR985107	Anti-*E. coli*; 12.5 μg/mL	Ha et al. (2024)
Aspergillusether E (**304**)	*A. unguis* PSU-MF16	Marine sponge *Dysidea* sp., the Koh Bulon Mai Pai, Satun Province, Thailand	KY397987	Anti-*S. aureus* and MRSA; 16 μg/mL	Saetang et al. (2021)
Aspergillusether C (**305**)	*A. unguis* PSU-MF16	Marine sponge *Dysidea* sp., the Koh Bulon Mai Pai, Satun Province, Thailand	KY397987	Anti-*S. aureus* and MRSA; 64 μg/mL	Saetang et al. (2021)
Aspergillusether D (**306**)	*A. unguis* PSU-MF16	Marine sponge *Dysidea* sp., the Koh Bulon Mai Pai, Satun Province, Thailand	KY397987	Anti-*S. aureus* and MRSA; 64 and 128 μg/mL	Saetang et al. (2021)
Pilobolusate (**307**)	*A. unguis* PSU-MF16	Marine sponge *Dysidea* sp., the Koh Bulon Mai Pai, Satun Province, Thailand	KY397987	Anti-*S. aureus* and MRSA; 64 μg/mL	Saetang et al. (2021)
Aspergillusether J (**308**)	*A. unguis* GXIMD 02505	Marine coral *P. damicornis*, the Weizhou Islands, Guangxi, China	OL989238	Anti-MRSA, *M. variabilis*, and *M. jannaschii*; 16, 32 and 64 μg/mL	Zhang Y. T. et al. (2022)
Aspergillusether *F* (**309**)	*A. unguis* GXIMD 02505	Marine coral *P. damicornis*, the Weizhou Islands, Guangxi, China	OL989238	Anti-MRSA, *M. variabilis*, and *M. jannaschii*; 2, 16, and 32 μg/mL	Zhang Y. T. et al. (2022)
Flavuside A (**310**)	*A. flavus* MFA500	Marine green algae *C. fragile*, the GeoMun Island, Yeosu, Korea	–	Anti-MRSA; 15.6 μg/mL	Yang et al. (2011)
Flavuside B (**311**)	*A. flavus* MFA500	Marine green algae *C. fragile*, the GeoMun Island, Yeosu, Korea	–	Anti-MRSA; 15.6 μg/mL	Yang et al. (2011)
Acetylpeniciphenol (**312**)	*A. insuetus* SD-512	Deep-sea sediment, the South China Sea	MN696202	Anti-E. itarda, *V. alginolyticus*, and *V. vulnificus*; 4, 8, and 8 μg/mL	Chi et al. (2021a)
Fumagiringillin (**313**)	*A. fumigatus* H22	middle seawater, the Western Pacific	–	Anti-MRSA; 25.0 μM	Zhang R. et al. (2022)
Fumagillin (**314**)	*A. fumigatus* H22	middle seawater, the Western Pacific	–	Anti-MRSA; 2.50 μM	Zhang R. et al. (2022)
8-*O*-4-dehydrodiferulic acid (**315**)	Aspergillus sp.	Marine sponge *T. aurantium*, the Adriatic Sea	–	Anti-*R. litoralis*; 1 μg/mL	Zhou et al. (2014)
Penicitrinol L (**316**)	A. sydowii EN-534 and P. citrinum EN-535	Marine red alga *L. okamurai*, Qingdao, Shandong province, China	MG242135 MG242136	Anti-*E. coli*, *E. ictaluri*, and *V. alginolyticus*; 64 μg/mL	Yang et al. (2018b)
penicitrinol A (**317**)	A. sydowii EN-534 and P. citrinum EN-535	Marine red alga *L. okamurai*, Qingdao, Shandong province, China	MG242135 MG242136	Anti-*V. alginolyticus*, *E. coli*, *V. parahaemolyticus*, *M. luteus*, and *E. ictaluri*; 32, 8, 8, 4, and 16 μg/mL	Yang et al. (2018b)
	*A. versicolor* 170,217	the intestinal contents of a whale *M. densirostris*, the East China Sea	SUB13826338	Anti-V. parahemolyticus; 256 μg/mL	Lin S. H. et al. (2023)
2-(Hydroxymethyl)-3-propylphenol (**318**)	Aspergillus sp. ZJ-68	Mangrove plant *K. candel*, the Zhanjiang Mangrove Nature Reserve, Guangdong Province, China	MK629267	Anti-*S. aureus*, *E. coli*, and *B. subtilis*; 4.15, 8.3, and 8.3 μg/mL	Cai et al. (2019)
(−)-Brassicadiol (**319**)	Aspergillus sp. ZJ-68	Mangrove plant *K. candel*, the Zhanjiang Mangrove Nature Reserve, Guangdong Province, China	MK629267	Anti-*S. aureus*, *E. coli*, and *B. subtilis*; 12.5 μg/mL	Cai et al. (2019)
4,6-Dichloro-5-methyl-benzene-1,3-diol (**320**)	*A. terreus* CC-S06-18	A seawater sample, the Pacific Ocean	MN463005	Anti-*V. parahaemolyticus*; 7.8 μg/mL	Huang et al. (2024)
1-(2,6-Dihydroxy-4-methoxy-3,5-dimethylphenyl)-2-methylbutan-1-one (**321**)	*A. unguis* GXIMD 02505	Marine coral *P. damicornis*, the Weizhou Islands, Guangxi, China	OL989238	Anti-*M. variabilis* and *M. jannaschii*; 8 and 32 μg/mL	Zhang Y. T. et al. (2022)
Asperporonin A (**322**)	*A. terreus* SCSIO 41202	Deep-sea sediment, the coast of the South China Sea	MN613535	Anti-*X. citri* subsp. *citri*; 0.3125 mg/mL	Zhang et al. (2024)
Asperporonin B (**323**)	*A. terreus* SCSIO 41202	Deep-sea sediment, the coast of the South China Sea	MN613535	Anti-*X. citri* subsp. *citri*; 0.3125 mg/mL	Zhang et al. (2024)
Terrusnolide A (**324**)	*Aspergillus* sp. SCSIO 41029	Deep-sea sediment, the South China	MH591418.1	Anti-*S. aureus*; 6.25 μg/mL	Chen et al. (2021)
Candidusin A (**325**)	*Aspergillus* sp. SCSIO 40435	Marine coral, the South China sea	–	Anti-*E. coli*, *A. baumannii*, and *S. aureus*; 1, 64, and 32 μg/mL	Ye et al. (2022)
Terphenyllin (**326**)	*Aspergillus* sp. SCSIO 40435	Marine coral, the South China sea	–	Anti-*E. coli*; 0.5 μg/mL	Ye et al. (2022)
4″-Deoxyterphenyllin (**327**)	*Aspergillus* sp. SCSIO 40435	Marine coral, the South China sea	–	Anti-*B. subtilis* and *M. luteus*; 64 and 32 μg/mL	Ye et al. (2022)
5[(3*E*,5*E*)-Nona-3,5-dien-1-yl]benzene (**328**)	*A. stellatus* KUFA 2017	Marine sponge *Mycale* sp., the Samaesan Island, Chonburi province, Thailand	MZ331807	Anti-*E. faecalis* ATCC 29212, VRE, *S. aureus* ATCC 29213, and MRSA; 16. 16, 32, and 16 μg/mL	Machado et al. (2022)
(9*R*,10*E*,12*E*)-9-Methoxyoc Tadecadienoic acid (**329**)	*A. terreus* SCSIO 41202	Deep-sea sediment, the coast of the South China Sea	MN613535	Anti-*X. citri* subsp. *citri*; 0.078 mg/mL	Zhang et al. (2024)
Carnemycin H (**330**)	*A. ustus*	Mangrove sediments, the Zhangjiangkou Mangrove National Nature Reserve, Fujian province, China	MN650842	Anti-*R. solanacearum*; 25 μg/mL	Xue et al. (2024)
Carnemycin I (**331**)	*A. ustus*	Mangrove sediments, the Zhangjiangkou Mangrove National Nature Reserve, Fujian province, China	MN650842	Anti-*R. solanacearum*; 15 μg/mL	Xue et al. (2024)
Stromemycin B (**332**)	*A. ustus*	Mangrove sediments, the Zhangjiangkou Mangrove National Nature Reserve, Fujian province, China	MN650842	Aanti-*R. solanacearum*; 3 μg/mL	Xue et al. (2024)
Carnemycin E (**333**)	*A. ustus*	Mangrove sediments, the Zhangjiangkou Mangrove National Nature Reserve, Fujian province, China	MN650842	Anti-*R. solanacearum*; 35 μg/mL	Xue et al. (2024)
Carnemycin B (**334**)	*A. ustus*	Mangrove sediments, the Zhangjiangkou Mangrove National Nature Reserve, Fujian province, China	MN650842	Anti-*R. solanacearum*; 30 μg/mL	Xue et al. (2024)
Carnemycin A (**335**)	*A. ustus*	Mangrove sediments, the Zhangjiangkou Mangrove National Nature Reserve, Fujian province, China	MN650842	Anti-*R. solanacearum*; 25 μg/mL	Xue et al. (2024)
2,4-Dihydroxy-6-[(3*E*,5*E*)-nona-3,5-dien-1-yl]-benzoic acid (**336**)	*A. ustus*	Mangrove sediments, the Zhangjiangkou Mangrove National Nature Reserve, Fujian province, China	MN650842	Anti-*R. solanacearum*; 5 μg/mL	Xue et al. (2024)
Stromemycin (**337**)	*A. ustus*	Mangrove sediments, the Zhangjiangkou Mangrove National Nature Reserve, Fujian province, China	MN65084	Anti-*R. solanacearum*; 8 μg/mL	Xue et al. (2024)

**Figure 15:**
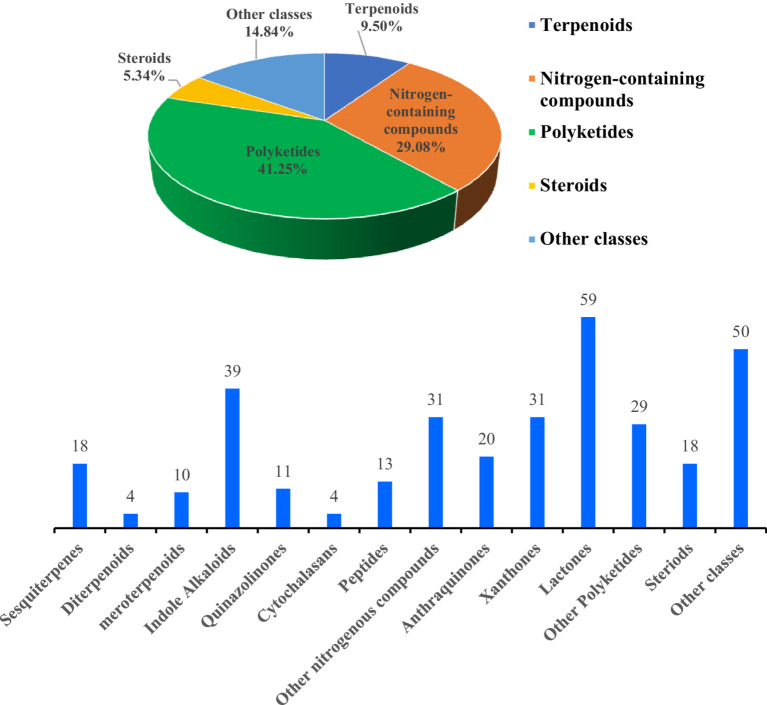
Structural diversity of the antibacterial secondary metabolites from the genus of *Aspergillus* (January 2010 to June 2024).

**Figure 16:**
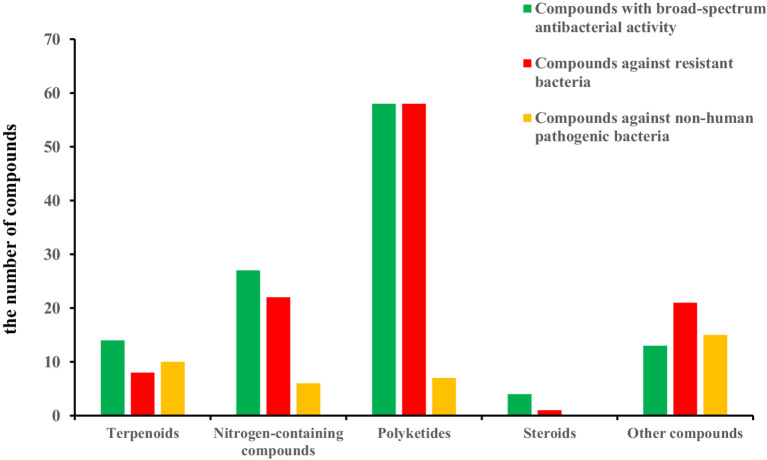
The number and types of compounds with broad-spectrum antibacterial activity, activity against resistant bacteria, and activity against non-human pathogenic bacteria.

The structural diversities of the antibacterial secondary metabolites isolated from *Aspergillus* spp. are shown in Figure 15. The reported numbers of *Aspergillus* were based on structural classification, including 32 terpenoids, 98 nitrogen-containing compounds, 139 polyketides, 18 steroids, and 50 other derivatives discovered. The number and types of compounds with broad-spectrum antibacterial activity, activity against resistant bacteria, and activity against non-human pathogenic bacteria are shown in Figure 16.

Interesting, the conjugated double bonds at C-16 and C-18 are essential for the antibacterial activities of the ophiobolin sesterterpenes when having −CH_2_OH (**2**) or −CHO (**3**) groups positioned at C-7 (Chi et al., 2020). Notoamides (**69**–**71**, **118,** and **119**) are featured by the conserved moieties of a pyranoindole ring and a proline-bearing bicyclo[2.2.2]diazaoctane core. Sclerotiamide L (**65**) with a 6,6,5,7,6,5-ring system inhibited pathogenic bacteria including methicillin-resistant *S. aureus* (Meng et al., 2022). Nevertheless, this study provides indole diketopiperazine alkaloids as the undescribed natural scaffolds for the development of antibacterial agents. A large number of depsidone derivatives (**203**–**221**) had antibacterial activity against *S. aureus* and MRSA has been reported in the literature (Handayani et al., 2020; Zhang Y. T. et al., 2022; Thi et al., 2023; Saetang et al., 2021). The possible and preliminary structure–activity relationship was discussed; the phenolic hydroxyl group can improve the activity. Natural polyphenol compounds have significant antimicrobial activity (Chen et al., 2024). The chlorine-substituted group can be beneficial for the activity.

**Figure 17:**
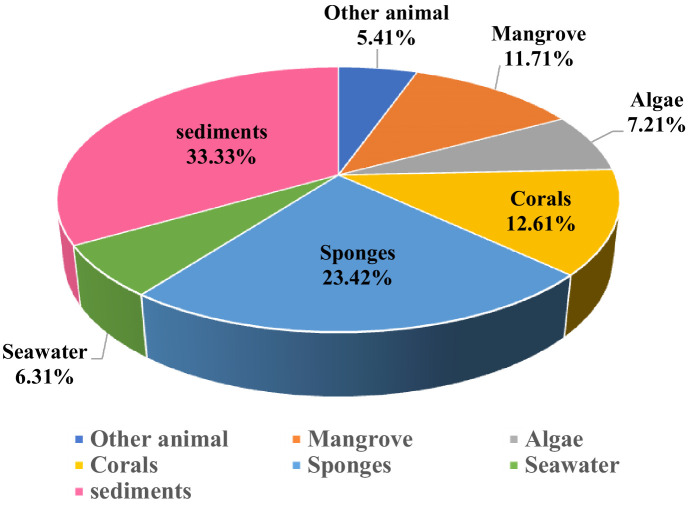
The proportion of *Aspergillus* from different marine sources.

We sorted out the different marine sources of these *Aspergillus* spp., such as marine algae, corals, sponges, other animals, mangroves, seawater, and marine sediments, are shown in Figure 17. The most *Aspergillus* spp. were derived from marine sediment, accounting for 33.33%, and from marine sponges ranked second, comprising 23.42% of the total.

**Figure 18:**
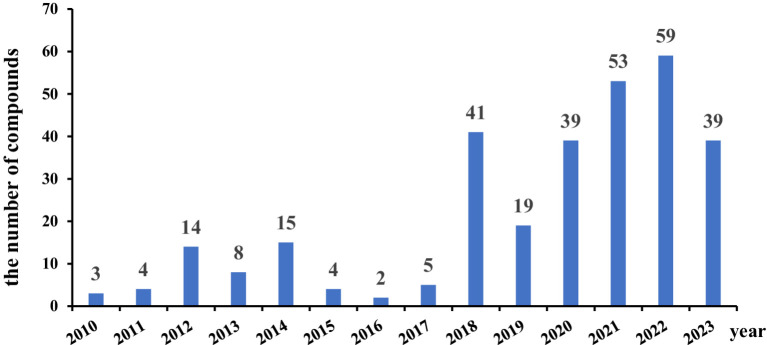
Each year of the antibacterial secondary metabolites from the genus of *Aspergillus* (2010–2023) (the data for 2024 is not accurate, so it will not be included).

The number of antibacterial secondary metabolites from the genus of *Aspergillus* annually from 2010 to 2023 is shown in Figure 18. The progress of research in antimicrobial compounds from the genus *Aspergillus* was relatively slow from 2010 to 2017. However, there has been rapid development in antimicrobial research since 2018. These data indicated that research related to antibacterial compounds from *Aspergillus* spp. is increasingly receiving attention. Many of these compounds show inhibitory effects against *S. aureus*, while some showed activity against *E. coli* and *B. subtilis*. These active compounds hold promise for treating bacterial infections, offering valuable insights for the development of new anti-infective drugs.

Notably, some antimicrobial compounds produced by *Aspergillus* fungi also showed activities against agriculture and fish pathogenic bacteria and so on. For example, asperalin E (**115**), with a rare 4-amino-2-butanone moiety, exhibited the strongest inhibitory effects against fish pathogenic bacterium *S. iniae*, with potential for development as a new bactericide, and asperalin *F* (**116**) showed moderate-to-potent inhibitory activity against three fish pathogenic bacterium among *E. ictalurid*, *S. iniae*, and *S. parauberis*, with potential for development as a new bactericide. (9*R*,10*E*,12*E*)-9-methoxyoctadecadienoic acid (**329**) exhibited an excellent anti-*Xanthomonas citri* subsp. *citri* effect with the MIC value of 0.078 mg/mL, which was significantly more potent than the positive control CuSO_4_ (MIC, 0.3125 mg/mL). Compound **329** inhibited cell growth by disrupting biofilm formation, destroying the cell membrane, and inducing the accumulation of reactive oxygen species. Compound **6** is highly effective in controlling citrus canker disease *in vivo* tests, indicating **6** has the potential to lead compound for the development of new environmentally friendly and efficient anti-Xcc pesticides (Zhang et al., 2024). Stromemycin B (**332**) could effectively control the development of wilting symptoms and considerably minimize the occurrence of bacterial wilt in tomato plants. At 14 days after inoculation, compound **332** exerted a controlled efficacy of over 80% at a concentration of 100 μg/mL, which was better than that of streptomycin sulfate (100 μg/mL), indicating that compound **332** was a significant candidate as an antibacterial agent against *Ralstonia solanacearum* (Xue et al., 2024). These results suggested that the antibacterial lead compounds might be used as one of the probable candidates’ drugs for “One Health” in the utilization in healthcare, agriculture, and fishery.

## 4 Conclusion

337 secondary metabolites (including 145 new compounds) were isolated from marine-derived *Aspergillus* fungi; the compounds were classified into five chemical types: 32 terpenoids, 98 nitrogen-containing compounds, 139 polyketides, 18 steroids, and 50 other derivatives (Figure 15). The distribution of these compounds is as follows: terpenoids (9.50%), nitrogen-containing compounds (29.08%), polyketides (41.25%), steroids (5.34%), and other compounds (14.84%). Polyketides displayed the most substantial proportion of the observed antibacterial compounds, alongside notable contributions from terpenoids and nitrogen-containing compounds. This comprehensive analysis highlights the potential for developing antimicrobial agents from these natural products.

Additionally, the samples were obtained from various environments: 7.21% from algae, 12.61% from corals, 23.42% from sponges, 5.41% from other animals, 11.71% from mangroves, and 6.31% from seawater. Most significantly, 33.33% originated from sediment samples (Figure 18). This extensive environmental sampling underscores the compounds’ efficacy and potential applications in combating antibiotic-resistant bacteria. Specifically, terpenoid compounds were classified as 18 sesquiterpenes, four diterpenes, and 10 meroterpenoids. Nitrogen-containing compounds included 39 indole alkaloids, 11 quinazolinone alkaloids, four cytochalasan alkaloids, 13 peptides, and 31 other nitrogen-containing compounds. Polyketide compounds were identified as 20 anthraquinones, 31 xanthones, 59 lactones, and 29 other polyketide metabolites. 18 steriods and 50 other classes are shown in Figure 15. We observed that research progress in antimicrobial compounds from the genus of *Aspergillus* was relatively slow from 2010 to 2017. However, there has been rapid development in antimicrobial research since 2018. These data indicated that research related to antibacterial compounds from *Aspergillus* spp. are increasingly receiving attention. By classifying multiple antibacterial compounds, a foundation is laid for predicting which types may exert more potent pharmacological effects on specific biological targets, guiding drug design and validation through simulation or experimentation.

Among all antibacterial active compounds, some were found to have activity levels approaching or reaching the nanomolar range, such as fumigatoside *F* (**65**), cytochalasin Z17 (**75**), dihydroisoflavipucine (**90**), emeguisin A (**204**), and fusidic acid (**265**). As a first-in-class BCG-selective diketopiperazine dimer antibiotic, brevianamide S (**34**) was indicative of a possible new mechanism of action that could, if translated to *M. tuberculosis*, represent a valuable new lead in the search for next-generation antitubercular drugs. These compounds could become promising lead compounds for use as antimicrobial agents in the future. Notably, some antimicrobial compounds produced by *Aspergillus* fungi also showed activities against agriculture and fish pathogenic bacteria, and so on.

In summary, the chemical diversity and potent antibacterial activities ofsecondary metabolites from marine-derived *Aspergillus* species indicated their potential in antibiotic drug discovery. The identified metabolites demonstrate a wide range of antimicrobial activities, showing potent effects against various pathogens. Future research aims to elucidate their mechanisms of action and optimize production methods to fully harness their therapeutic potential in fighting infectious diseases. Marine-derived *Aspergillus* species present a promising frontier for developing novel natural products with applications in medical treatments and agricultural antimicrobial agents.

## References

[ref1] Alahmari A. N. Hassoubah S. A. Alaidaroos B. A. (2022). Sponges-associated marine bacteria as sources of antimicrobial compounds. Novel. Res. Microbiol. J. 6, 1742–1767. doi: 10.21608/nrmj.2022.267424

[ref2] An C. L. Kong F. D. Ma Q. Y. Xie Q. Y. Yuan J. Z. Zhou L. M.. (2018). Chemical constituents of the marine-derived fungus *Aspergillus* sp. SCS-KFD66. Mar. Drugs 16:468. doi: 10.3390/md16120468, PMID: 30486303 PMC6316597

[ref3] Anh C. V. Kwon J. H. Kang J. S. Lee H. S. Heo C. S. Shin H. J. (2022). Antibacterial and cytotoxic phenolic polyketides from two marine-derived fungal strains of *Aspergillus unguis*. Pharmaceuticals 15:74. doi: 10.3390/ph15010074, PMID: 35056132 PMC8779881

[ref4] Bai Z. Q. Lin X. P. Wang Y. Z. Wang J. F. Zhou X. F. Yang B.. (2014). New phenyl derivatives from endophytic fungus *Aspergillus flavipes* AIL8 derived of mangrove plant *Acanthus ilicifolius*. Fitoterapia 95, 194–202. doi: 10.1016/j.fitote.2014.03.021, PMID: 24704337

[ref5] Bao J. Li X. X. He F. Zhang X. Y. Zhu K. K. Tao H. R.. (2020). Asperbutenolide a, an unusual aromatic butenolide dimer with diverse bioactivities from a marine-derived fungus *Aspergillus terreus* SCAU011. Tetrahedron Lett. 61:152193. doi: 10.1016/j.tetlet.2020.152193

[ref6] Bao J. Li X. X. Zhu K. K. He F. Wang Y. Y. Yu J. H.. (2021). Bioactive aromatic butenolides from a mangrove sediment originated fungal species, *Aspergillus terreus* SCAU011. Fitoterapia 150:104856. doi: 10.1016/j.fitote.2021.104856, PMID: 33582267

[ref7] Buttachon S. Ramos A. A. Inacio A. Dethoup T. Gales L. Lee M.. (2018). Bis-indolyl benzenoids, hydroxypyrrolidine derivatives and other constituents from cultures of the marine sponge-associated fungus *Aspergillus candidus* KUFA0062. Mar. Drugs 16:119. doi: 10.3390/md16040119, PMID: 29642369 PMC5923406

[ref8] Cai J. Chen C. M. Tan Y. H. Chen W. H. Luo X. W. Luo L. X.. (2021). Bioactive polyketide and diketopiperazine derivatives from the mangrove-sediment-derived fungus *Aspergillus* sp. SCSIO41407. Molecules 26:4851. doi: 10.3390/molecules26164851, PMID: 34443439 PMC8399180

[ref9] Cai R. L. Jiang H. M. Zang Z. M. Li C. Y. She Z. G. (2019). New benzofuranoids and phenylpropanoids from the mangrove endophytic fungus, *Aspergillus* sp. ZJ-68. Mar. Drugs 17:478. doi: 10.3390/md17080478, PMID: 31426620 PMC6723808

[ref10] Cai J. Wang X. N. Gan X. Zhou Q. Luo X. W. Yang B.. (2023). New chlorinated metabolites and antiproliferative polyketone from the mangrove sediments-derived fungus *Mollisia* sp. SCSIO41409. Mar. Drugs 21:32. doi: 10.3390/md21010032, PMID: 36662205 PMC9866852

[ref11] Cardoso-Martinez F. De la Rosa J. M. Diaz-Marrero A. R. Darias J. D'Croz L. Cerella C.. (2015). Oximoaspergillimide, a fungal derivative from a marine isolate of *Aspergillus* sp. Eur. J. Org. Chem. 2015, 2256–2261. doi: 10.1002/ejoc.201403668

[ref12] Carroll A. R. Copp B. R. Davis R. A. Keyzers R. A. Prinsep M. R. (2024). Marine natural products. Nat. Prod. Rep. 41, 162–207. doi: 10.1039/D3NP00061C 38285012

[ref13] Cen S. Y. Jia J. Ge Y. C. Ma Y. H. Li X. Y. Wei J. H.. (2021). A new antibacterial 3,5-dimethylorsellinic acid-based meroterpene from the marine fungus *Aspergillus* sp. CSYZ-1. Fitoterapia 152:104908. doi: 10.1016/j.fitote.2021.104908, PMID: 33892126

[ref14] Charani E. Mendelson M. Pallett S. J. C. Ahmad R. Mpundu M. Mbamalu O.. (2023). An analysis of existing national action plans for antimicrobial resistance-gaps and opportunities in strategies optimising antibiotic use in human populations. Lancet Glob. Health 11, e466–e474. doi: 10.1016/S2214-109X(23)00019-0, PMID: 36739875

[ref15] Chen W. H. Chen C. M. Long J. Y. Lan S. J. Lin X. P. Liao S. R.. (2021). Bioactive secondary metabolites from the deep-sea derived fungus *Aspergillus* sp. SCSIO 41029. J. Antibiot. 74, 156–159. doi: 10.1038/s41429-020-00378-y, PMID: 33106626

[ref16] Chen M. Fu X. M. Kong C. J. Wang C. Y. (2014). Nucleoside derivatives from the marine-derived fungus *Aspergillus versicolor*. Nat. Prod. Res. 28, 895–900. doi: 10.1080/14786419.2014.891114 24670197

[ref17] Chen X. N. Lan W. Q. Xie J. (2024). Natural phenolic compounds: antimicrobial properties, antimicrobial mechanisms, and potential utilization in the preservation of aquatic products. Food Chem. 440:138198. doi: 10.1016/j.foodchem.2023.138198 38128429

[ref18] Chen W. H. Liu H. Y. Long J. Y. Tao H. M. Lin X. P. Liao S. R.. (2020). Asperpentenone a, a novel polyketide isolated from the deep-sea derived fungus *Aspergillus* sp. SCSIO 41024. Phytochem. Lett. 35, 99–102. doi: 10.1016/j.phytol.2019.11.009

[ref19] Chen B. Qiu P. J. Xu B. F. Zhao Q. M. Gu Y. C. Fu L.. (2022). Cytotoxic and antibacterial isomalabaricane terpenoids from the sponge *Rhabdastrella globostellata*. J. Nat. Prod. 85, 1799–1807. doi: 10.1021/acs.jnatprod.2c00348, PMID: 35767002

[ref20] Chen M. Shao C. L. Fu X. M. Xu R. F. Zheng J. J. Zhao D. L.. (2013). Bioactive indole alkaloids and phenyl ether derivatives from a marine-derived *Aspergillus* sp. fungus. *J*. *Nat. Prod*. 76:1229. doi: 10.1021/np400465r 23527875

[ref21] Chen X. Y. Zeng Q. Chen Y. C. Zhong W. M. Xiang Y. Wang J. F.. (2022). Chevalones H-M: six new *α*-pyrone meroterpenoids from the gorgonian coral-drived fungus *Aspergillus hiratsukae* SCSIO 7S2001. Mar. Drugs 20:71. doi: 10.3390/md20010071, PMID: 35049926 PMC8781156

[ref22] Chi L. P. Li X. M. Wan Y. P. Li Y. H. Li X. Wang B. G. (2021a). Two new phenol derivatives from the cold seep-derived fungus *Aspergillus insuetus* SD-512. Chem. Biodivers. 18:e2100512. doi: 10.1002/cbdv.202100512, PMID: 34347345

[ref23] Chi L. P. Li X. M. Wan Y. P. Li X. Wang B. G. (2020). Ophiobolin sesterterpenoids and farnesylated phthalide derivatives from the deep sea cold-seep-derived fungus *Aspergillus insuetus* SD-512. J. Nat. Prod. 83, 3652–3660. doi: 10.1021/acs.jnatprod.0c00860, PMID: 33322904

[ref24] Chi L. P. Liu D. Li X. M. Wan Y. P. Wang B. G. Li X. (2023). Aspertides A-E: antimicrobial pentadepsipeptides with a unique p-methoxycinnamoyl amide group from the marine isolates *Aspergillus tamarii* MA-21 and *Aspergillus insuetus* SD-512. J. Agr. Food. Chem. 71, 13316–13324. doi: 10.1021/acs.jafc.3c02610, PMID: 37650146

[ref25] Chi L. P. Yang S. Q. Li X. M. Li X. D. Wang B. G. Li X. (2021b). A new steroid with 7β,8β-epoxidation from the deep sea-derived fungus *Aspergillus penicillioides* SD-311. J. Asian Nat. Prod. Res. 23, 884–891. doi: 10.1080/10286020.2020.1791096, PMID: 32657145

[ref26] de Alcântara Rodrigues I. Ferrari R. G. Panzenhagen P. H. N. Mano S. B. Conte C. A. J. (2020). Antimicrobial resistance genes in bacteria from animal-based foods. Adv. Appl. Microbiol. 112, 143–183. doi: 10.1016/bs.aambs.2020.03.001, PMID: 32762867

[ref27] Ding L. J. Ren L. Li S. Song J. J. Han Z. W. He S.. (2019). Production of new antibacterial 4-hydroxy-*α*-pyrones by a marine fungus *Aspergillus niger* cultivated in solid medium. Mar. Drugs 17:344. doi: 10.3390/md17060344, PMID: 31185700 PMC6627810

[ref28] Dong Y. L. Li X. M. Shi X. S. Wang Y. R. Wang B. G. Meng L. H. (2023a). Diketopiperazine alkaloids and bisabolene sesquiterpenoids from *Aspergillus versicolor* AS-212, an endozoic fungus associated with deep-sea coral of magellan seamounts. Mar. Drugs 21:293. doi: 10.3390/md21050293, PMID: 37233487 PMC10224163

[ref29] Dong Y. L. Li X. M. Wang Y. R. Shi X. S. Wang B. G. Meng L. H. (2023b). Oxepine-containing pyrazinopyrimidine alkaloids and quinolinone derivatives produced by *Aspergillus versicolor* AS-212, a deep-sea-derived endozoic fungus. Fitoterapia 168:105559. doi: 10.1016/j.fitote.2023.105559, PMID: 37271296

[ref30] Duraes F. Szemeredi N. Kumla D. Pinto M. Kijjoa A. Spengler G. (2021). Metabolites from marine-derived fungi as potential antimicrobial adjuvants. Mar. Drugs 19:475. doi: 10.3390/md19090475, PMID: 34564137 PMC8470461

[ref31] Gou X. S. Jia J. Xue Y. X. Ding W. J. Dong Z. T. Tian D. M.. (2020). New pyrones and their analogs from the marine mangrove-derived *Aspergillus* sp. DM94 with antibacterial activity against *Helicobacter pylori*. Appl. Microbiol. Biotechnol. 104, 7971–7978. doi: 10.1007/s00253-020-10792-9, PMID: 32700088

[ref32] Gow N. A. R. Johnson C. Berman J. Coste A. T. Cuomo C. A. Perlin D. S.. (2022). The importance of antimicrobial resistance in medical mycology. Nat. Commun. 13:5352. doi: 10.1038/s41467-022-32249-5, PMID: 36097014 PMC9466305

[ref33] Graziano T. S. Cuzzullin M. C. Franco G. C. Schwartz-Filho O. Dias D. A. E. Groppo F. C.. (2015). Statins and antimicrobial effects: simvastatin as a potential drug against *Staphylococcus aureus* biofilm. PLoS One 10:e0128098. doi: 10.1371/journal.pone.0128098, PMID: 26020797 PMC4447369

[ref34] Guo C. Wang P. Pang X. Y. Lin X. P. Liao S. R. Yang B.. (2021). Discovery of a dimeric zinc complex and five cyclopentenone derivatives from the sponge-associated fungus *Aspergillus ochraceopetaliformis*. ACS Omega 6, 8942–8949. doi: 10.1021/acsomega.0c06218 33842764 PMC8028006

[ref35] Guo Z. K. Zhou Y. Q. Han H. Wang W. Xiang L. Deng X. Z.. (2018). New antibacterial phenone derivatives asperphenone A-C from mangrove-derived fungus *Aspergillus* sp. YHZ-1. Mar. Drugs 16:45. doi: 10.3390/md16020045, PMID: 29385686 PMC5852473

[ref36] Ha Y. R. Zhou Y. F. Ma M. Z. Wang N. Wang P. B. Zhang Z. Z. (2024). Antimicrobial metabolites from the marine-derived fungus *Aspergillus* sp. ZZ1861. Phytochemistry 224:114164. doi: 10.1016/j.phytochem.2024.114164 38797256

[ref37] Haenni M. Dagot C. Chesneau O. Bibbal D. Labanowski J. Vialette M.. (2022). Environmental contamination in a high-income country (France) by antibiotics, antibiotic-resistant bacteria, and antibiotic resistance genes: status and possible causes. Environ. Int. 159:107047. doi: 10.1016/j.envint.2021.107047, PMID: 34923370

[ref38] Hai Y. Wei M. Y. Wang C. Y. Gu Y. C. Shao C. L. (2021). The intriguing chemistry and biology of sulfur-containing natural products from marine microorganisms (1987-2020). Mar. Life Sci. Tech. 3, 488–518. doi: 10.1007/s42995-021-00101-2, PMID: 37073258 PMC10077240

[ref39] Han J. H. Yang N. Wei S. Z. Jia J. Lin R. Li J. P.. (2022). Dimeric hexylitaconic acids from the marine-derived fungus *Aspergillus welwitschiae* CUGBMF180262. Nat. Prod. Res. 36, 578–585. doi: 10.1080/14786419.2020.1793152, PMID: 32666830

[ref40] Han Y. Q. Zhang Q. Xu W. F. Hai Y. Chao R. Wang C. F.. (2023). Targeted isolation of antitubercular cycloheptapeptides and an unusual pyrroloindoline-containing new analog, asperpyrroindotide a, using LC-MS/MS-based molecular networking. Mar. Life Sci. Tech. 5, 85–93. doi: 10.1007/s42995-022-00157-8, PMID: 36713278 PMC9854410

[ref41] Handayani D. Dwinatrana K. Rustini R. (2022). Antibacterial compound from marine sponge derived fungus *Aspergillus sydowii* DC08. Rasayan J. Chem. 15, 2485–2492. doi: 10.31788/RJC.2022.1546971

[ref42] Handayani D. Rendowati A. Aminah I. Ariantari N. P. Proksch P. (2020). Bioactive compounds from marine sponge derived fungus *Aspergillus unguis* WR8. Rasayan. J. Chem. 13, 2633–2638. doi: 10.31788/RJC.2020.1345781

[ref43] Holland D. C. Prebble D. W. Er S. Hayton J. B. Robertson L. P. Avery V. M.. (2022). α-Synuclein aggregation inhibitory prunolides and a dibrominated β-carboline sulfamate from the ascidian *Synoicum prunum*. J. Nat. Prod. 85, 441–452. doi: 10.1021/acs.jnatprod.1c01172, PMID: 35050597

[ref44] Howden B. P. Giulier S. G. Lung T. W. F. Baines S. L. Sharkey L. K. Lee J. Y. H.. (2023). *Staphylococcus aureus* host interactions and adaptation. Nat. Rev. Microbiol. 21, 380–395. doi: 10.1038/s41579-023-00852-y, PMID: 36707725 PMC9882747

[ref45] Hu Y. Y. Yang M. Zhao J. Liao Z. X. Qi J. Wang X. Z.. (2019). A meroterpenoid isolated from the fungus *Aspergillus* sp. Nat. Prod. Commun. 14:1934578X1987893. doi: 10.1177/1934578X19878933

[ref46] Hu Z. B. Zhu Y. J. Chen J. J. Chen J. Li C. Y. Gao Z. Z.. (2023). Discovery of novel bactericides from *Aspergillus alabamensis* and their antibacterial activity against fish pathogens. J. Agric. Food. Chem. 71, 4298–4305. doi: 10.1021/acs.jafc.2c09141, PMID: 36857464

[ref47] Huang Z. H. Liang X. Gu Q. Ma X. Qi S. H. (2023). Punicesterones A-G, polyhydroxylated mycoecdysteroids from the deep-sea-derived fungus *Aspergillus puniceus* SCSIO z021. Phytochemistry 205:113511. doi: 10.1016/j.phytochem.2022.113511, PMID: 36372238

[ref48] Huang X. M. Wang Y. C. Li G. Y. Shao Z. Z. Xia J. M. Qin J. J.. (2024). Secondary metabolites from the deep-sea derived fungus *Aspergillus terreus* MCCC M28183. Front. Microbiol. 15:1361550. doi: 10.3389/fmicb.2024.1361550, PMID: 38419626 PMC10899347

[ref49] Ibrahim S. R. M. Mohamed S. G. A. Alsaadi B. H. Althubyani M. M. Awari Z. I. Hussein H. G. A.. (2023). Secondary metabolites, biological activities, and industrial and biotechnological importance of *Aspergillus sydowii*. Mar. Drugs 21:441. doi: 10.3390/md21080441, PMID: 37623723 PMC10455642

[ref50] Ikuta K. S. Swetschinski L. R. Aguilar G. R. Sharara F. Mestrovic T. Gray A. P.. (2022). Global mortality associated with 33 bacterial pathogens in 2019: a systematic analysis for the global burden of disease study 2019. Lancet 400, 2221–2248. doi: 10.1016/S0140-6736(22)02185-7, PMID: 36423648 PMC9763654

[ref51] Jeewon R. Aullybux A. A. Puchooa D. Nazurally N. Alrefaei A. F. Zhang Y. (2023). Marine microbial polysaccharides: an untapped resource for biotechnological applications. Mar. Drugs 21:420. doi: 10.3390/md21070420, PMID: 37504951 PMC10381399

[ref52] Jin M. Osman M. Green B. A. Yang Y. F. Ahuja A. Lu Z. Y.. (2023). Evidence for the transmission of antimicrobial resistant bacteria between humans and companion animals: a scoping review. One Health 17:100593. doi: 10.1016/j.onehlt.2023.100593, PMID: 37448771 PMC10336692

[ref53] King A. M. Reid-Yu S. A. Wang W. King D. T. Pascale G. D. Strynadka N. C.. (2014). Aspergillomarasmine A overcomes metallo-*β*-lactamase antibiotic resistance. Nature 510, 503–506. doi: 10.1038/nature13445, PMID: 24965651 PMC4981499

[ref54] Kong F. D. Huang X. L. Ma Q. Y. Xie Q. Y. Wang P. Chen P. W.. (2018). Helvolic acid derivatives with antibacterial activities against *Streptococcus agalactiae* from the marine-derived fungus *Aspergillus fumigatus* HNMF0047. J. Nat. Prod. 81, 1869–1876. doi: 10.1021/acs.jnatprod.8b00382, PMID: 30070829

[ref55] Kumla D. Sousa E. Marengo A. Dethoup T. Pereira J. A. Gales L.. (2021). 1,3-dioxepine and spiropyran derivatives of viomellein and other dimeric naphthopyranones from cultures of *Aspergillus elegans* KUFA0015 and their antibacterial activity. Phytochemistry 181:112575. doi: 10.1016/j.phytochem.2020.112575, PMID: 33166747

[ref56] Lee Y. M. Kim M. J. Li H. Y. Zhang P. Bao B. Q. Lee K. J.. (2013). Marine-derived *Aspergillus* species as a source of bioactive secondary metabolites. Mar. Biotechnol. 15, 499–519. doi: 10.1007/s10126-013-9506-3, PMID: 23709045

[ref57] Lee Y. M. Li H. Y. Hong J. K. Cho H. Y. Bae K. S. Kim M. A.. (2010). Bioactive metabolites from the sponge-derived fungus *Aspergillus versicolor*. Arch. Pharm. Res. 33, 231–235. doi: 10.1007/s12272-010-0207-4, PMID: 20195823

[ref58] Li H. H. Fu Y. Q. Song F. H. (2023). Marine *Aspergillus*: a treasure trove of antimicrobial compounds. Mar. Drugs 21:277. doi: 10.3390/md21050277, PMID: 37233471 PMC10222851

[ref59] Li W. H. Gao Q. Hu Y. J. Shi Y. T. Yan X. J. Ding L. J.. (2023). Dibetanide, a new benzofuran derivative with the rare conjugated triene side chain from a sponge-associated fungus *Aspergillus* species. J. Mol. Struct. 1271:134082. doi: 10.1016/j.molstruc.2022.134082

[ref60] Li J. L. Jiang X. Liu X. P. He C. W. Di Y. X. Lu S. J.. (2019). Antibacterial anthraquinone dimers from marine derived fungus *Aspergillus* sp. Fitoterapia 133, 1–4. doi: 10.1016/j.fitote.2018.11.015, PMID: 30543983

[ref61] Li X. D. Li X. Li X. M. Xu G. M. Zhang P. Meng L. H.. (2016). Tetranorlabdane diterpenoids from the deep sea sediment-derived fungus *Aspergillus wentii* SD-310. Planta Med. 82, 877–881. doi: 10.1055/s-0042-102965, PMID: 27257768

[ref62] Li X. D. Li X. Li X. M. Yin X. L. Wang B. G. (2021). Antimicrobial bisabolane-type sesquiterpenoids from the deep-sea sediment-derived fungus *Aspergillus versicolor* SD-330. Nat. Prod. Res. 35, 4265–4271. doi: 10.1080/14786419.2019.1696792, PMID: 31782317

[ref63] Li S. D. Wei M. Y. Chen G. Y. Lin Y. C. (2012). Two new dihydroisocoumarins from the endophytic fungus *Aspergillus* sp. collected from the South China Sea. Chem. Nat. Compd. 48, 371–373. doi: 10.1007/s10600-012-0254-9

[ref64] Li J. X. Xu Q. H. Shang R. Y. Liu Q. Luo X. C. Lin H. W.. (2023). Aspergetherins A-D, new chlorinated biphenyls with anti-MRSA activity from the marine sponge symbiotic fungus *Aspergillus terreus* 164018. Chem. Biodivers. 20:e202300010. doi: 10.1002/cbdv.202300010 36876631

[ref65] Limbadri S. Luo X. W. Lin X. P. Liao S. R. Wang J. F. Zhou X. F.. (2018). Bioactive novel indole alkaloids and steroids from deep sea-derived fungus *Aspergillus fumigatus* SCSIO 41012. Molecules 23:2379. doi: 10.3390/molecules23092379 30231470 PMC6225233

[ref66] Lin S. X. Li J. Chen W. Z. He J. X. Shi Y. T. Jin H. X.. (2023). A new antibacterial dihydroisocoumarin from the marine sponge-associated fungus *Aspergillus* sp. Chem. Nat. Compd. 59, 246–248. doi: 10.1007/s10600-023-03967-z

[ref67] Lin S. H. Yan Q. X. Zhang Y. Wu T. Z. Zou Z. B. Liu Q. M.. (2023). Citriquinolinones a and B: rare isoquinolinone-embedded citrinin analogues and related metabolites from the deep-sea-derived *Aspergillus versicolor* 170217. Mar. Drugs 21:504. doi: 10.3390/md21100504, PMID: 37888439 PMC10608187

[ref68] Liu Y. Ding L. J. He J. X. Zhang Z. M. Deng Y. T. He S.. (2021). A new antibacterial chromone from a marine sponge-associated fungus *Aspergillus* sp. LS57. Fitoterapia 154:105004. doi: 10.1016/j.fitote.2021.105004, PMID: 34339802

[ref69] Liu Y. Ding L. J. Shi Y. T. Yan X. J. Wu B. He S. (2022). Molecular networking-driven discovery of antibacterial perinadines, new tetracyclic alkaloids from the marine sponge-derived fungus *Aspergillus* sp. ACS Omega 7, 9909–9916. doi: 10.1021/acsomega.2c00402, PMID: 35350304 PMC8945076

[ref70] Liu Y. Li X. M. Meng L. H. Wang B. G. (2015). Polyketides from the marine mangrove-derived fungus *Aspergillus ochraceus* MA-15 and their activity against aquatic pathogenic bacteria. Phytochem. Lett. 12, 232–236. doi: 10.1016/j.phytol.2015.04.009

[ref71] Liu X. H. Miao F. P. Liang X. R. Ji N. Y. (2014). Ergosteroid derivatives from an algicolous strain of *Aspergillus ustus*. *Nat*. *Prod*. *Res*. 28, 1182–1186. doi: 10.1080/14786419.2014.923996, PMID: 24896666

[ref72] Liu X. H. Miao F. P. Qiao M. F. Cichewicz R. H. Ji N. Y. (2013). Terretonin, ophiobolin, and drimane terpenes with absolute configurations from an algicolous *Aspergillus ustus*. RSC Adv. 3, 588–595. doi: 10.1039/C2RA22701K

[ref73] Liu W. Wang L. P. Wang B. Xu Y. C. Zhu G. L. Lan M. M.. (2019). Diketopiperazine and diphenylether derivatives from marine algae-derived *Aspergillus versicolor* OUCMDZ-2738 by epigenetic activation. Mar. Drugs 17:6. doi: 10.3390/md17010006, PMID: 30583513 PMC6356248

[ref74] Liu C. M. Yao F. H. Lu X. H. Zhang X. X. Luo L. X. Liang X.. (2022). Isoquinoline alkaloids as protein tyrosine phosphatase inhibitors from a deep-sea-derived fungus *Aspergillus puniceus*. Mar. Drugs 20:78. doi: 10.3390/md20010078, PMID: 35049933 PMC8781450

[ref75] Lu C. J. Tang Z. Z. Su Z. W. Li H. Y. Zhang G. S. Gao C. H.. (2023). Secondary metabolites from marine-derived fungus *Aspergillus carneus* GXIMD00519. Rec. Nat. Prod. 17, 343–351. doi: 10.25135/rnp.355.2207.2518

[ref76] Luo X. W. Zhou X. F. Lin X. P. Qin X. C. Zhang T. Y. Wang J. F.. (2017). Antituberculosis compounds from a deep-sea-derived fungus *Aspergillus* sp. SCSIO Ind09F01. Nat. Prod. Res. 31, 1958–1962. doi: 10.1080/14786419.2016.1266353, PMID: 28068839

[ref77] Lv H. W. Wang K. B. Xue Y. X. Chen J. Su H. B. Zhang J. K.. (2021). Three new metabolites from the marine-derived fungus *Aspergillus* sp. WHUF03110. Nat. Prod. Commun. 16:1934578X2110550. doi: 10.1177/1934578X211055009

[ref78] Lv H. W. Zhang J. K. Xue Y. X. Li S. W. Sun X. Y. Jia J.. (2022). Two new austocystin analogs from the marine-derived fungus *Aspergillus* sp. WHUF05236. Chem. Biodivers. 19:e202200207. doi: 10.1002/cbdv.202200207, PMID: 35419971

[ref79] Machado F. P. Kumla D. Pereira J. A. Sousa E. Dethoup T. Freitas-Silva J.. (2021). Prenylated phenylbutyrolactones from cultures of a marine sponge-associated fungus *Aspergillus flavipes* KUFA1152. Phytochemistry 185:112709. doi: 10.1016/j.phytochem.2021.112709, PMID: 33636575

[ref80] Machado F. P. Rodrigues I. C. Gales L. Pereira J. A. Costa P. M. Dethoup T.. (2022). New alkylpyridinium anthraquinone, isocoumarin, c-glucosyl resorcinol derivative and prenylated pyranoxanthones from the culture of a marine sponge-associated fungus, *Aspergillus stellatus* KUFA 2017. Mar. Drugs 20:672. doi: 10.3390/md20110672, PMID: 36354995 PMC9696483

[ref81] Meng Q. Y. Guo X. Wu J. S. Liu D. Gu Y. C. Huang J.. (2022). Prenylated notoamide-type alkaloids isolated from the fungus *Aspergillus sclerotiorum* and their inhibition of NLRP3 inflammasome activation and antibacterial activities. Phytochemistry 203:113424. doi: 10.1016/j.phytochem.2022.113424, PMID: 36063866

[ref82] Miao F. P. Li X. D. Liu X. H. Cichewicz R. H. Ji N. Y. (2012). Secondary metabolites from an algicolous *Aspergillus versicolor* strain. Mar. Drugs 10, 131–139. doi: 10.3390/md10010131, PMID: 22363226 PMC3280527

[ref83] Neuhaus G. F. Adpressa D. A. Bruhn T. Loesgen S. (2019). Polyketides from marine-derived *Aspergillus porosus*: challenges and opportunities for determining absolute configuration. J. Nat. Prod. 82, 2780–2789. doi: 10.1021/acs.jnatprod.9b00416, PMID: 31557023

[ref84] Okeke I. N. de Kraker M. E. A. Van Boeckel T. P. Kumar C. K. Schmitt H. Gales A. C.. (2024). The scope of the antimicrobial resistance challenge. Lancet 403, 2426–2438. doi: 10.1016/S0140-6736(24)00876-6 38797176

[ref85] Orfali R. Aboseada M. A. Abdel-Wahab N. M. Hassan H. M. Perveen S. Ameen F.. (2021). Recent updates on the bioactive compounds of the marine-derived genus *Aspergillus*. RSC Adv. 11, 17116–17150. doi: 10.1039/D1RA01359A, PMID: 35479707 PMC9033173

[ref86] Peng Q. Y. Cai J. Long J. Y. Yang B. Lin X. P. Wang J. F.. (2021). New azaphthalide and phthalide derivatives from the marine coral-derived fungus *Aspergillus* sp. SCSIO41405. Phytochem. Lett. 43, 94–97. doi: 10.1016/J.PHYTOL.2021.03.019

[ref87] Peng Q. Y. Chen W. H. Lin X. P. Xiao J. Liu Y. H. Zhou X. F. (2022). Butenolides from the coral-derived fungus *Aspergillius terreus* SCSIO41404. Mar. Drugs 20:212. doi: 10.3390/md20030212, PMID: 35323511 PMC8955524

[ref88] Pinedo-Rivilla C. Aleu J. Duran-Patron R. (2022). Cryptic metabolites from marine-derived microorganisms using OSMAC and epigenetic approaches. Mar. Drugs 20:84. doi: 10.3390/md20020084, PMID: 35200614 PMC8879561

[ref89] Prestinaci F. Pezzotti P. Pantosti A. (2015). Antimicrobial resistance: a global multifaceted phenomenon. Pathog. Glob. Health 109, 309–318. doi: 10.1179/2047773215Y.0000000030 26343252 PMC4768623

[ref90] Ren J. M. Yang J. K. Zhu H. J. Cao F. (2020). Bioactive steroids from the marine-derived fungus *Aspergillus flavus* JK07-1. Chem. Nat. Compd. 56, 945–947. doi: 10.1007/s10600-020-03195-9

[ref91] Saetang P. Rukachaisirikul V. Phongpaichit S. Preedanon S. Sakayaroj J. Hadsadee S.. (2021). Antibacterial and antifungal polyketides from the fungus *Aspergillus unguis* PSU-MF16. J. Nat. Prod. 84, 1498–1506. doi: 10.1021/acs.jnatprod.0c01308 33861594

[ref92] Song F. H. Lin R. Yang N. Jia J. Wei S. Z. Han J. H.. (2021). Antibacterial secondary metabolites from marine-derived fungus *Aspergillus* sp. IMCASMF180035. Antibiotics 10:377. doi: 10.3390/antibiotics10040377, PMID: 33916658 PMC8066187

[ref93] Song Z. J. Liu Y. Gao J. Y. Hu J. S. He H. T. Dai S. W.. (2021). Antitubercular metabolites from the marine-derived fungus strain *Aspergillus fumigatus* MF029. Nat. Prod. Res. 35, 2647–2654. doi: 10.1080/14786419.2019.1660331, PMID: 34414849

[ref94] Song F. H. Liu X. R. Guo H. Ren B. Chen C. X. Piggott A. M. (2012). Brevianamides with antitubercular potential from a marine-derived isolate of *Aspergillus versicolor*. Org. Lett. 14, 4770–4773. doi: 10.1021/ol302051x, PMID: 22963079

[ref95] Song F. H. Ren B. Chen C. X. Yu K. Liu X. R. Zhang Y. H.. (2014). Three new sterigmatocystin analogues from marine-derived fungus *Aspergillus versicolor* MF359. Appl. Microbiol. Biotechnol. 98, 3753–3758. doi: 10.1007/s00253-013-5409-5 24458562

[ref96] Sun C. Z. Ha Y. R. Liu X. Wang N. Lian X. Y. Zhang Z. Z. (2024). Isolation and structure elucidation of new metabolites from the mariana-trench-associated fungus *Aspergillus* sp. SY2601. Molecules 29:459. doi: 10.3390/molecules29020459, PMID: 38257372 PMC10819015

[ref97] Sun K. L. Li Y. Guo L. Wang L. Liu P. P. Zhu W. M. (2014). Indole diterpenoids and isocoumarin from the fungus, *Aspergillus flavus*, isolated from the prawn, *Penaeus vannamei*. Mar. Drugs 12, 3970–3981. doi: 10.3390/md12073970, PMID: 24983640 PMC4113809

[ref98] Sun L. X. Wang H. N. Yan M. C. Sai C. M. Zhang Z. (2022). Research advances of bioactive sesquiterpenoids isolated from marine-derived *Aspergillus* sp. Molecules 27:7376. doi: 10.3390/molecules27217376 36364202 PMC9659078

[ref99] Sun C. X. Zhang Z. P. Ren Z. L. Yu L. Zhou H. Han Y. X.. (2020). Antibacterial cyclic tripeptides from Antarctica-sponge-derived fungus *Aspergillus insulicola* HDN151418. Mar. Drugs 18:532. doi: 10.3390/md18110532, PMID: 33114712 PMC7694092

[ref100] Thi H. A. N. Mai A. N. Thi T. H. V. Thi M. H. D. Van C. P. Thanh X. D.. (2023). Antimicrobial activity of depsidones and macrocyclic peptides isolated from marine sponge-derived fungus *Aspergillus nidulans* M256. Chem. Biodivers. 20:e202301660. doi: 10.1002/cbdv.202301660, PMID: 37957128

[ref101] Tian Y. Q. Lin S. T. Kumaravel K. Zhou H. Wang S. Y. Liu Y. H. (2018). Polyketide-derived metabolites from the sponge-derived fungus *Aspergillus* sp. F40. Phytochem. Lett. 27, 74–77. doi: 10.1016/j.phytol.2018.06.009

[ref102] Tuan C. D. Van Hung N. Minh L. T. H. Lien H. T. H. Chae J. W. Yun H. Y.. (2022). A new indole glucoside and other constituents from the sea cucumber-derived *Aspergillus fumigatus* M580 and their biological activities. Rec. Nat. Prod. 16, 633–638. doi: 10.25135/rnp.310.2110.2248

[ref103] Ukwatta K. M. Lawrence J. L. Wijayarathne C. D. (2020). Antimicrobial, anti-cancer, anti-filarial and anti-inflammatory activities of cowabenzophenone a extracted from the endophytic fungus *Aspergillus terreus* isolated from a mangrove plant *Bruguiera gymnorrhyza*. Mycology 11, 297–305. doi: 10.1080/21501203.2019.1707722, PMID: 33329925 PMC7723023

[ref104] Wallis R. S. O’Garra A. Sher A. Wack A. (2023). Host-directed immunotherapy of viral and bacterial infections: past, present and future. Nat. Rev. Immunol. 23, 121–133. doi: 10.1038/s41577-022-00734-z, PMID: 35672482 PMC9171745

[ref105] Wang W. Y. Chen R. X. Luo Z. H. Wang W. Chen J. M. (2018). Antimicrobial activity and molecular docking studies of a novel anthraquinone from a marine-derived fungus *Aspergillus versicolor*. Nat. Prod. Res. 32, 558–563. doi: 10.1080/14786419.2017.1329732, PMID: 28511613

[ref106] Wang Q. Y. Chen H. P. Wu K. Y. Li X. Y. Liu J. K. (2022). Antibacterial and *β*-amyloid precursor protein-cleaving enzyme 1 inhibitory polyketides from the fungus *Aspergillus chevalieri*. Front. Microbiol. 13:1051281. doi: 10.3389/fmicb.2022.1051281, PMID: 36483193 PMC9722750

[ref107] Wang K. W. Ding P. (2018). New bioactive metabolites from the marine-derived fungi *Aspergillus*. *Mini*-*Rev. Med. Chem.* 18, 1072–1094. doi: 10.2174/1389557518666180305160856 29512458

[ref108] Wang W. Y. Gao M. L. Luo Z. H. Liao Y. Y. Zhang B. B. Ke W. Q.. (2019). Secondary metabolites isolated from the deep sea-derived fungus *Aspergillus sydowii* C1-S01-A7. Nat. Prod. Res. 33, 3077–3082. doi: 10.1080/14786419.2018.1519561, PMID: 30251547

[ref109] Wang C. Y. Liu X. H. Zheng Y. Y. Ning X. Y. Zhang Y. H. Fu X. M.. (2022). 2,5-diketopiperazines from a sponge-derived fungus *Aspergillus sclerotiorum*. Front. Microbiol. 13:808532. doi: 10.3389/fmicb.2022.808532, PMID: 35668768 PMC9164150

[ref110] Wang C. Sarotti A. M. Zaman K. H. Ahammad U. Wu X. H. Cao S. G. (2021). New alkaloids from a Hawaiian fungal strain *Aspergillus felis* FM324. Front. Chem. 9:724617. doi: 10.3389/fchem.2021.724617, PMID: 34434921 PMC8380829

[ref111] Wen H. M. Zhang Y. W. Feng F. J. Huang G. B. Lv Y. H. Zhang Z. Y.. (2024). Antibacterial oxygenated ergostane-type steroids produced by the marine sponge-derived fungus *Aspergillus* sp. J. Asian Nat. Prod. Res. 26, 548–554. doi: 10.1080/10286020.2023.2259317, PMID: 37712720

[ref112] Wu J. S. Shi X. H. Yao G. S. Shao C. L. Fu X. M. Zhang X. L.. (2020a). New thiodiketopiperazine and 3,4-dihydroisocoumarin derivatives from the marine-derived fungus *Aspergillus terreus*. Mar. Drugs 18:132. doi: 10.3390/md18030132, PMID: 32110865 PMC7143538

[ref113] Wu J. S. Shi X. H. Zhang Y. H. Shao C. L. Fu X. M. Li X.. (2020b). Benzyl furanones and pyrones from the marine-derived fungus *Aspergillus terreus* induced by chemical epigenetic modification. Molecules 25:3927. doi: 10.3390/molecules25173927 32867374 PMC7503933

[ref114] Wu J. Shui H. Zhang M. K. Zeng Y. D. Zheng M. X. Zhu K. K.. (2023). Aculeaxanthones A-E, new xanthones from the marine-derived fungus *Aspergillus* aculeatinus WHUF0198. Front. Microbiol. 14:1138830. doi: 10.3389/fmicb.2023.1138830, PMID: 36922969 PMC10008875

[ref115] Xu P. Ding L. J. Wei J. X. Li Q. Gui M. J. He X. P.. (2020). A new aquatic pathogen inhibitor produced by the marine fungus *Aspergillus* sp. LS116. Aquaculture 520:734670. doi: 10.1016/j.aquaculture.2019.734670

[ref116] Xu X. L. Han J. H. Zhang X. W. Xu W. Yang J. P. Song F. H. (2024). Investigation on the chemical constituents of the marine-derived fungus strain *Aspergillus brunneoviolaceus* MF180246. Nat. Prod. Res. 38, 1369–1374. doi: 10.1080/14786419.2022.2144300, PMID: 36369790

[ref117] Xu W. F. Wu N. N. Wu Y. W. Qi Y. X. Wei M. Y. Pineda L. M.. (2022). Structure modification, antialgal, antiplasmodial, and toxic evaluations of a series of new marine-derived 14-membered resorcylic acid lactone derivatives. Mar. Life Sci. Tech. 4, 88–97. doi: 10.1007/s42995-021-00103-0, PMID: 37073350 PMC10077203

[ref118] Xu X. L. Yang H. J. Xu H. T. Yin L. Y. Chen Z. K. Shen H. H. (2018). Diphenyl ethers from a marine-derived isolate of *Aspergillus* sp. CUGB-F046. Nat. Prod. Res. 32, 821–825. doi: 10.1080/14786419.2017.1363754, PMID: 28826261

[ref119] Xu K. Yuan X. L. Li C. Li A. X. (2020). Recent discovery of heterocyclic alkaloids from marine-derived *Aspergillus* species. Mar. Drugs 18:54. doi: 10.3390/md18010054 31947564 PMC7024353

[ref120] Xu L. L. Zhang C. C. Zhu X. Y. Cao F. Zhu H. J. (2017). Bioactive phenyl ether derivatives from the marine-derived fungus *Aspergillus carneus*. Nat. Prod. Res. 31, 1875–1879. doi: 10.1080/14786419.2016.1263848, PMID: 27917659

[ref121] Xuan J. Q. Feng W. G. Wang J. Y. Wang R. C. Zhang B. W. Bo L. T.. (2023). Antimicrobial peptides for combating drug-resistant bacterial infections. Drug Resist. Update 68:100954. doi: 10.1016/j.drup.2023.100954 36905712

[ref122] Xue J. J. Guo X. P. Xu G. X. Chen X. Jiao L. H. Tang X. X. (2024). Discovery, identification, and mode of action of phenolics from marine-derived fungus *Aspergillus ustus* as antibacterial wilt agents. J. Agr. Food. Chem. 72, 2989–2996. doi: 10.1021/acs.jafc.3c07826, PMID: 38214488

[ref123] Yan L. H. Du F. Y. Li X. M. Yang S. Q. Wang B. G. Li X. (2023). Antibacterial indole diketopiperazine alkaloids from the deep-sea cold seep-derived fungus *Aspergillus chevalieri*. Mar. Drugs 21:195. doi: 10.3390/md21030195, PMID: 36976244 PMC10059655

[ref124] Yang J. Gong L. Z. Guo M. M. Jiang Y. Ding Y. Wang Z. J.. (2021). Bioactive indole diketopiperazine alkaloids from the marine endophytic fungus *Aspergillus* sp. YJ191021. Mar. Drugs 19:157. doi: 10.3390/md19030157 33802820 PMC8002477

[ref125] Yang S. Q. Li X. M. Li X. Li H. L. Meng L. H. Wang B. G. (2018b). New citrinin analogues produced by coculture of the marine algal-derived endophytic fungal strains *Aspergillus sydowii* EN-534 and *Penicillium citrinum* EN-535. Phytochem. Lett. 25, 191–195. doi: 10.1016/j.phytol.2018.04.023

[ref126] Yang S. Q. Li X. M. Xu G. M. Li X. An C. Y. Wang B. G. (2018a). Antibacterial anthraquinone derivatives isolated from a mangrove-derived endophytic fungus *Aspergillus nidulans* by ethanol stress strategy. J. Antibiot. 71, 778–784. doi: 10.1038/s41429-018-0063-x, PMID: 29717199

[ref127] Yang G. H. Sandjo L. Yun K. Leutou A. S. Kim G. D. Choi H. D.. (2011). Flavusides a and B, antibacterial cerebrosides from the marine-derived fungus *Aspergillus flavus*. Chem. Pharm. Bull. 59, 1174–1177. doi: 10.1248/cpb.59.1174, PMID: 21881265

[ref128] Yang M. Y. Yang J. K. Yang J. K. Hu L. D. Zhu H. J. Cao F. (2018). New oxygenated steroid from the marine-derived fungus *Aspergillus flavus*. Nat. Prod. Commun. 13:1934578X1801300. doi: 10.1177/1934578X1801300807

[ref129] Yang X. Yu H. J. Ren J. W. Cai L. Xu L. J. Liu L. (2023). Sulfoxide-containing bisabolane sesquiterpenoids with antimicrobial and nematicidal activities from the marine-derived fungus *Aspergillus sydowii* LW09. J. Fungi 9:347. doi: 10.3390/jof9030347, PMID: 36983515 PMC10057145

[ref130] Ye W. X. Zhao M. R. Wang L. Jiang X. D. Zhang W. J. Zhang C. S.. (2022). Isolation, identification, and bioactive metabolites of coral-derived fungus *Aspergillus* sp. SCSIO 40435 from the South China Sea. Weishengwu Xuebao 62, 1819–1831. doi: 10.13343/j.cnki.wsxb.20210568

[ref131] Yu G. H. Wu G. W. Sun Z. C. Zhang X. M. Che Q. Gu Q. Q.. (2018). Cytotoxic tetrahydroxanthone dimers from the mangrove-associated fungus *Aspergillus versicolor* HDN1009. Mar. Drugs 16:335. doi: 10.3390/md16090335, PMID: 30223483 PMC6164687

[ref132] Yu H. J. Xue Y. X. Hong K. Jia J. Bi H. K. Xu L. J.. (2022). Secondary metabolites from a mangrove-derived fungus *Aspergillus* sp. WHUF0343. Weishengwu Xuebao 62, 2658–2670. doi: 10.13343/j.cnki.wsxb.20210655

[ref133] Yurchenko A. N. Girich E. V. Yurchenko E. A. (2021). Metabolites of marine sediment-derived fungi: actual trends of biological activity studies. Mar. Drugs 19:88. doi: 10.3390/md19020088, PMID: 33557071 PMC7913796

[ref134] Zang Z. M. Yang W. C. Cui H. Cai R. L. Li C. Y. Zou G.. (2022). Two antimicrobial heterodimeric tetrahydroxanthones with a 7,7′-linkage from mangrove endophytic fungus *Aspergillus flavus* QQYZ. Molecules 27:2691. doi: 10.3390/molecules27092691, PMID: 35566042 PMC9103106

[ref135] Zeng Q. Chen Y. C. Wang J. F. Shi X. F. Che Y. H. Chen X. Y.. (2022a). Diverse secondary metabolites from the coral-derived fungus *Aspergillus hiratsukae* SCSIO 5Bn_1_003. Mar. Drugs 20:150. doi: 10.3390/md20020150, PMID: 35200679 PMC8877224

[ref136] Zeng Q. Zhong W. M. Chen Y. C. Xiang Y. Chen X. Y. Tian X. P.. (2020b). A new butenolide derivative from the deep-sea fungus *Aspergillus terreus* SCSIO FZQ028. Nat. Prod. Res. 34, 1984–1991. doi: 10.1080/14786419.2019.1569658, PMID: 30721083

[ref137] Zhang J. Gao L. L. Lin H. T. Liang Y. You M. N. Ding L. J.. (2024). Discovery of antibacterial compounds against *Xanthomonas citri* subsp. citri from a marine fungus *Aspergillus terreus* SCSIO 41202 and the mode of action. J. Agric. Food. Chem. 72, 12596–12606. doi: 10.1021/acs.jafc.4c02769, PMID: 38771666

[ref138] Zhang Y. H. Hou X. M. Yu M. L. Wang C. Y. (2019). Secondary metabolites and their bioactivities from the gorgonian-derived fungus *Aspergillus versicolor*. *Chem*. *Nat*. *Compd*. 55, 327–330. doi: 10.1007/s10600-019-02680-0

[ref139] Zhang Y. T. Li Z. C. Huang B. Y. Liu K. Peng S. Liu X. M.. (2022). Anti-osteoclastogenic and antibacterial effects of chlorinated polyketides from the beibu gulf coral-derived fungus *Aspergillus unguis* GXIMD02505. Mar. Drugs 20:178. doi: 10.3390/md20030178, PMID: 35323477 PMC8956104

[ref140] Zhang Y. Li X. M. Wang B. G. (2012). Anthraquinone derivatives produced by marine-derived fungus *Aspergillus versicolor* EN-7. Biosci. Biotechnol. Biochem. 76, 1774–1776. doi: 10.1271/bbb.120047, PMID: 22972319

[ref141] Zhang L. Qiu P. P. Ding L. J. Li Q. Song J. J. Han Z. W.. (2020). A new antibacterial chlorinated amino acid derivative from the sponge-derived fungus *Aspergillus* sp. LS53. Chem. Nat. Compd. 56, 109–111. doi: 10.1007/s10600-020-02955-x

[ref142] Zhang R. Wang H. F. Chen B. S. Dai H. Q. Sun J. Z. Han J. J.. (2022). Discovery of anti-MRSA secondary metabolites from a marine-derived fungus *Aspergillus fumigatus*. Mar. Drugs 20:302. doi: 10.3390/md20050302, PMID: 35621953 PMC9146929

[ref143] Zhang Y. H. Xu Y. Wang C. Y. Cao F. (2020). Alkaloids and sesquiterpenoids from the marine-derived fungus *Aspergillus versicolor*. Chem. Nat. Compd. 56, 971–973. doi: 10.1007/s10600-020-03205-w

[ref144] Zheng C. J. Shao C. L. Wu L. Y. Chen M. Wang K. L. Zhao D. L.. (2013). Bioactive phenylalanine derivatives and cytochalasins from the soft coral-derived fungus, *Aspergillus elegans*. Mar. Drugs 11, 2054–2068. doi: 10.3390/md11062054, PMID: 23752358 PMC3721221

[ref145] Zhou Y. M. Debbab A. Wray V. Lin W. H. Schulz B. Trepos R.. (2014). Marine bacterial inhibitors from the sponge-derived fungus *Aspergillus* sp. Tetrahedron Lett. 55, 2789–2792. doi: 10.1016/j.tetlet.2014.02.062

[ref146] Zhu S. H. Chang Y. M. Su M. Z. Yao L. G. Li S. W. Wang H.. (2024). Nine new antibacterial diterpenes and steroids from the South China Sea soft coral *Lobophytum catalai Tixier-Durivault*. Mar. Drugs 22:50. doi: 10.3390/md22010050, PMID: 38276652 PMC10817416

[ref147] Zhu F. Chen G. Y. Chen X. Huang M. Z. Wan X. Q. (2011). Aspergicin, a new antibacterial alkaloid produced by mixed fermentation of two marine-derived mangrove epiphytic fungi. Chem. Nat. Compd. 47, 767–769. doi: 10.1007/s10600-011-0053-8

[ref148] Zhu A. Zhang X. W. Zhang M. Li W. Ma Z. Y. Zhu H. J.. (2018). Aspergixanthones I-K, new anti-vibrio prenylxanthones from the marine-derived fungus *Aspergillus* sp. ZA-01. Mar. Drugs 16:312. doi: 10.3390/md16090312, PMID: 30181432 PMC6165128

